# Photocatalytic Hydrogenation of an N_2_‑Derived Re^V^ Imido Complex

**DOI:** 10.1021/jacsau.5c00525

**Published:** 2025-09-25

**Authors:** Katharina Oelschlegel, Myron Heinz, Sandipan Maji, Robert Naumann, Yannis F. S. Höhle, Niyaz Alizadeh, Markus Finger, Matthias Otte, Katja Heinze, Max C. Holthausen, Sven Schneider

**Affiliations:** † Institut für Anorganische Chemie and International Center for Advanced Studies of Energy Conversion, 9375 Georg-August-Universität Göttingen, 37077 Göttingen, Germany; ‡ Institut für Anorganische und Analytische Chemie, 9173 Goethe-Universität Frankfurt am Main, 60438 Frankfurt am Main, Germany; § Department of Chemistry, 9182 Johannes Gutenberg University, 55128 Mainz, Germany

**Keywords:** N_2_ fixation, hydrogenation, photocatalysis, transient absorption spectroscopy, computational chemistry

## Abstract

Direct hydrogenation of N_2_ at ambient conditions lags behind the recent advances in molecular N_2_ (electro-)­fixation. In fact, molecular platforms that couple N_2_ and H_2_ activation remain rare. Inspired by recent photocatalytic approaches, we report here the photochemical hydrogenation of an N_2_-derived rhenium­(V) benzoylimido complex. Self-sensitized photolysis with sacrificial 1,4-cyclohexadiene gave benzamide in a high yield. In turn, a new iridium­(III) porphyrinato hydrido photocatalyst allowed for quantitative photohydrogenation via light-driven net hydride transfer. Transient absorption spectroscopy and computations support a light-driven, stepwise hydride transfer mechanism that is enabled by relativistic effects. This approach provides a new strategy for the reductive transformation of N_2_ into nitrogenous products beyond ammonia.

## Graphical abstract

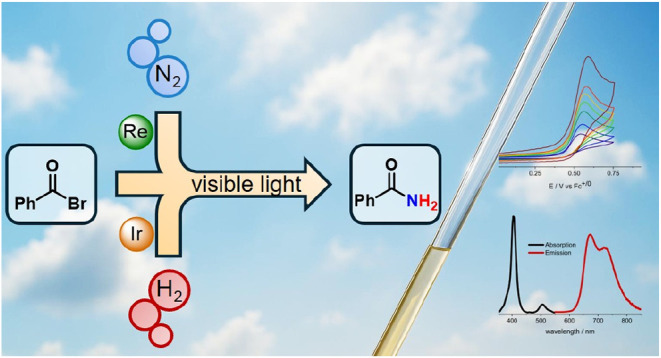

While industrial N_2_ hydrogenation (N_2_ + 3 H_2_ → 2 NH_3_) is operated at a huge scale (>150 Mt·a^–1^), homogeneous variants define an unachieved goal.^1–3^ One key challenge is set by the small driving force (Δ*G*_gas_^0^ = 3.9 kcal·mol^–1^).^4^ Thus, even moderate chemical overpotentials imposed by intermediates with weak N–H bonds easily shut down catalysis.^5–7^ The recent advances in molecular N_2_ electrofixation were fueled by the introduction of proton-coupled electron transfer (PCET) reagents (e.g., SmI_2_/H_2_O) and mediators that offset the necessity for strong reductants.^8–11^ Alternatively, photoredox catalysis was utilized for light-driven N_2_ fixation with sacrificial H-donors.^12,13^ Similarly, successful strategies are yet to be developed for H_2_ as a reductant.

Pioneering work by the Fryzuk and Chirik groups demonstrated H_2_ addition along Zr–N bonds of highly activated N_2_ (Scheme 1).^14,15^ Hidai and Nishibayashi utilized nonclassical H_2_-complexes to protonate coordinated N_2_.^16^ H_2_ heterolysis was later expanded toward Mo sulfido clusters and frustrated Lewis pairs.^17–19^ Hou and co-workers introduced Ti alkyl precursors that split N_2_ upon hydrogenolysis with concomitant N–H bond formation.^20–22^ Related multinuclear N_2_/H_2_ activation was also reported for Cr, U, and Fe (Scheme 1) precursors.^23–26^ However, ammonia yields in these cases ultimately remained limited.

**Scheme 1:**
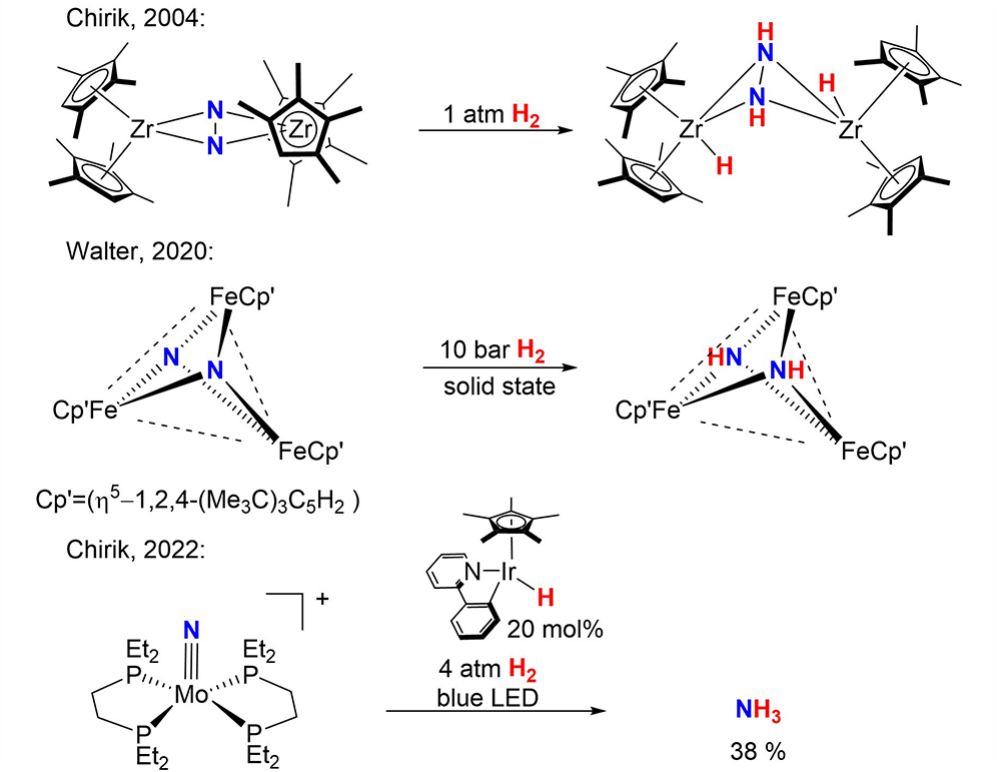
Selected Examples for Concomitant N_2_/H_2_ (Photo-)­activation^15,26,27^

Photocatalysis is also a promising strategy to offset bottlenecks in the N_2_ hydrogenation. Chirik recently introduced Ir hydrido photocatalysts for the light-driven hydrogenation of an N_2_-derived nitride (Scheme 1).^27,28^ Here, this approach is expanded toward organic nitrogenous products.^29,30^ We previously reported the electroreduction of the N_2_-derived Re nitride **1** to benzonitrile in the presence of protons and benzoyl bromide (Scheme 2).^31,32^ C–N coupling with the electrophile allowed for bypassing a Re^IV^ imido intermediate with a weak N–H bond. We now demonstrate the use of H_2_ as a reductant, aided by molecular photocatalysis.

**Scheme 2:**
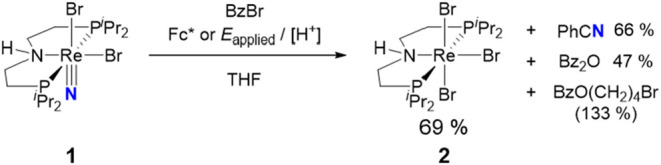
(Electro-)­chemical Generation of Benzonitrile from an N_2_-Derived Nitrido Complex^32^

We first computationally evaluated the thermochemical framework for stepwise H-transfer to the N_2_-derived imido complex [Re^V^{NC­(O)­Ph}­Br_2_(HPNP)]^+^ (**3** ^**+**^), which is easily prepared from **1**.^32^ [Re^IV^{NC­(OH)­Ph}­Br_2_(HPNP)]^+^ (**4** ^**+**^) and [Re^III^{OC­(NH_2_)­Ph}­Br_2_(HPNP)]^+^ (**5** ^**+**^) were obtained as the lowest-energy configurations (Chart 1). All quantum chemical results were obtained by employing the ONIOM­(CCSD­(T)-F12:scLH22t) method except for the Ir–Ir bond dissociation free energy (BDFE), which was obtained at the scLH22t level. Open-shell Re and Ir complexes were corrected for contributions from spin–orbit coupling (SOC) to the thermochemistry by multireference computations with perturbational treatment of SOC (CASSCF/NEVPT2/QDPT; cf. Supporting Information (SI) for details). Note that the unusual molecular structure of *trans*-**5** ^**+**^ was confirmed by single-crystal X-ray diffraction (SC-XRD; Figure S64). According to the computed BDFEs of **4** ^**+**^ and **5** ^**+**^ vs. that of H_2_,^35^ the hydrogenation of **3^+^** should be exergonic by 15 kcal·mol^–1^. However, no direct reaction of **3** ^**+**^ with H_2_ is observed, indicative of a significant kinetic barrier under the reaction conditions.

**Chart 1:**
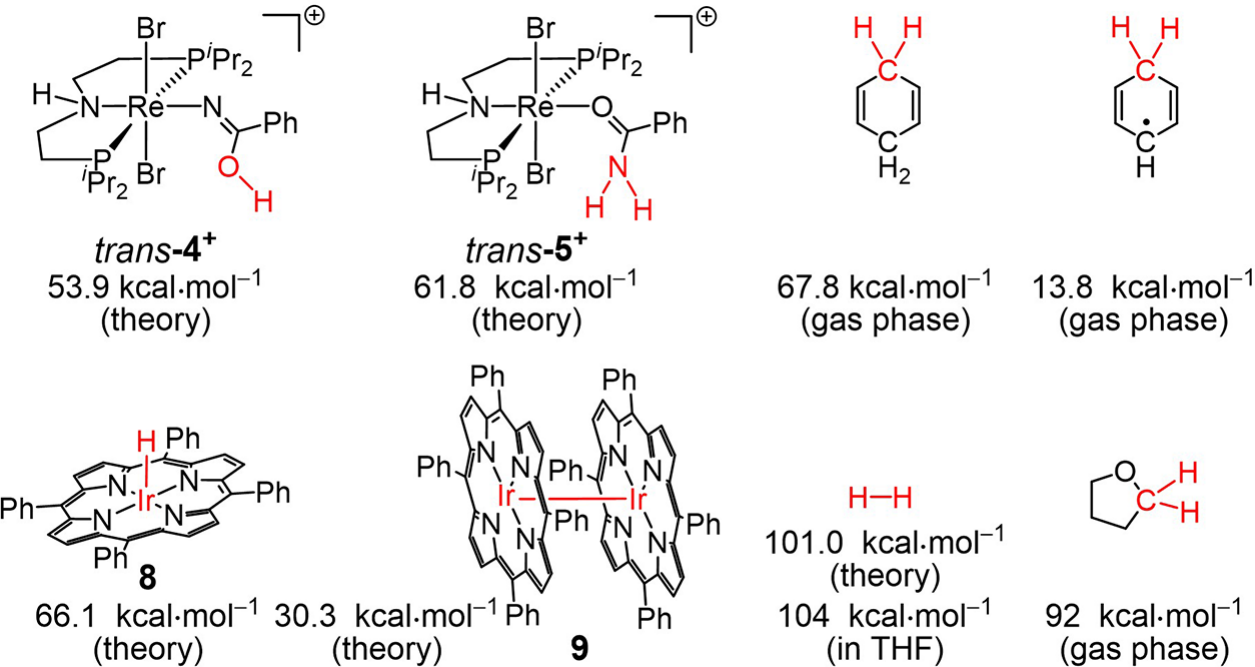
Selected BDFE Data of Relevant Species with Respective Bonds Marked in Red^33–35^

Direct photoactivation was, therefore, first considered. Photolysis of **3** ^**+**^ near the lowest-energy absorption maximum (λ_max_ = 405 nm; Figure S72) with a blue LED (λ_max_ = 456 nm) in THF-*d*_8_ results in quantitative decay over 24 h (Figures S29–S34). Even in the absence of an additional reductant like H_2_, the benzonitrile complex [ReBr_2_(NCPh)­(HPNP)]^+^ (**6** ^**+**^, Scheme 3, *top*) is obtained as the main product (90%) with a low quantum yield (*Φ* = 0.11 ± 0.08%). Complex **6** ^**+**^ exhibits strongly shifted yet sharp NMR resonances (δ­(^31^P) = −2526 ppm), as is typically obtained for octahedral Re^III^ complexes due to temperature-independent paramagnetism and rapid electronic relaxation.^36–39^ SC-XRD confirmed the *trans*-dibromido configuration in the solid state (Figure S63). Addition of tetrabutylammonium bromide after photolysis results in the quantitative release of benzonitrile and **2**, i.e., the precursor for reductive N_2_ splitting.^32^ In addition to that, NMR spectroscopic examination indicated the formation of structurally ill-defined ring-opening products of 2-hydroxytetrahydrofuran (see Figure S34). Thus, THF acts as an oxygen atom acceptor in the unsensitized photolysis.^40^

**Scheme 3:**
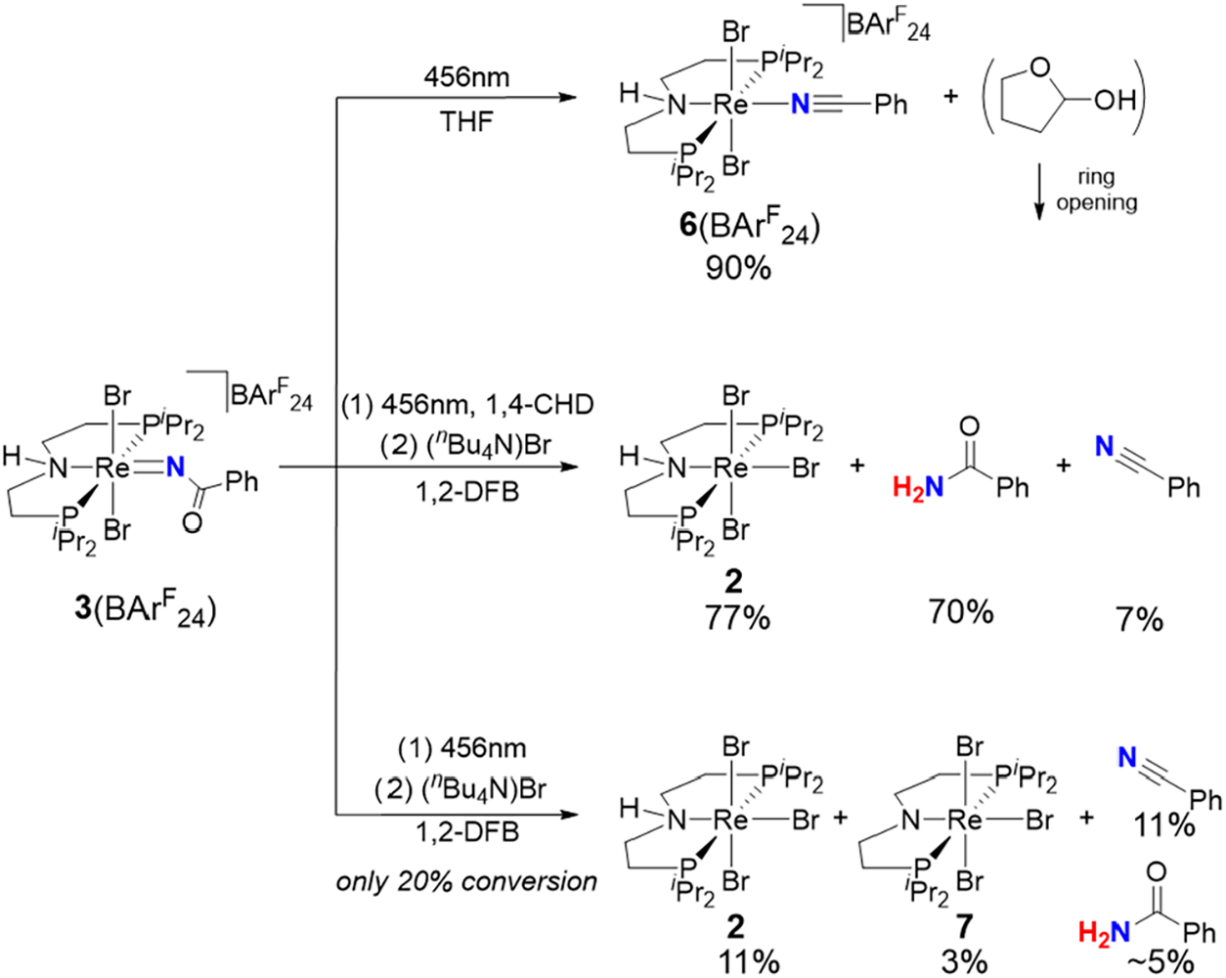
Unsensitized Photochemical Transformations of 3­(BAr^F^_24_) in THF and 1,2-DFB

We therefore turned to 1,2-difluorobenzene (1,2-DFB) as a chemically more inert solvent (Scheme 3, *bottom*). Accordingly, a significantly reduced decay of **3** ^**+**^ was observed even after a prolonged photolysis over 24 h (λ_max_ = 456 nm). Subsequent addition of (^*n*^Bu_4_N)Br gave [Re^IV^Br_3_(PNP)] (**7**) and [Re^III^Br_3_(HPNP)] (**2**) in 3 and 11% yield, respectively, in addition to benzonitrile (11%) and some benzamide (∼5%). The product distribution thus suggests that the metal ion and the N–H proton of the pincer ligand serve as e^–^ and H^+^ donors, respectively. Photolysis of **3** ^**+**^ with 1,4-cyclohexadiene (1,4-CHD, 10 equiv) as a sacrificial reductant and the subsequent addition of (^*n*^Bu_4_N)Br (Scheme 3, *center*) gave the nitrogen transfer product **2** in high yield (77%, *Φ* = 0.02 ± 0.008%). Furthermore, the corresponding amounts of free benzamide (70%) and benzonitrile (7%) are obtained. Comparison of the BDFE_O–H_ of **4** ^**+**^ (Chart 1) vs the BDFE_C–H_ of 1,4-CHD or THF^34,35^ shows that the first H-transfer to **3** ^**+**^ is thermochemically unfeasible and should therefore be a light-driven process.

Imido complex **3** ^**+**^ does not luminesce upon excitation at 400 nm in 1,2-DFB. Transient absorption spectroscopy (TAS) confirmed a rapid excited state decay within around 10 ps (Figure S56). A weak photoproduct absorption signature remained, which is in line with the low quantum yield. TD-DFT calculations for the transition of **3** ^**+**^ at 405 nm indicated a predominant charge transfer character from the bromide ligands into the {ReNC­(O)} core, suggesting Re–Br homolysis as the likely decay path that initiates H atom transfer processes with solvent, CHD, or the pincer ligand, respectively.^41^ In fact, the photoproduct(s) exhibit absorptions in the region of solvated bromine atoms (Figure S62),^42^ but unequivocal assignment is hampered due to superimposed bands from the remaining Re fragment. Nevertheless, the TAS experiments did not rule out this scenario for unsensitized nitrile release.

We next turned to H_2_ as a reductant. Photolysis of **3**(BAr^F^_24_) in 1,2-DFB under an atmosphere of H_2_ (1 bar) gave the same results as those under Ar (vide infra). Molecular photocatalysis was therefore considered, taking into account the unsensitized decay of **3** ^**+**^ when excited below λ = 456 nm. Note that Chirik’s iridium­(III) hydride (Scheme 1) exhibits MLCT absorption maxima well below 500 nm. We therefore turned to porphyrin ligands, which often exhibit absorptions beyond 500 nm.^43,44^ The iridium­(III) tetraphenylporphyrinato hydrido complex [IrH­(TPP)] (**8**; Scheme 4) was synthesized according to a modified procedure for a structurally related compound (see SI).^45^ While thermal H-transfer from iridium­(III) porphyrin hydrides has previously been examined,^46,47^ their photochemistry remains surprisingly unexplored.

**Scheme 4:**
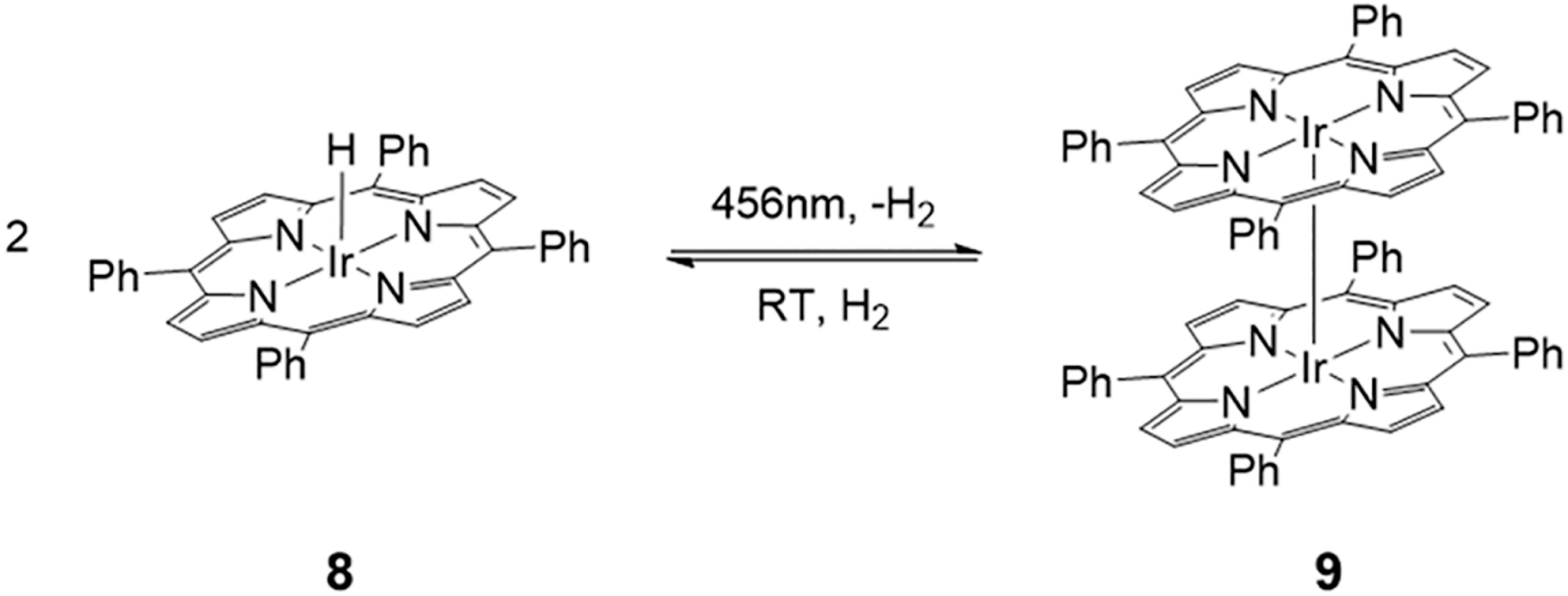
Photochemical Hydrogen Evolution and Thermal Hydrogen Addition of **8** and **9**

Electrochemical characterization of **8** by cyclic voltammetry (Figure 1) reveals an oxidation at *E*_pf_ = +0.54 V, which remains irreversible at scan rates of up to 20 V·s^–1^. Chronoamperometry (Figure S25) confirmed a 2-electron process. We tentatively attribute the irreversibility to coupled proton loss. This assumption is supported by thermochemical considerations: As judged from the potential and the computed Ir–H BDFE of **8** (Chart 1), high acidity of the oxidized state can be expected. In analogy to other Ir porphyrinato hydrides,^46,47^ **8** undergoes facile H-transfer to 2,2,6,6-tetramethylpiperidine-1-oxyl (TEMPO; BDFE_O–H_(TEMPO–H) = 65.5 kcal·mol^–1^)^35^ with subsequent dimerization to the diamagnetic bisiridium­(II) complex [Ir­(TPP)]_2_ (**9**; Scheme 4), as confirmed by DOSY NMR spectroscopy (*D* = 4.55 × 10^–6^ cm^2^·s^–1^). Our thermochemical computations (Chart 1) confirmed that the reaction with TEMPO is driven by the dimerization step. Importantly, **9** in turn adds H_2_ (1 bar) to quantitatively restore hydride **8** (Scheme 4) within a nearly thermoneutral step (Δ*G* ^0^ = −1.0 kcal·mol^–1^), which is ideal for catalytic hydrogen transfer.

**Figure 1:**
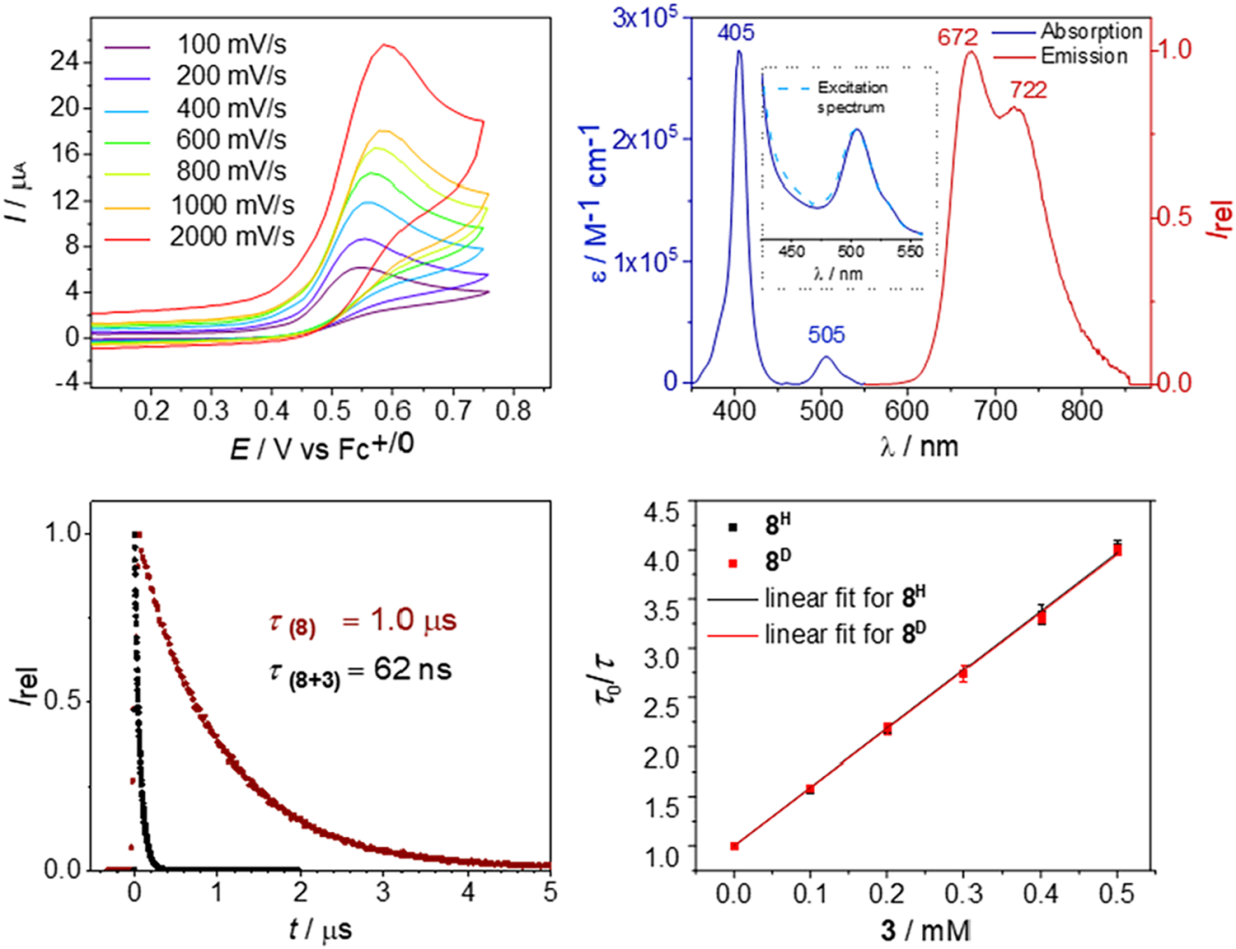
*Top left*: Electrochemical characterization of **8**. *Top right*: Electronic absorption (blue) and emission (red) spectra of **8** in 1,2-DFB at room temperature with comparison of the absorption and excitation (dashed) spectra in the Q-band region as the inset. *Bottom left*: Decay of the emission intensity of **8** (red) and **8** (0.33 mM) and **3**(BAr^F^_24_) (2.5 mM) (black) in 1,2-DFB at room temperature. *Bottom right*: Stern–Volmer plot for lifetime quenching (672 nm) of **8** (black) and **8^D^** (red) (both 1 × 10^–5^ M) with **3**(BAr^F^_24_) (solid lines: linear fit).

In addition to the Soret band (405 nm), the electronic absorption spectrum of **8** in 1,2-DFB exhibits a Q-band at 505 nm (Figure 1).^44^ Excitation of either transition leads to strong phosphorescence at 672 nm with a shoulder at 722 nm, which can be attributed to a vibronic progression.^44,48–50^ Time-correlated single-photon counting measurements at room temperature gave a lifetime of the phosphorescent state (τ = 1.0 μs) that is well suited for bimolecular photocatalysis.^51^ Transient absorption spectroscopy revealed all of the typical features for a porphyrin-centered ^3^(π,π*) excited state with a strong absorption around 450 nm, a broad feature below 500 nm that extends into the near-infrared (NIR) region, and a distinct NIR band at λ_max_ = 870 nm (Figure S57).^52^ Prolonged photolysis of **8** at λ = 456 or 525 nm leads to H_2_ evolution and formation of dimer **9** (Scheme 4), confirming that H_2_ elimination can be driven by visible light. Estimation of the excited state Ir–H bond strength from a Rehm–Weller cycle (21 kcal·mol^–1^)^53^ suggests ample driving force for H-transfer to our N_2_-derived rhenium precursor **3**(BAr^F^_24_).

Motivated by the chemical and photophysical properties of **8**, we targeted the photocatalytic hydrogenation of **3** ^**+**^. Photolysis of **3** ^**+**^ and **8** (2 mol %) with green light (λ = 525 nm) in 1,2-difluorobenzene (r.t.) under an atmosphere of H_2_ (1 bar) initially leads to one new rhenium product (δ_P_ = −2117 ppm), while photocatalyst **8** is not consumed. NMR spectroscopic characterization supports the assignment to the rhenium­(III) *cis*-dibromido hydrogenation product [ReBr_2_{OC­(NH_2_)­Ph}­(HPNP)]^+^ (*cis*-**5** ^**+**^). In solution, *cis*-**5** ^**+**^ isomerizes over the course of several hours to the *trans*-dibromo isomer *trans*-**5** ^**+**^ (δ_P_ = −1882 ppm), which was independently synthesized and fully characterized, including SC-XRD (Figure S64). The addition of (^*n*^Bu_4_N)Br as a bromide source allows for the release of benzamide in high yield over both steps (92% yield), in addition to small amounts of benzonitrile (6%). In turn, the precursor to N_2_ splitting, tribromide **2**, is restored in the corresponding amounts (98%). This reaction thus closes a synthetic cycle for the light-driven benzamide synthesis from dinitrogen using H_2_ as a reductant (Scheme 5).

**Scheme 5:**
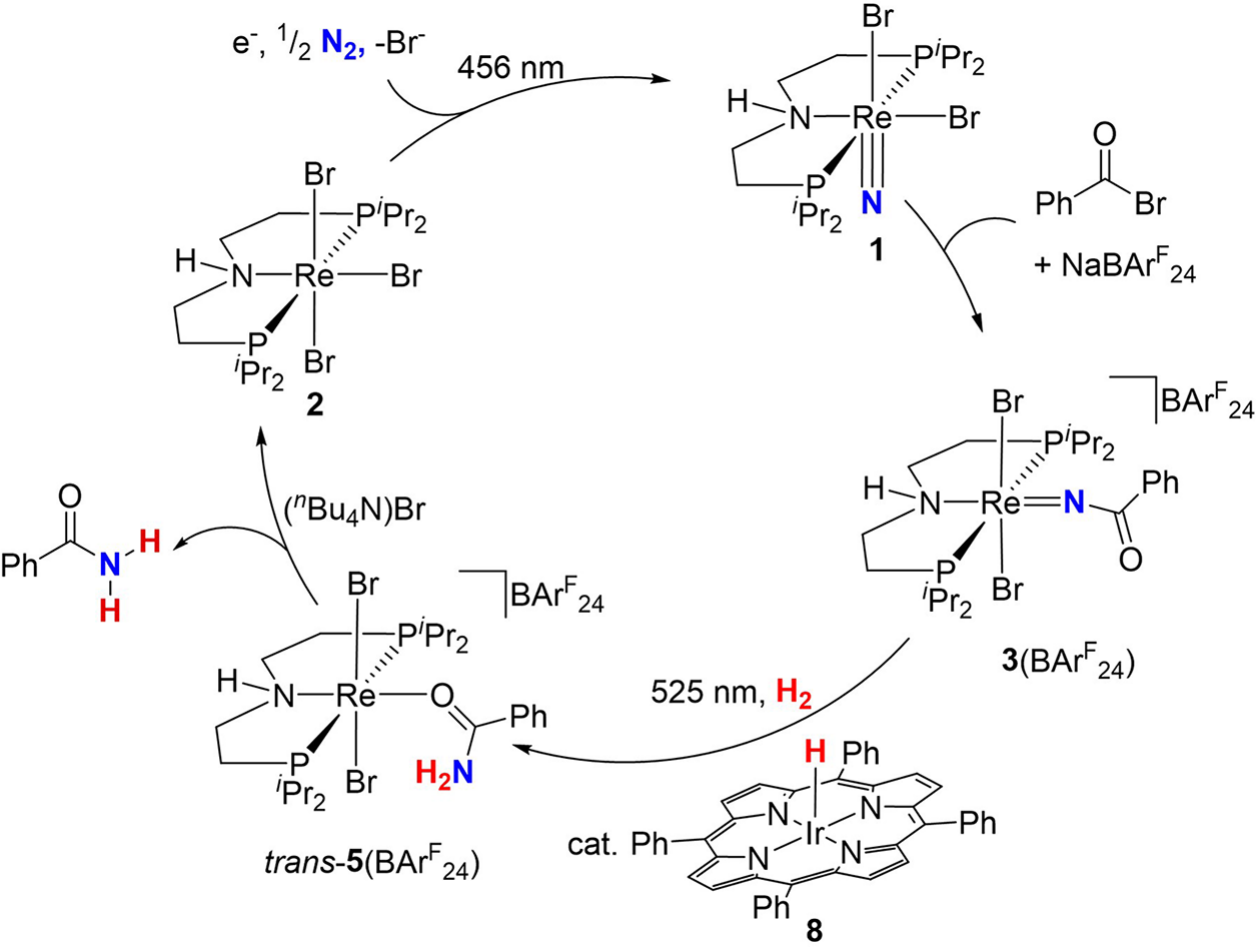
Synthetic Cycle for Benzamide Synthesis from N_2_, H_2_, and Benzoyl Bromide via Photoelectrochemical N_2_ Splitting and Photocatalytic Hydrogenation^a^

a Only the thermodynamically more stable *trans* isomer of **5** Is shown for clarity.

^15^N and ^2^H isotopic labeling confirmed the N_2_-derived imido ligand and dihydrogen as N- and H-sources, respectively (Figures S49–S52). No reaction is observed in the dark or in the absence of the photocatalyst. Stoichiometric photolysis (525 nm) of **3** ^**+**^ and **8** (2 equiv) under argon led to an approximately 1:1 conversion, yet with low selectivity. In addition to **2** (23%), several unidentified Re compounds are observed, along with benzamide (28%), no benzonitrile, and one unknown porphyrin product (**A**). A control reaction of dimer **9** with **3** ^**+**^ in the dark also gave **8** and **A** (1:1), suggesting that Ir^II^ can thermally undergo H-abstraction from the parent rhenium imide in the absence of the reductant H_2_.

Next, photocatalytic hydrogenation was examined in more detail. The quantum yield (*Φ* = 0.11 ± 0.10%) does not indicate a light-induced radical chain. Direct excited state reactivity of 8* with **3** ^**+**^ was therefore examined. **3** ^**+**^ efficiently quenches the emission of 8* with a dynamic quenching constant of *k*_q_ = 6.4 × 10^9^ M^–1^·s^–1^ (Figure 1). Identical rates were obtained for the iridium deuteride **8** ^**D**^ (Figure 1), disfavoring initial proton transfer or concerted PCET.^54^ From the irreversible oxidation wave and the emission energy, we estimated an upper limit for the excited-state redox potential of around E*­(**8** ^**+**^/8*) ≲ −1.1 V.^53^ Thus, the reduction of imido complex **3** ^**+**^ (*E*_1/2_ = −0.69 V) by 8* should be thermochemically feasible. However, alternative energy transfer cannot be excluded. The lowest computed triplet state of **3** ^**+**^ is at 27.6 kcal·mol^–1^ (Table S9) above the singlet ground state (vertically) and well below *E*_00_(8*) ≈ 45.2 kcal·mol^–1^.

TAS experiments confirmed the quenching of 8* by **3** ^**+**^ (Figures 2, *left*, S59, and S60). After full decay of 8*, the spectra revealed distinct absorption bands of the quenching product(s) between 500 and 800 nm. Comparison with TD-DFT computed spectra of potential iridium products showed good agreement with [Ir­(TPP)]^+^ in its triplet ground state (Figure 2, *right*). In contrast, no resemblance was found for ^2^[Ir­(H)­(TPP)]^+^ or ^2^[Ir­(TPP)] (Figures S67 and S69). Furthermore, the Re complexes generally exhibit extinction coefficients that are more than an order of magnitude lower, and the spectra of isolated **3** and **4** (Figures S20 and S21) do not exhibit absorptions beyond 500 (**4**) and 600 nm (**3**), respectively. The spectroscopic results thus suggest formal hydride transfer upon reaction of 8* with **3** ^**+**^, presumably as a stepwise process as judged from the KIE of unity (see above).

**Figure 2:**
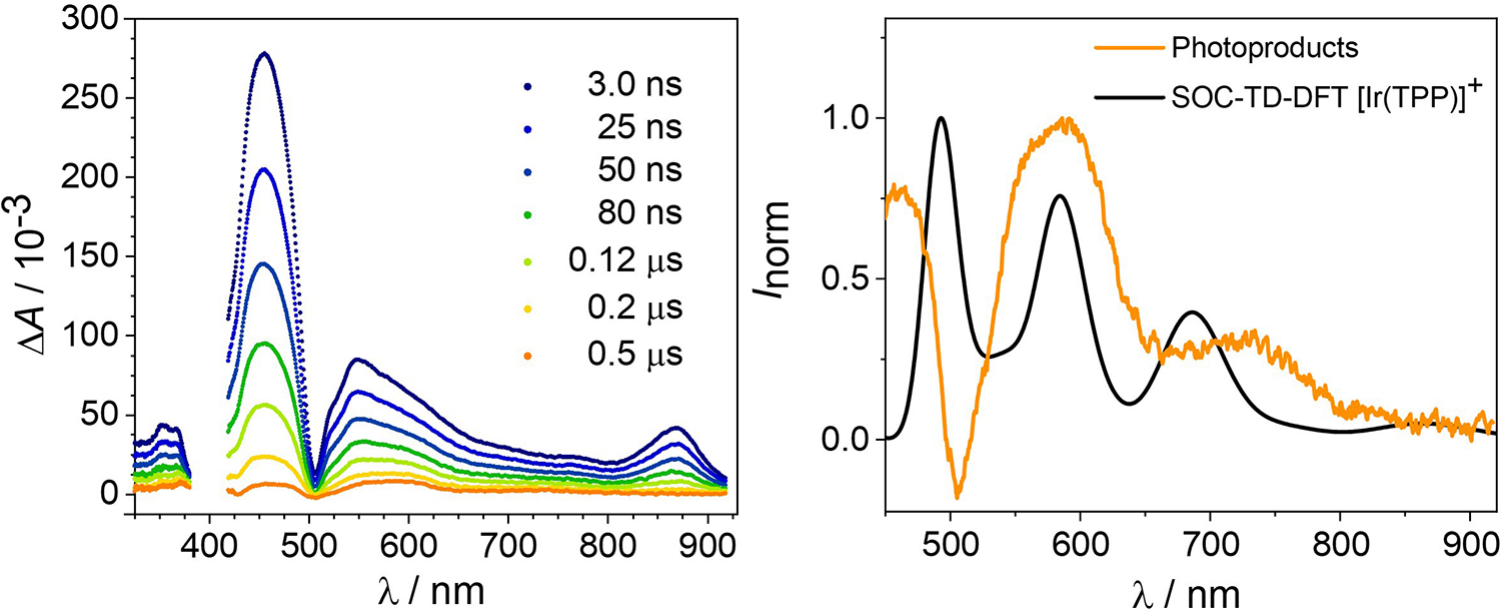
*Left*: Transient absorption spectra of **8** (0.33 mM) and **3** ^**+**^ (2.5 mM) in 1,2-DFB were obtained after 505 nm excitation. *Right*: Averaged (0.5–1.5 μs) TA spectrum (orange) superimposed with the (unshifted) SOC-TD-DFT calculated spectrum of [Ir­(TPP)]^+^ (black; bands broadened with fwhm = 1200 cm^–1^). The minimum in the TA spectrum originates from the bleach of the Q-band of **8** at 505 nm.

Thermochemical considerations support this proposal (Scheme 6). As pointed out above, ET from 8* to **3** ^**+**^ is downhill by Δ*G* ^0^ = −9.5 kcal·mol^–1^. For the subsequent formal hydrogen atom transfer from **8** ^**+**^ to **3**, an overall slightly exergonic reaction free energy of Δ*G* ^0^ = −1.4 kcal·mol^–1^ is computed, assuming relaxation of **3** or the final Re^III^ amido product **4** to their more stable *cis*-configuration. We note that the explicit treatment of SOC contributions to the thermochemistry is crucial for the computational treatment. Srnec et al. showed that SOC can shift the redox potentials of heavy 5d ions by up to around 400 mV (9.2 kcal·mol^–1^) if one of the redox states exhibits a (near) degenerate spin ground state.^55,56^ For H-transfer from **8** ^**+**^ to **3**, our computations found a large SOC contribution to the driving force of around 10 kcal·mol^–1^. It originates in additive contributions of both 5d redox couples, i.e., the conversion of doublet **8** ^**+**^ and doublet **3** to the triplet species **10** ^**+**^ and **4** (see SI for details).

**Scheme 6:**
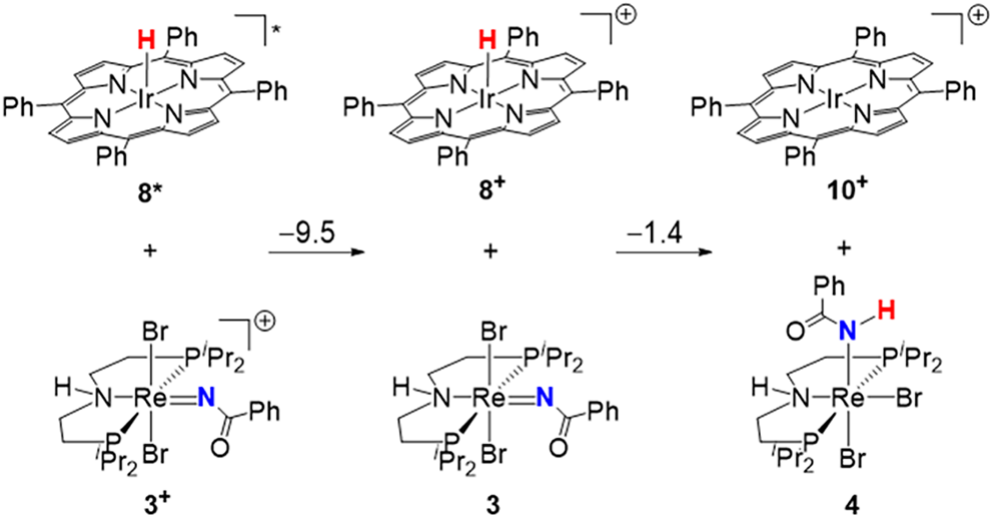
Thermochemistry for Stepwise Hydride Transfer upon Quenching of 8* with **3** ^**+**^ (the Numbers on the Arrows Denote Free Energies in kcal·mol^–1^)

In summary, we reported the unsensitized and sensitized photochemical reduction of an N_2_-derived imido complex **3**(BAr^F^_24_). Direct excitation of **3** ^**+**^ with blue light enables the release of benzonitrile or benzamide upon activation of THF or cyclohexadiene as sacrificial reductant, which is probably initiated by light-induced Re–Br homolysis. Light-driven hydrogenation of **3^+^**, which is thermally inaccessible, could be realized with **8** as a new PCET photocatalyst. The ^3^(π,π*) excited state lifetime of **8** and the near thermoneutral regeneration from dimer **9** with H_2_ provide an excellent framework for photocatalytic hydrogenation. Transient absorption spectroscopy supports that quenching of 8* by imido complex **3** ^**+**^ results in net hydride transfer. As judged by electrochemical, kinetic (KIE), and computational data, this reaction presumably is a stepwise process with initial light-driven ET and subsequent thermal PCET. Notably, the driving force for the latter relies on large relativistic effects (SOC) on the thermochemistry of the two heavy metal ions, Ir and Re. In consequence, combining the photocatalytic hydrogenation with photochemical N_2_ splitting and C–N bond formation enabled a light-driven, stepwise protocol for the conversion of N_2_ to benzamide.

## Supplementary Material





## Notes

Experimental details and spectroscopic, crystallographic, and computational data (PDF)
XYZ coordinates (TXT)
