# Supplementary material for: Photocatalytic Hydrogenation of an N2‑Derived ReV Imido Complex
Source: JACS Au. 2025 Sep 25;5(10):4706–13. doi: 10.1021/jacsau.5c00525 (PMC12569705; doi:10.1021/jacsau.5c00525)
Supplement: Supplementary file 1 [file au5c00525_si_001.pdf]

Supporting Information

Photocatalytic Hydrogenation of an N_2-derived Re^V Imido complex

Katharina Oelschlegel,[†] Myron Heinz,[‡] Sandipan Maji,[†] Robert Naumann,[¶] Yannis F. S. Höhle,[†] Niyaz Alizadeh,[†] Markus Finger,[†] Matthias Otte,[†] Katja Heinze,[¶,*] Max C. Holthausen,[‡,*] and Sven Schneider[†,*]

[†] Institut für Anorganische Chemie and International Center for Advanced Studies of Energy Conversion, Georg-August-Universität Göttingen, 37077 Göttingen, Germany.

[‡] Institut für Anorganische und Analytische Chemie, Goethe-Universität Frankfurt am Main, 60438 Frankfurt am Main, Germany.

[¶] Department of Chemistry, Johannes Gutenberg University, 55128 Mainz, Germany.

*E-Mail: katja.heinze@uni-mainz.de, max.holthausen@chemie.uni-frankfurt.de , sven.schneider@chemie.uni-goettingen.de

Table of Contents

1 Experimental 3

1.1 Materials and Synthetic Methods 3

1.2 Analytical Methods 3

1.3 Syntheses 5

1.3.1 Synthesis of [Ir(TPP)Cl(CO)] 5

1.3.2 Synthesis of [Ir(TPP)H] **8** 6

1.3.3 Synthesis of $[Ir(TPP)]_2$ (**9**) 10

1.3.4 Photolysis of [Ir(TPP)H] (**8**) 12

1.3.5 Synthesis of $[ReBr_2(HPNP)(NCPh)]^+$ (**6**$^+$) 13

1.3.6 Synthesis of $[ReBr_2\{OC(NH_2)Ph\}(HPNP)]^+$ (*trans*-**5** ($BAr^F{}_{24}$)) 15

1.3.7 UV-vis Spectra of **3** and **4** 17

1.4 Electrochemical Investigations 18

1.4.1 Electrochemical examination of **3**($BAr^F{}_{24}$) in 1,2-difluorobenzene 18

1.4.2 Electrochemical examination of **8** in 1,2-difluorobenzene 19

1.5 Excited state thermochemical data of **8** 21

1.5.1 Estimation of excited state thermochemical data of **8**. 21

2 Self-sensitized photochemical reactions of 3^+ 22

2.1 Photolysis of **3**$^+$ in THF 22

2.2 Photolysis of **3**$^+$ in 1,2-difluorobenzene 26

2.3 Photolysis of **3**$^+$ and 1,4-CHD in 1,2-difluorobenzene 27

2.4 Photolysis of **3**$^+$ and **8** in 1,2-difluorobenzene 29

2.5 Reaction of **3**$^+$ and **9** in 1,2-difluorobenzene 31

3 Photocatalytic hydrogenation 31

3.1 Synthetic Procedures 31

3.2 Isotopic Labelling 33

^{15}N Labelling 33

^{2}H Labelling 34

3.3 Stern-Volmer Analysis 35

3.4 Quantum Yields 37

3.4.1 Quantum yield of the photoconversion of **3**($BAr^F{}_{24}$) to **6**($BAr^F{}_{24}$) 37

3.4.2 Quantum yield of the photoconversion of **3**($BAr^F{}_{24}$) with 1,4-CHD 37

3.4.3 Quantum yield of the photohydrogenation of **3**($BAr^F{}_{24}$) with photocatalyst **8** 37

4 Transient Absorption Spectroscopy 38

4.1 Transient Absorption Spectroscopy of **3**$^+$ 38

4.2 Transient Absorption Spectroscopy of **8** 38

4.3 Transient Absorption Spectroscopy of **8** with **3**$^{+}$ 39
4.4 Transient Absorption Spectroscopy of Br_2 in 1,2-difluorobenzene 41
5 Crystallographic Details 41
5.1 Crystal Structure of **6**(BAr^F_{24})·C_5H_{12} 42
5.2 Crystal Structure of *trans*-**5**(BAr^F_{24}) 49
6. Computational Details 55
6.1 UV/vis spectrum of **3**$^{+}$ 74
References 75

1 Experimental

1.1 Materials and Synthetic Methods

All experiments were carried out under inert conditions (argon, Linde 5.0) using standard Schlenk and glove-box techniques (argon atmosphere). Glassware was oven dried at 120 °C for at least 2 hours prior to use and allowed to cool under vacuum. All solvents were purchased in HPLC quality (SIGMA ALDRICH) and dried using an MBRAUN Solvent Purification System. THF, toluene and benzene were additionally dried over Na/K-alloy, 1,2-difluorobenzene was dried over CaH_2 and distilled by trap-to-trap transfer in vacuo and degassed via three freeze-pump-thaw cycles. Deuterated solvents were obtained from EURISOTOP GmbH and dried over NaK-alloy (C_6D_6, d_8-THF, d_8-Tol), distilled by trap-to-trap transfer *in vacuo*, and degassed by three *freeze-pump-thaw* cycles, respectively. H_2 (≥99.999%, Linde) was purified by a high capacity moisture filter (ZPure™ H_2O Filters, Pure Gas Products) prior to use. D_2 gas (SIGMA ALDRICH, 99.6 % D) was used without further purification. [Re(NBz)(^{H}PNP)]BAr^F_{24} (**3**(BAr^F_{24})), H_2(TPP), [$Re^{IV}Br_3$(PNP)] (**7**), [Re(NC(O)Ph)(^{H}PNP)] (**3**), [Re(NHC(O)Ph)(^{H}PNP)] (**4**), were synthesized according to published procedures.[1,2,3] The synthesis of [Ir(TPP)Cl(CO)] (see below) was adapted from the synthesis of structurally related [Ir(TTP)Cl(CO)] (TTP = tetrakis(*p*-tolyl)porphyrin).[4,] [Ir(TPP)H] (**8**) was synthesized according to a modified procedure for [Ir(TTP)H].[5] Bromine was degassed and distilled under Argon prior to the measurements. All other chemicals were purchased from chemical vendors and used as received unless otherwise stated.

1.2 Analytical Methods

NMR

NMR spectra were recorded in J-Young tubes on Bruker Avance III HD 300, Avance III HD 400, Avance Neo 400, Avance III HD 500, or Avance Neo 600 at a temperature of 298 K. ^{1}H NMR spectra were referenced to the residual solvent signal (C_6D_6: δ_H = 7.16 ppm; d_8-THF: δ_H = 3.58 ppm; d_8-Tol: δ_H = 2.09 ppm) as internal standard. Chemical shifts for ^{13}C, ^{15}N, ^{31}P, and ^{2}H are given vs. Me_4Si, $MeNO_2$, H_3PO_4, and $(CD_3)_4Si$, respectively. All heteronuclei were referenced via the proton spectrum by using the Ξ value, as recommended by IUPAC.[6] Signal multiplicities are abbreviated as s (singlet), d (doublet), t (triplet), q (quartet), quint (quintet), m (multiplet), dd (doublet of doublets), dt (doublet of triplets), td (triplet of doublets), vt (virtual triplet), or br (broad). MestReNova 14.3 (Mestrelab Research S.L., Santiago de Compostela, ESP) was used for analysis of NMR spectra.

Elemental Analyses

Elemental analyses were obtained from the Analytisches Labor, Georg-August-Universität (Göttingen, Germany) using an Elementar Vario EL 3 analyzer.

Mass Spectrometry

HR-ESI-MS (Bruker maXis QTOF), LIFDI-MS (JEOL AccuTOF JMS-T100GCV; inert conditions) spectra were measured by the Zentrale Massenabteilung, Fakultät für Chemie, Georg-August-Universität, Göttingen.

UV-vis Spectroscopy

UV-vis spectra were recorded on a Varian CARY300 Scan or an Agilent Cary 8454 spectrometer with VT unit (+100 to –195 °C; USP-203 Series, Unisoku, Osaka, JPN) and magnetic stirrer using quartz cuvettes with airtight caps. All UV-vis samples were prepared in a glovebox and transferred out of the glovebox prior to the measurement.

Infrared Spectroscopy

IR spectra were recorded using a Bruker ALPHA FT-IR spectrometer with Platinum ATR, Nicolet Summit X with Everest Diamond ATR module, or a ThermoFisher Scientific Nicolet iS10 FT-IR spectrometer with liquid sample holder (1 mm pathlength).

Cyclic Voltammetry

Cyclic voltammograms were measured with a Gamry 600 reference potentiostat in a 0.2 M [^{n}Bu$_4$N][PF$_6$] solution with glassy carbon working electrode, Pt counter electrode and Ag pseudo-reference electrode. Original spectra were referenced against the $[Fe(C_5H_5)_2]^{+/0}$ couple. The internal resistance was compensated by 90 % of the uncompensated value.

Photolysis Setup

Photolysis experiments were carried out using Kessil PR160L LEDs: 427 (max. 45 W), 456nm (max. 50 W), 525nm (max. 44 W), while keeping the sample at room temperature by a water bath. Irradiation inside the NMR spectrometer was carried out by using a commercial setup from Mountain Photonics (Landsberg am Lech, GER), consisting of a 420 nm LED and a fiber optic, both from Prizmatix (Holon, IL). The fiber optic was sanded at the lower 40 mm, allowing irradiation of the sample around the fiber optic. Instead of a J-Young NMR tube, a combination of inner tube (3 mm), surrounding the fiber optic, and outer tube (5 mm), containing the sample solution, was used. The inner tube is inserted into the outer tube through a screw-cap septum, which is further air-tightened by parafilm.

Emission Spectroscopy

For steady-state emission and excitation spectroscopy, a Fluorolog 3-22 instrument (Horiba Jobin-Yvon) was used. Lifetimes were measured using pulsed light for TCSPC (time correlated single photon counting, Edinburgh Instruments) generated with a NanoLED 510 nm from Horiba. All samples were prepared in a glovebox in 10x10 mm fluorescence quartz cuvette (Hellma, 200-2500mm) and transferred out of the glovebox prior to the measurement. Emission decay was fitted using Origin's single exponential decay function.

Steady-state emission spectra and emission decay curves were measured with a *FLS1000 luminescence spectrometer* from *Edinburgh Instruments* equipped with a cooled a photomultiplier detector *PMT-980*. A xenon arc lamp Xe2 (450 W) was used for excitation in steady-state measurements. Emission decay curves were recorded employing a pulsed diode laser *VPL-450* (λ_{exc} = 451.3 nm) or a white light pulsed supercontinuum laser NKT-FIU-6 SuperK Fianium (λ_{exc} = 505 nm) from *NKT Photonics.*

fs-Transient absorption experiments were conducted using a *Helios* pump-probe setup from *Ultrafast Systems* paired with a regeneratively amplified 1030 nm laser (*Pharos, Light Conversion*, 1030 nm, < 175 fs, 2 mJ). The effective laser repetition rate of 2 kHz was set via an internal pulse picker. A small portion of the 1030 nm fundamental was directed to the optical delay line and was subsequently used

to generate broadband probe light by focusing the beam onto a sapphire for measurements in the Vis/NIR range (450 nm – 900 nm). The pump pulse was generated with an optical parametric amplifier (*Apollo Y*, *Ultrafast Systems*) and the beam diameter was adjusted to 100 μm – 150 μm at the sample to assure homogeneous excitation of the observation volume, which is defined by the probe diameter (ca. 20 μm). The samples were measured under argon atmosphere in a quartz cuvette (*d* = 1 mm). To correct artefacts due to emission and scattering, a second chopper was installed along the probe beam. Preprocessing of the data, including chirp and baseline correction, has been performed using the *Surface Xplorer 4.3.0* software from *Ultrafast Systems*.

ns-Transient absorption experiments were carried out using a modified version of the described fs-transient absorption spectroscopy setup. For this purpose, the *EOS* add-on has been employed, which uses a photonic crystal fiber-based supercontinuum laser as probe light source. In contrast to the fs measurements, the pump-probe time delay is controlled electronically with a time resolution of < 1 ns. The sample preparation and data analysis were carried out as described for the fs-transient absorption experiments.

1.3 Syntheses

1.3.1 Synthesis of [Ir(TPP)Cl(CO)]

$[IrCl(COD)]_2$ (164 mg, 0.244 mmol, 1.50 eq) and H_2(TPP) (100 mg, 0.163 mmol, 1.00 eq) are dissolved in 1,2,4-trichlorobenzene (50 mL) in a round-bottom flask attached with a reflux condenser and refluxed for 72 h at 220 °C. The crude product is purified by silica column chromatography, initially with hexane and then a hexane/dcm (1:4) solvent mixture. The fast-moving brown fraction is discarded, and the following red fraction is collected. The red fraction is washed with hexane and the product is obtained as red solid (69 mg, 0.79 mmol, 49 %), which is of sufficient purity for further use.

^{1}**H NMR** (300MHz, CD_2Cl_2): δ (ppm) = 8.95 (s, 8H, pyrrol-H), 8.24 (m, 8H, meta-H), 7.79 (m, 12H, ortho, para-H).

[Ir(tpp)Cl(CO)]

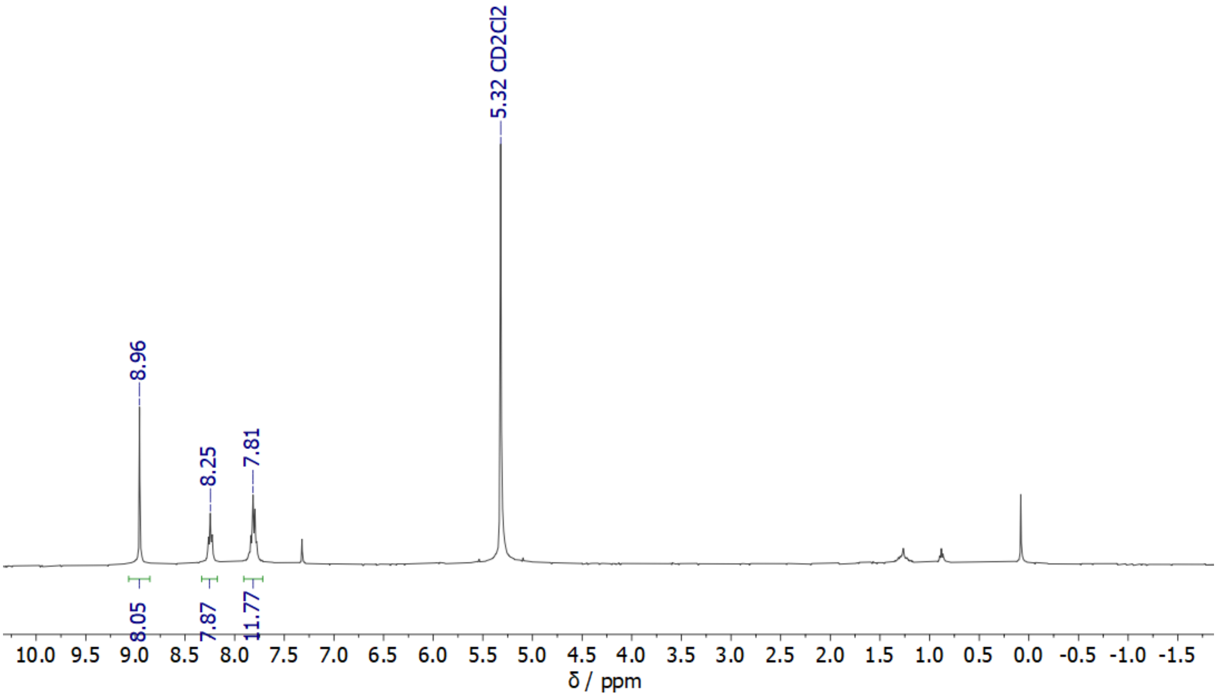

Figure S1: ^{1}H NMR spectrum of [Ir(TPP)Cl(CO)] in DCM-d_2 at room temperature.

1.3.2 Synthesis of [Ir(TPP)H] 8

[IrCl(TPP)(CO)] (68.9 mg, 79.4 µmol, 1.00 eq) is suspended in THF (50mL) in a round-bottom flask equipped with a reflux condenser. A degassed solution of NaOH (2.06 mL, 1.73 M, 3.57 mmol, 45.0 eq) is added together with a degassed solution of $NaBH_4$ (45.0 mg, 1.19 mmol, 15.0 eq). The reaction mixture is refluxed for 3 h to 70 °C. The reaction mixture is cooled with an ice-bath and a solution of HCl (150 mL, 0.84 M) is added. The orange precipitate is filtered and washed with water (3 x 15mL). The crude product is extracted with benzene until the benzene solution becomes colorless. The solvent is removed in vacuo and the crude product is obtained as red solid (62 mg, 0.077 mmol, 98 %).

The crude product contains small amounts of the methyl complex [IrMe(TPP)]. Further purification for photochemical use is best carried out *via* reaction with 2,2,6,6-tetramethylpiperidine-1-oxyl (TEMPO) (see section 1.3.3). The resulting $[Ir(TPP)]_2$ (**9**; 15 mg, 9.3 µmol) is then dissolved in toluene (0.80mL). The solution is degassed twice, and the reaction vessel backfilled with dihydrogen. After stirring for 24 hours stirring at 50 °C, **8** is obtained quantitatively in analytical purity.

The isotopolgue [Ir(TPP)D] (**8^D**) is synthesized from $[Ir(tpp)]_2$ (10mg, 12.4µmol) in toluene (0.60mL) upon reaction with D_2. After 24 hours stirring at 50 °C, **8^D** is obtained quantitatively in analytical purity.

^{1}H NMR (400 MHz, C_6D_6): δ (ppm) = 8.69 (s, 8H, Pyrrol), 8.22 (d, $^3J_{HH}$ = 6.1 Hz, 4H, *ortho*-Ph), 7.99 (d, $^3J_{HH}$ = 6.8 Hz, 4H, *ortho*-Ph), 7.41 (m, 12H, *m,p*-Ph), –57.31 (s, 1H, Ir–*H*).

^{1}H NMR (500 MHz, toluene-d_8): δ (ppm) = 8.61 (s, 8H, Pyrrol), 8.25 (dt, $^3J_{H-H}$ = 7.5 Hz, $^4J_{H-H}$ =1.7 Hz, 4H, *ortho*-Ph), 7.95 (dt, $^3J_{H-H}$ = 7.5 Hz, $^4J_{H-H}$ =1.6 Hz, *ortho*-Ph), 7.54 (td, $^3J_{H-H}$ = 7.5 Hz, $^4J_{H-H}$ =1.6 Hz, 4H, *meta*-Ph), 7.49 (tt, $^3J_{H-H}$ = 7.5 Hz, $^4J_{H-H}$ =1.4 Hz, 4H, *para*-Ph), 7.43 (td, $^3J_{H-H}$ = 7.6 Hz, $^4J_{H-H}$ =1.7 Hz, 4H, *meta*-Ph), –58.00 (s, 1H, Ir–H).

^{13}C{^{1}H} NMR (C_6D_6, 100.6 MHz): δ (ppm) = 143.90 (s, α-Pyrrol), 142.44 (s, *i-Ph*), 134.20 (s, *o-Ph*), 134.03 (s, *o-Ph*), 131.78 (s, β-Pyrrol), 128.35 (s, *m-Ph*,) 127.16 (s, *p-Ph*), 126.96 (s, *m-Ph*), 124.29 (s, *meso-C*)

^{13}C{^{1}H} NMR (126 MHz, toluene-d_8): δ (ppm) = 143.84 (s, α-Pyrrol), 142.48 (s, *i*-Ph), 134.21(s, *o*-Ph), 133.96 (s, *o*-Ph), 131.68 (s, β-Pyrrol), 127.6 (*p*-Ph superimposed with solvent), 127.06 (s, *m*-Ph), 126.90 (s, *m*-Ph), 124.15 (s, *meso-C*).

DOSY NMR: D ($cm^2 s^{-1}$) = $5.50 \cdot 10^{-6}$.

MS (LIFDI, Toluene) m/z found (calc): [$C_{44}H_{29}IrN_4$]: 806.1 (806.2)

EA: found (calculated): [$C_{44}H_{29}IrN_4$]: C 65.50 (65.57), H 3.51 (3.63), N 6.67 (6.95)

IR (ATR, cm^{-1}): ν (cm^{-1}) = 2255 (Ir–H, **8**); 1811 (Ir–D, **8^D**).

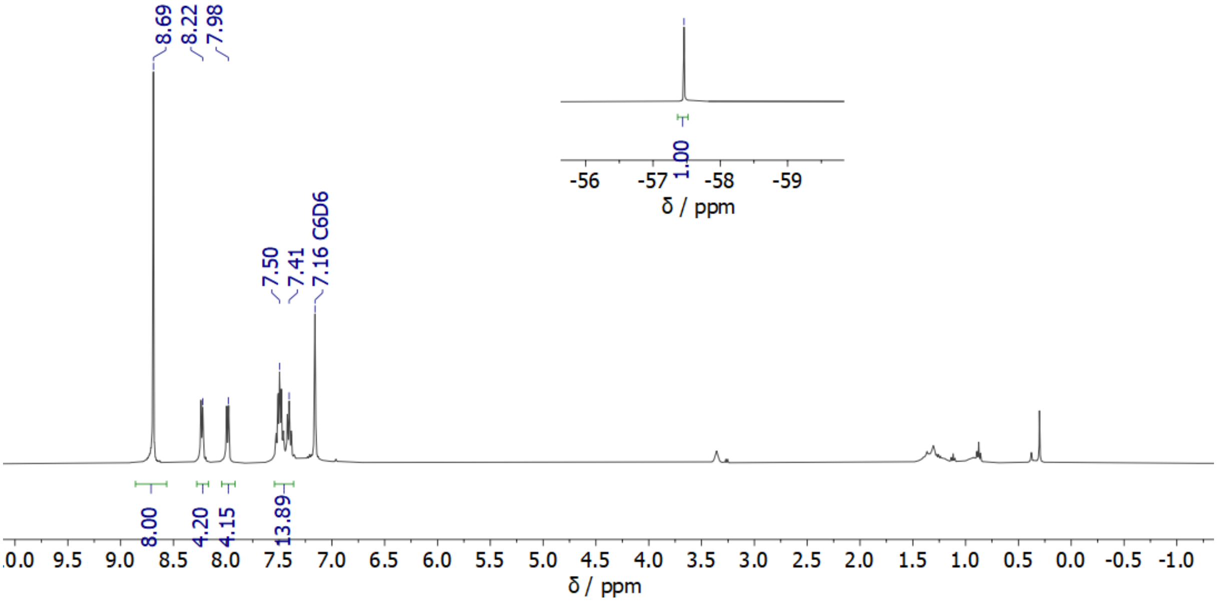

*Figure S2: ¹H NMR spectrum of **8** in C_6D_6 at room temperature.*

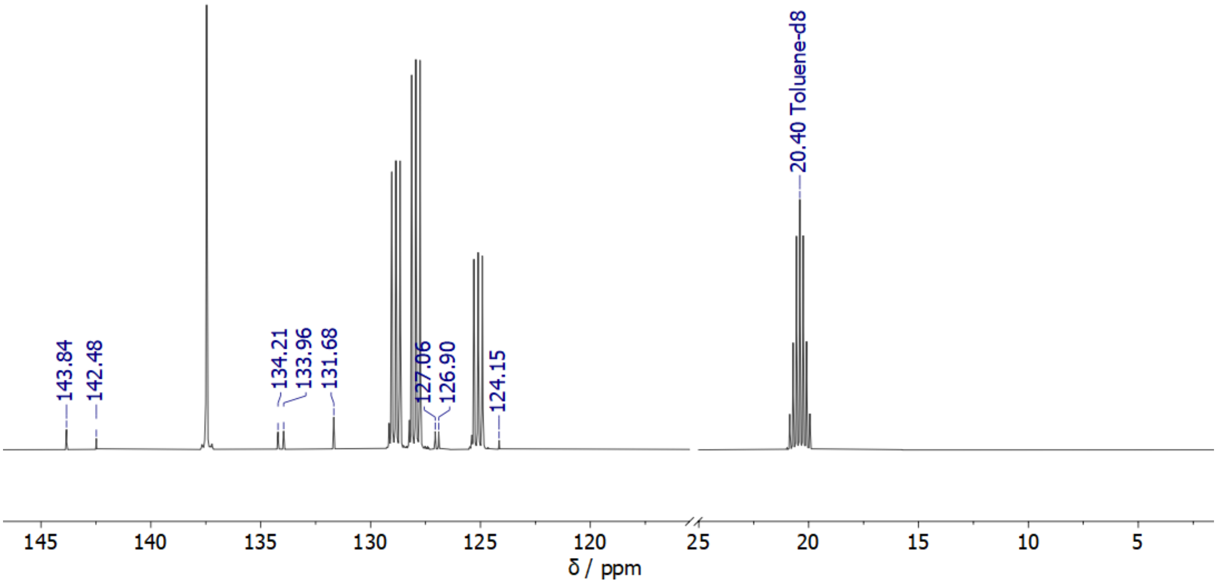

*Figure S3: $^{13}C\{^{1}H\}$ NMR of **8** in toluene-d_8 at room temperature.*

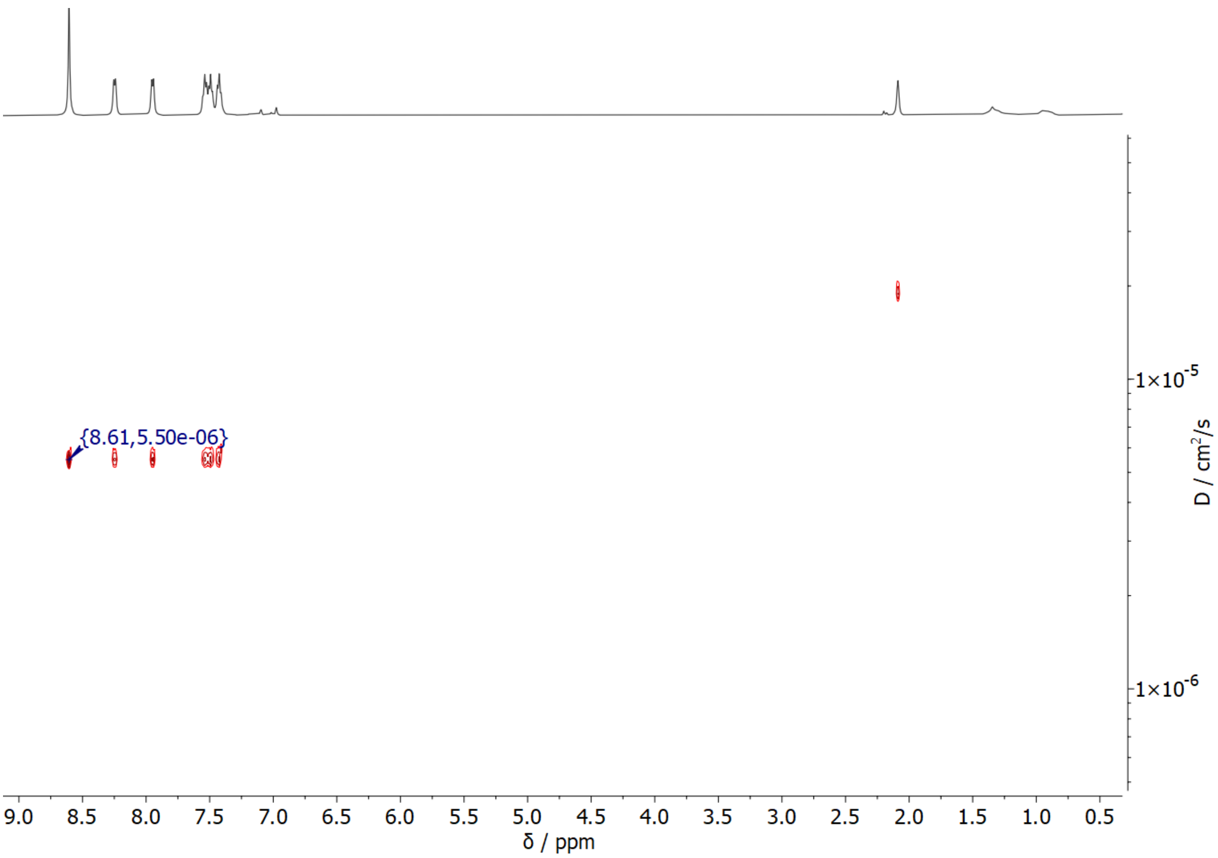

*Figure S4: DOSY NMR spectrum of **8** in toluene-d_8 at room temperature.*

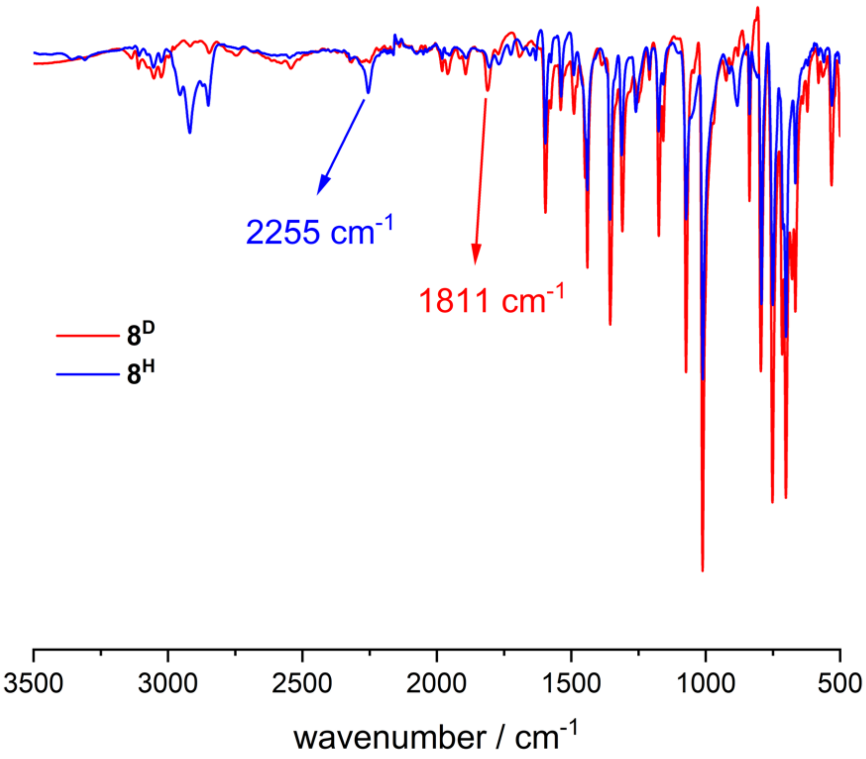

*Figure S5: ATR-IR spectrum of **8**.*

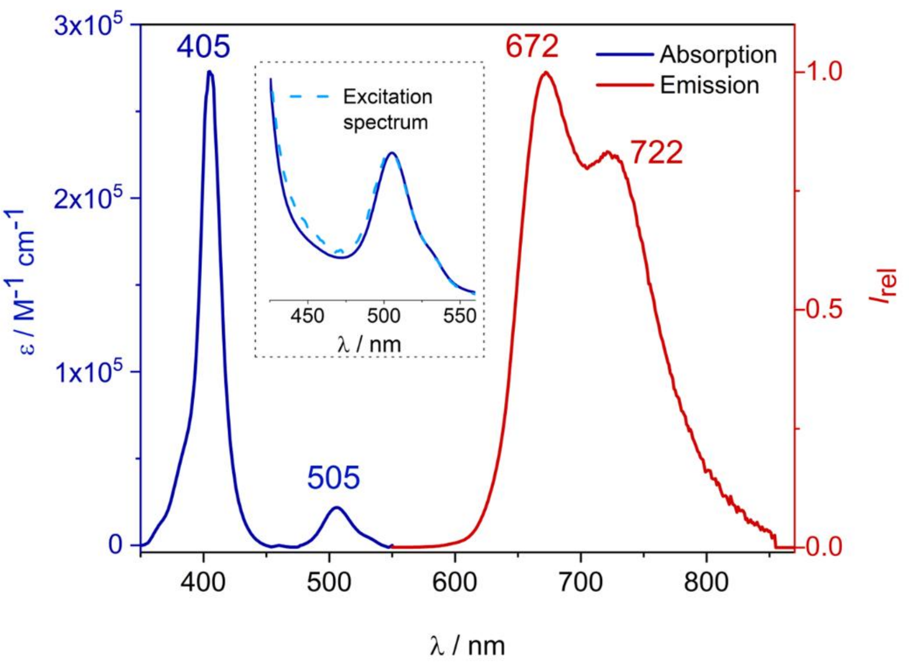

*Figure S6: UV-vis Absorption (blue) and emission (red, λ_{exc} = 505 nm) spectra of **8** in 1,2-difluorobenzene at room temperature with a comparison of the absorption and excitation (dashed, λ_{em} = 670 nm) spectra in the Q-band region as inset.*

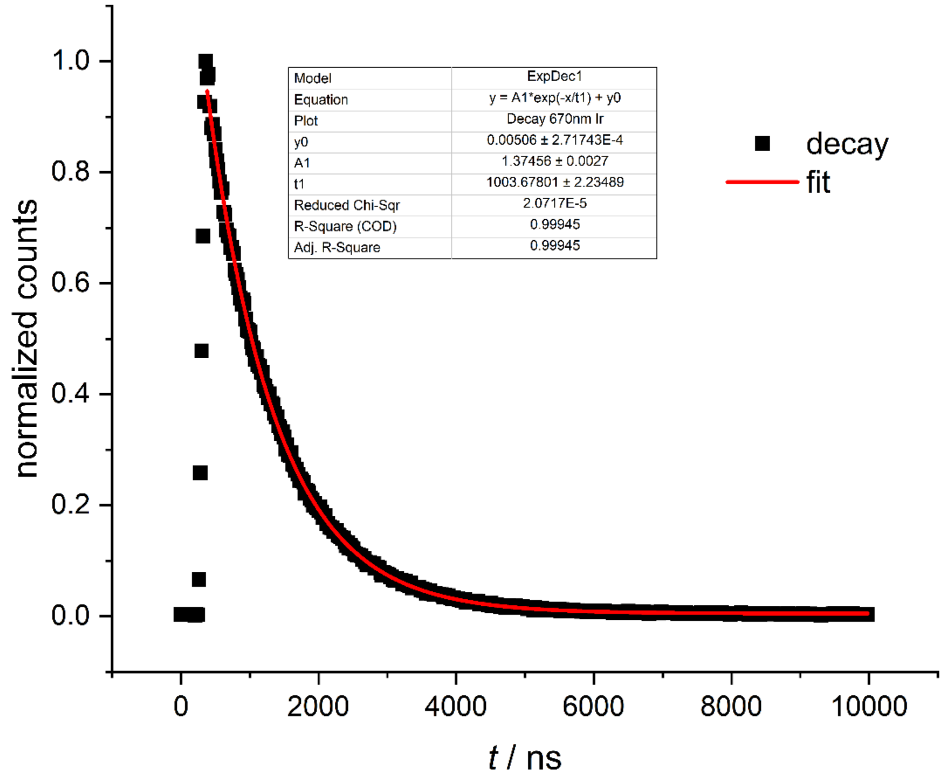

*Figure S7: Luminescence decay of **8** in 1,2-difluorobenzene at room temperature from time correlated single photon counting (black squares; λ_{exc} = 450 nm, λ_{em} = 670 nm) and single exponential fit (τ_{obs} = 1.0 µs, red).*

1.3.3 Synthesis of $[Ir(TPP)]_2$ (9)

8 (25 mg, 31 µmol, 1.0 eq) is dissolved in benzene (2mL) and 2,2,6,6-tetramethylpiperidine-1-oxyl (TEMPO) (6.8 mg, 43 µmol, 1.4 eq) is added. The reaction mixture is stirred for 30 minutes. The product precipitates as brown solid. The reaction mixture is filtered, and the precipitate is washed with pentane (3 x 4mL) and extracted with toluene until the solution becomes colorless. The solvent is removed *in vacuo* to obtain the product as brown solid (15 mg, 60 %)

^{1}H NMR (500 MHz, toluene-d_8): δ (ppm) = 9.44 (dt, $^{3}J_{H\text{-}H}$ = 7.6 Hz, $^{4}J_{H\text{-}H}$ = 1.7 Hz, 4H, *ortho*-Ph-*H*), 8.12 (s, 8H, Pyrrol-*H*), 7.84 (td, $^{3}J_{H\text{-}H}$ = 7.6 Hz, $^{4}J_{H\text{-}H}$ = 1.3 Hz, 4H, *meta*-Ph-*H*), 7.53 (tt, $^{3}J_{H\text{-}H}$ = 7.7 Hz, $^{4}J_{H\text{-}H}$ = 1.3 Hz, 4H, *para*-Ph-*H*), 7.27 (td, $^{3}J_{H\text{-}H}$ = 7.6 Hz, $^{4}J_{H\text{-}H}$ = 1.3 Hz, 4H, *meta*-Ph-*H*), 7.18 (dt, $^{3}J_{H\text{-}H}$ = 7.6 Hz, $^{4}J_{H\text{-}H}$ = 1.3 Hz, 4H, *ortho*-Ph-*H*).

^{13}C{^{1}H} NMR (126 MHz, toluene-d_8): δ (ppm) = 144.63 (s, α-Pyrrol), 143.37 (s, *i-Ph*), 135.89 (s, *o-Ph*), 134.39 (s, *o-Ph*), 131.56(s, β-Pyrrol), 127.71 (s, *m-Ph*), 127.24 (s, *m-Ph*), 124.53 (s, Pyrrol-*C*-Pyrrol).

DOSY NMR: *D* ($cm^2\,s^{-1}$) = $4.55 \cdot 10^{-6}$.

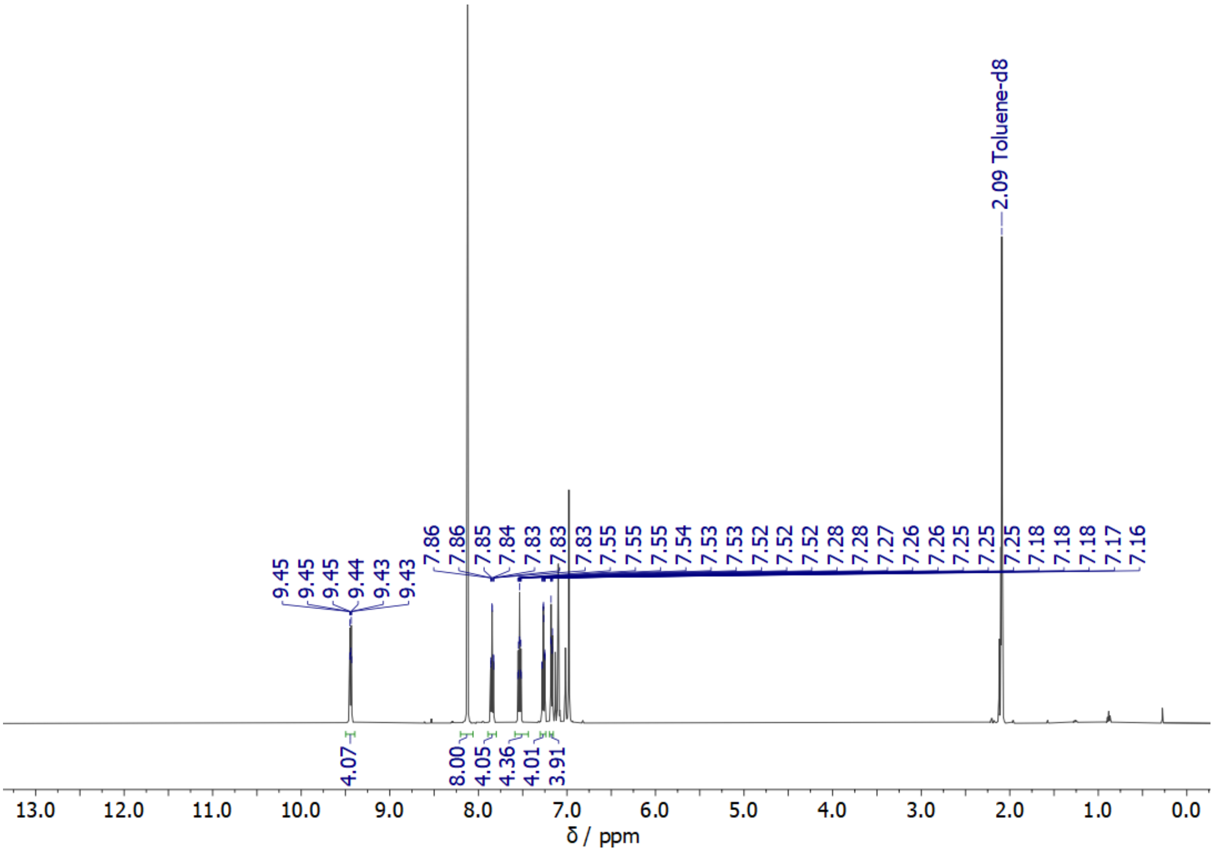

*Figure S8: ^{1}H NMR spectrum of **9** in toluene-d_8 at room temperature.*

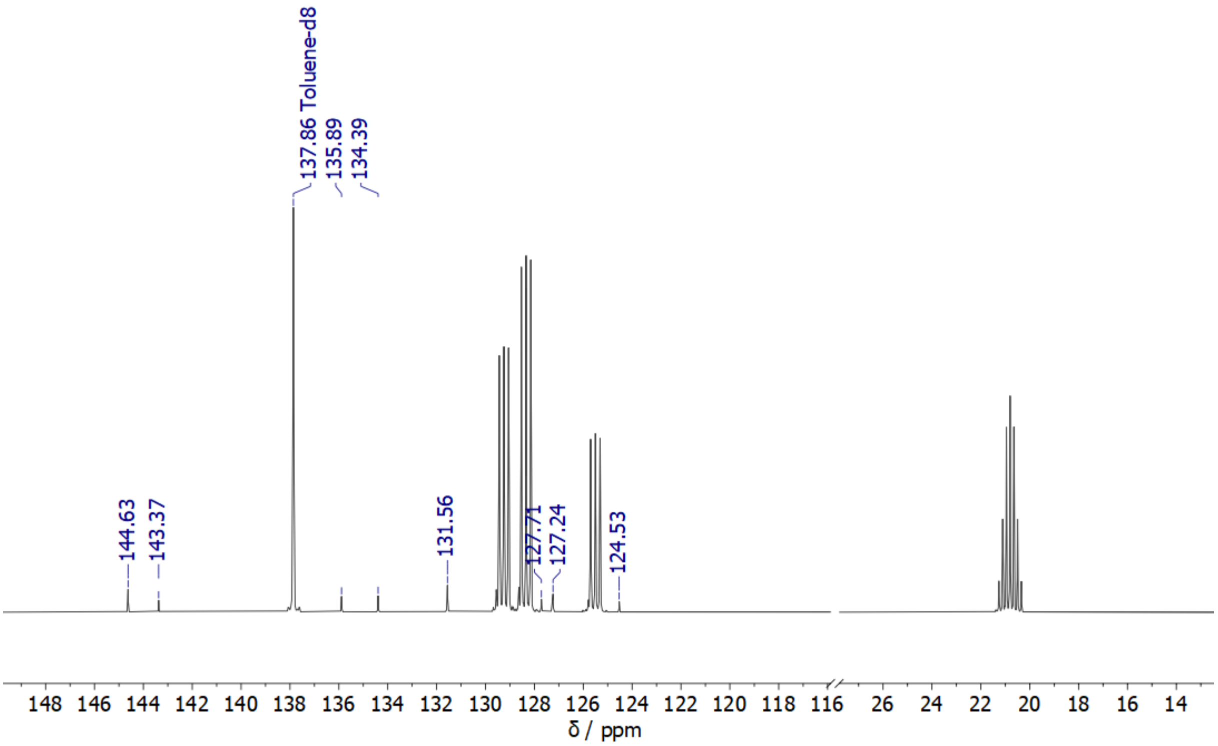

*Figure S9: $^{13}C\{^{1}H\}$ NMR spectrum of **9** in toluene-d_8 at room temperature.*

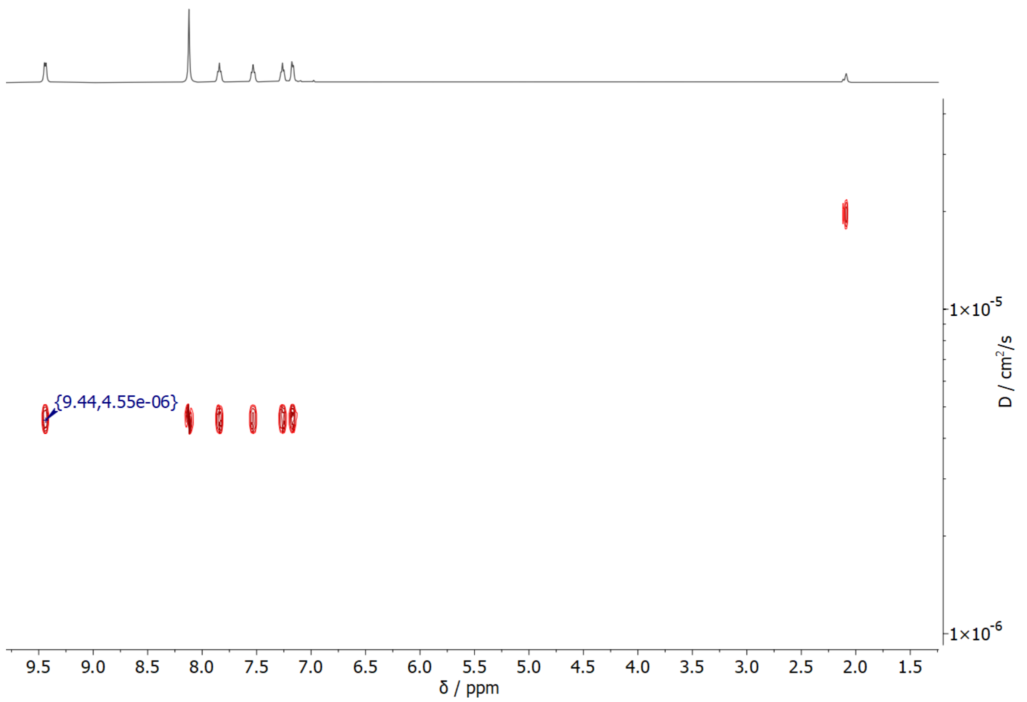

*Figure S10: DOSY NMR spectrum of **9** in toluene-d_8 at room temperature.*

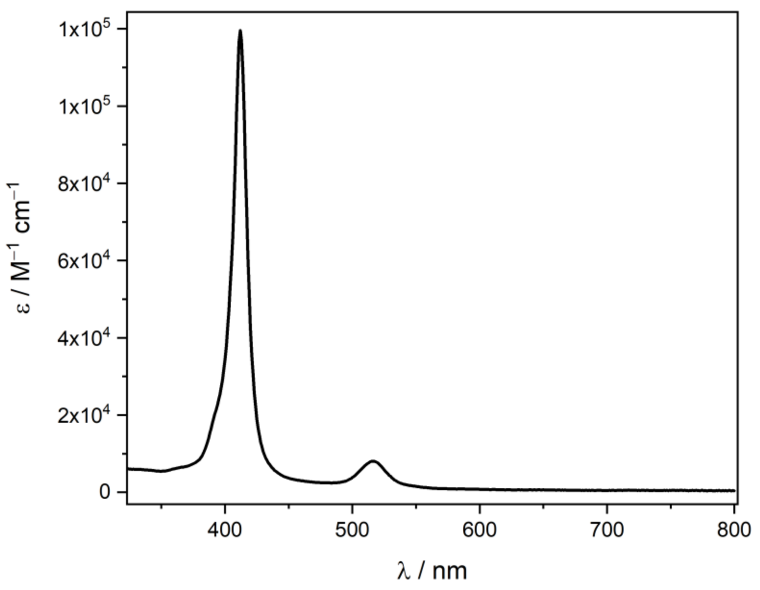

*Figure S11: UV-vis Absorption spectrum of **9** in toluene at room temperature*

1.3.4 Photolysis of [Ir(TPP)H] (8)

Complex **8** (2.0 mg, 2.5 µmol) is dissolved in C_6D_6 (0.5 mL) and photolyzed with an LED (λ = 456 nm or λ = 525 nm). ^1H NMR spectroscopic examination evidenced the formation of **9** and H_2 as photoproducts.

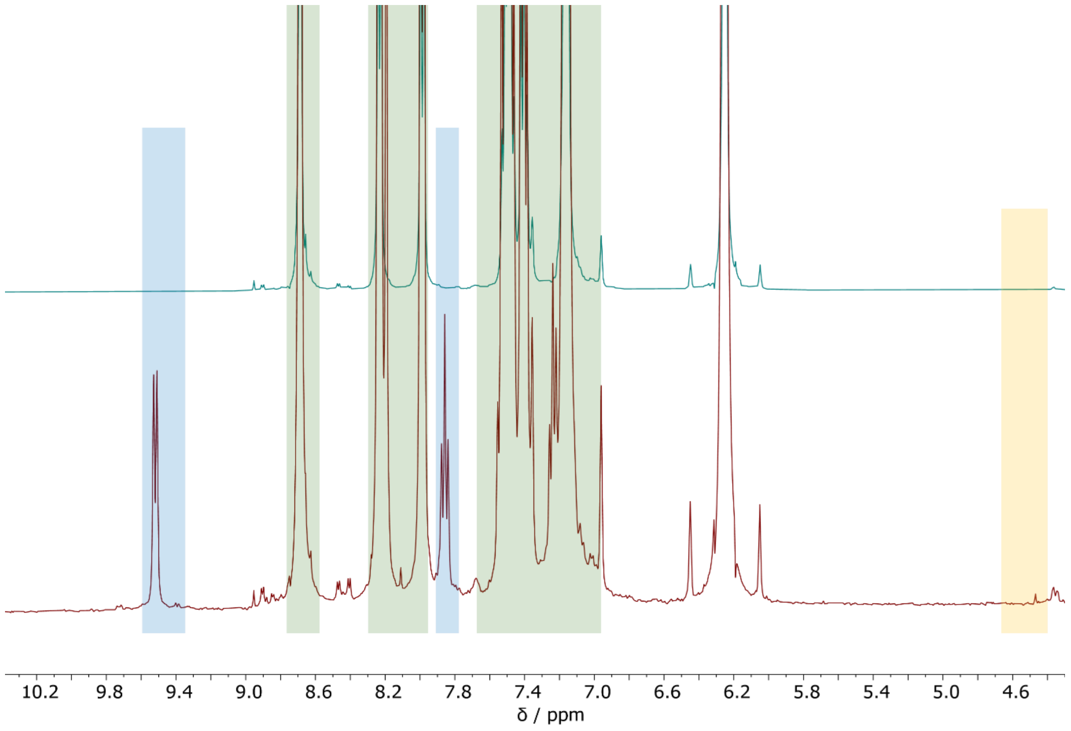

*Figure S12: Top: ^1H NMR spectrum of **8** in C_6D_6. Bottom: NMR spectrum after photolysis (λ = 456 nm) for 2 h (blue: **9**, green: **8**, yellow: H_2).*

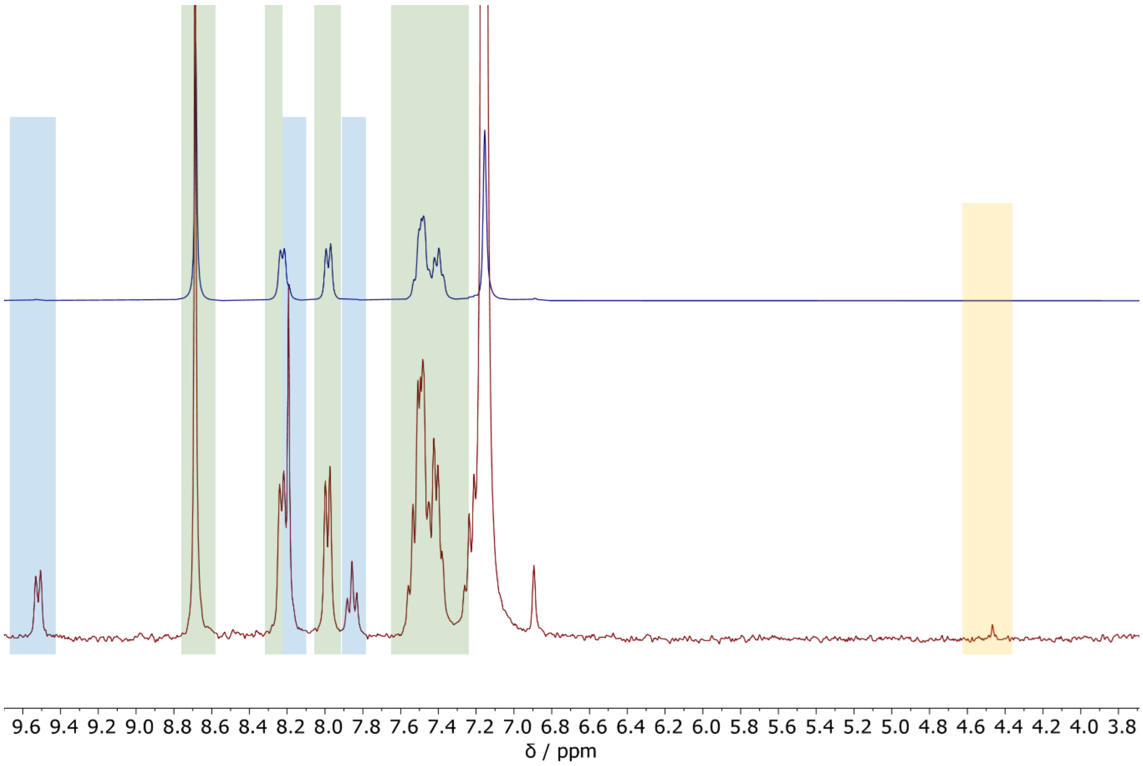

*Figure S13: Top: ^{1}H NMR spectrum of **8** in C_6D_6. Bottom: NMR spectrum after photolysis (λ = 525 nm) for 24 h (blue: **9**, green: **8**, yellow: H_2).*

1.3.5 Synthesis of $[ReBr_2(HPNP)(NCPh)]^+$ (**6**$^+$)

Route 1: $[ReBr_3(HPNP)]$ (**1**, 6.0 mg, 8.2µmol), benzonitrile (4.2µL, 41µmol, 5.0 eq) and $TlPF_6$ (3.0 mg, 8.6µmol, 1.05 eq) are dissolved in DCM and the solution is stirred at room temperature for 16 hours. The solution is filtered, and the solvent is removed *in vacuo*. The solid is washed with pentane and redissolved in THF and filtered. The product **6**(PF_6) is obtained as yellow solid (7.2mg, 6.4µmol, 78%). The spectroscopic properties of the cation are identical with those of **6**$^+$ obtained from route 2.

Route 2: $[Re(NC(O)Ph)Br(HPNP)][BAr^F_{24}]$ (**3**(BAr^F_{24}), 6.0 mg, 3.7 µmol) is dissolved in THF-d_8 (0.5 mL) and photolyzed for 24 h with a blue LED (456 nm). After photolysis, the solvent is removed *in vacuo*. The crude reaction product is washed with pentane (3x3mL) and extracted with ether until the solution becomes colorless. The solvent is removed *in vacuo* and **6**(BAr^F_{24}) is obtained as yellow solid (4.9 mg, 3.0 µmol, 82%). Crystals suitable for X-Ray diffraction were obtained by layering a diethyl ether solution with pentane –80 °C.

Elem. Anal. found (calc) for $C_{55}H_{54}BBr_2F_{24}ReN_2P_2$: C 40.89 (40.83); H 3.64 (3.36); N 1.57 (1.73).

^{1}**H NMR** (500MHz, THF-d_8): *δ* (ppm) = 298.53 (s, 1H, NH), 17.57 (m, 2H, N-CH_2-CH**H**), 13.63 (t, $^{3}J_{HH}$=7.6Hz, 2H, C_{ortho}**H** (NCPh)), 13.54 (m, 2H, N-C**H**H-CH_2), 11.95 (d, 6H, $^{3}J_{HH}$=6.5Hz, CH(C**H**$_3$)$_2$), 11.00 (d, $^{3}J_{HH}$=6.5Hz, 6H, CH(C**H**$_3$)$_2$), 10.52 (m, 12H, overlapping 2x CH(C**H**$_3$)$_2$), 7.82 (s, 8H, BAr^F_{24}), 7.56 (s, 4H, BAr^F_{24}), 3.81 (d, 2H, $^{3}J_{HH}$=7.2Hz, C_{meta}**H** (NCPh)), 3.48 (m, 2H, NC**H**H-CH_2), 2.87 (m, 2H, NCH_2-C**H**H), 2.34 (m, 2H, very broad, C**H**Me_2), 1.64 (t, 1H, $^{3}J_{HH}$=7.7Hz, C_{para}**H** (NCPh)), −1.70 (m, 2H, C**H**Me_2).

^{31}**P{^{1}H} NMR** (202 MHz, THF-d_8): *δ* (ppm) = −2526.

^{13}**C{^{1}H} NMR** (125.8MHz, THF-d_8): *δ* (ppm) = 227.2 (s, **C**$_{meta}$ (NCPh)), 168.1 (s, **C**$_{para}$ (NCPh)), 163.0 (q, $^{1}J_{C-11B}$ = 50Hz, C_{ipso} (BAr^F_{24}) + (hept, $^{1}J_{C-10B}$=17Hz, **C**$_{ipso}$ (BAr^F_{24})), 135.8 (s, **C**$_{ortho}$ (BAr^F_{24}), 130.2 (qq, $^{2}J_{C-F}$=32 Hz, $^{4}J_{C-F}$=2.7Hz, **C**$_{meta}$ (BAr^F_{24}), 125.7 (q, $^{1}J_{C-F}$=272Hz, **C**F_3), 122.4 (s, broad, **C**HMe_2), 118.4 (hept, $^{3}J_{C-F}$=4Hz, **C**$_{para}$ (BAr^F_{24})), 111.5 (d, $^{5}J_{CP}$=19.5 Hz, **C**$_{ortho}$(NCPh)), 111.1 (d, $^{2}J_{CP}$=13.0 Hz, N-**C**H_2-CH_2), 90.4 (s, broad,

CHMe₂), 32.4 (s, broad, **C**H(CH₃)₂), 28.1 (s, broad, CH-(**C**H₃)₂), −2.9 (s, CH(**C**H₃)₂), −4.3 (s, CH(**C**H₃)₂), −14 (d, $^{1}J_{CP}$=39.7 Hz, N-CH₂-**C**H₂), −23.5 (s, **C**$_{ipso}$ (NCPh)). **C**N-Ph not found

MS (LIFDI, THF) m/z found (calc)[$C_{23}H_{42}Br_2N_2P_2Re$]$^+$: 755.0 (755.1).

IR (THF, cm^{-1}): *ν* (cm^{-1}) = 2207 (C≡^{14}N), 2182 (C≡^{15}N).

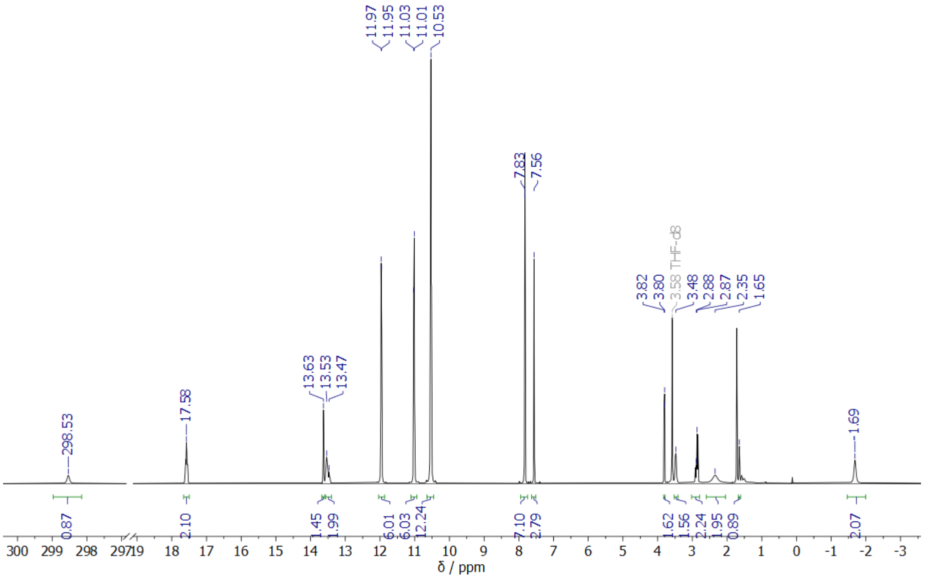

*Figure S14: ¹H NMR spectrum of **6**(BAr^F$_{24}$) in THF-d$_8$ at room temperature.*

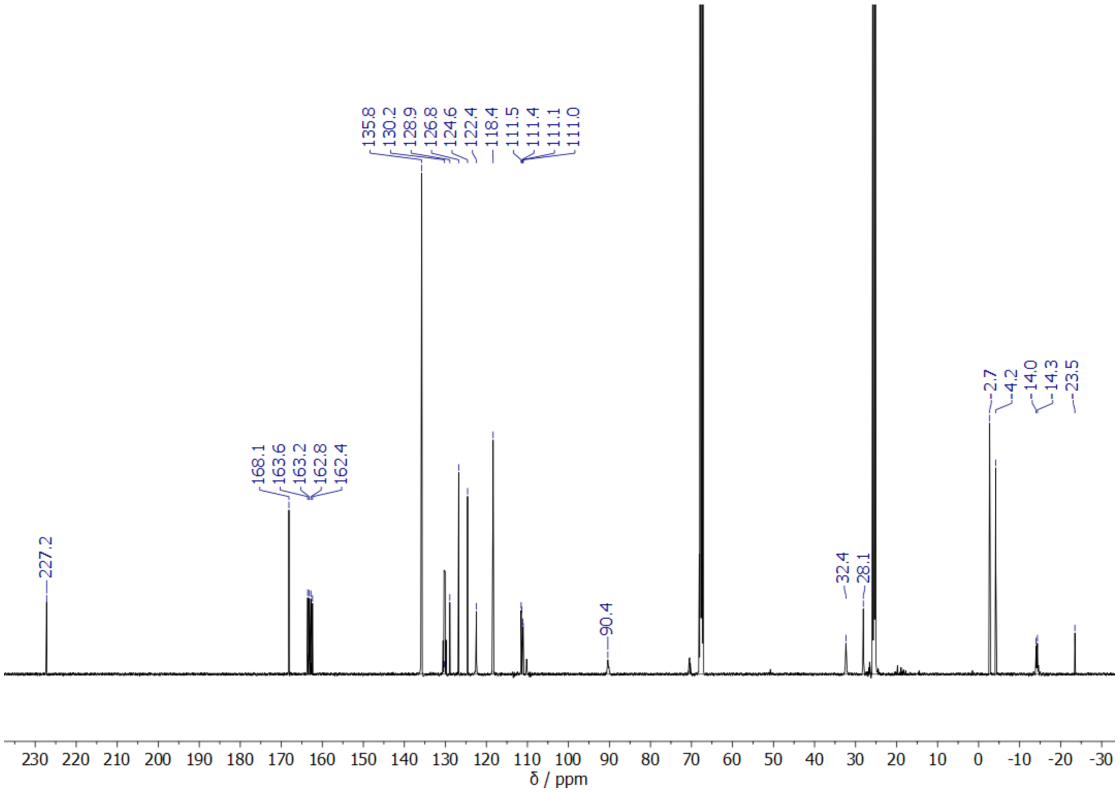

*Figure S15: $^{13}C\{^{1}H\}$ NMR spectrum of **6**(BAr^F$_{24}$) in THF-d$_8$ at room temperature.*

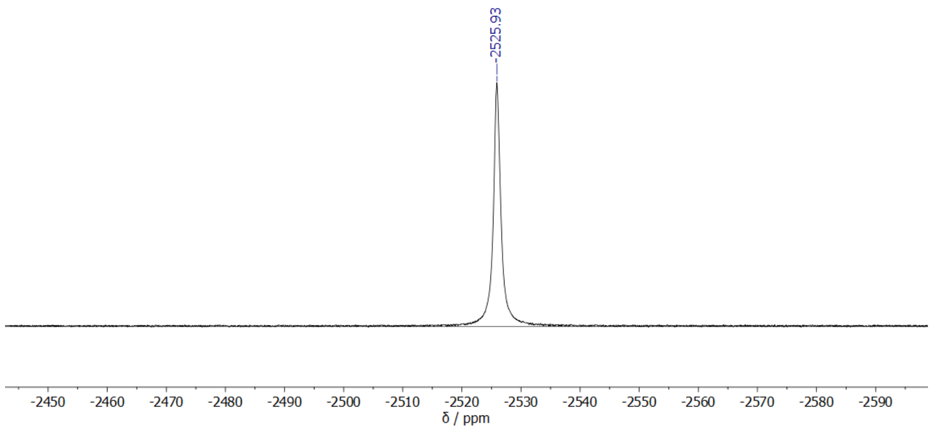

*Figure S16: $^{31}P\{^{1}H\}$ NMR spectrum of **6**(BAr^{F}_{24}) in THF-d_8 at room temperature.*

1.3.6 Synthesis of $[ReBr_2\{OC(NH_2)Ph\}(HPNP)]^+$ (*trans*-**5** (BAr^{F}_{24}))

$[ReBr_3(HPNP)]$ (**1**; 7.0 mg, 9.6 µmol, 1.0 eq), benzamide (4.6 mg, 38 µmol, 4.0 eq) and $TlPF_6$ (3.7 mg, 10 µmol, 1.1 eq) are dissolved in DCM and the solution is stirred at room temperature for 12 hours. The solvent is removed, and the residual solid is washed with toluene until the solution is almost colorless. The residual solid is extracted with THF, filtered, and the solvent is removed *i. vac*. The crude product is suspended in diethylether (4 mL) and $NaBAr^F$ (4.3 mg, 4.8 µmol, 0.70 eq) is added. The reaction mixture is stirred for 16 hours at room temperature. The yellow solution is filtered and the solvent removed *in vacuo*. *trans*-**5**(BAr^{F}_{24}) is obtained as yellow, solid BAr^{F}_{24}-salt (15mg, 8.7µmol, 45%). Crystals suitable for X-Ray diffraction are obtained by layering a diethyl ether solution with pentane at –80 °C.

^{1}H NMR (500MHz, THF-d_8): δ (ppm) = 223.77 (s, 1H, $PN^{H}P$), 11.82 (m, 2H, N-CH_2-CH**H**), 10.72 (s, 1H, N**H**), 10.24 (d, $^{3}J_{HH}$=7.6Hz, 6H, CH(**CH$_3$**)$_2$), 9.93 (d, $^{3}J_{HH}$=7.6Hz, 6H, CH(**CH$_3$**)$_2$), 9.30 (d, $^{3}J_{HH}$=7.6Hz, 6H, CH(**CH$_3$**)$_2$), 9.07 (d, $^{3}J_{HH}$=7.6Hz, 6H, CH(**CH$_3$**)$_2$), 7.80 (s, 8H, BAr^{F}_{24}), 7.56 (m, 4H, BAr^{F}_{24}), 7.49 (s, 1H, C_{para}**H** ($NH_2C(O)$**Ph**)), 7.39 (m, 2H, C_{meta}**H** ($NH_2C(O)$**Ph**)), 6.69 (d, $^{3}J_{HH}$=7.6Hz, 2H, C_{ortho}**H** ($NH_2C(O)$**Ph**)), 6.46 (m, 2H, very broad, C**H**Me_2), 4.08 (m, 2H, very broad, C**H**Me_2), 3.51 (m, 2H, N-CH_2-CH**H**), 2.69 (m, 2H, P-CH_2-CH**H**), –4.88 (m, 2H, P-CH_2-CH**H**), -13.86 (s, 1H, N**H**).

$^{13}C\{^{1}H\}$ NMR (125.8MHz, THF-d_8): δ (ppm) = 189.0 (**C**$_{ipso}$($NH_2C(O)$**Ph**))*, 163.0 (m, **C**$_{ipso}$ (BAr^{F}_{24}), 149.3 (s, broad, N-**C**H_2-$CH_{2)}$, 144.0 (s, **C**$_{ortho}$ ($NH_2C(O)$**Ph**)), 138.8 (s, **C**$_{para}$ ($NH_2C(O)$**Ph**)), 135.8 (s, **C**$_{ortho}$ (BAr^{F}_{24}), 130.2 (m, **C**$_{meta}$ (BAr^{F}_{24}),129.9 (s, **C**$_{meta}$ ($NH_2C(O)$**Ph**)), 125.7 (q, $^{1}J_{C\text{-}F}$=272Hz, **C**F_3), 118.4 (m, **C**$_{para}$ (BAr^{F}_{24})), 112.2 (s, **C**$_{para}$ (NH_2**C**(O)Ph)), 107.4 (s, broad, **C**HMe_2), 106.2 (s, broad, **C**HMe_2), 32.4 (s, P-**C**H_2-CH_2), 30.8 (s, CH(**C**H_3)$_2$), 26.9 (s, CH(**C**H_3)$_2$), 9.3 (s, CH(**C**H_3)$_2$), 9.2 (s, CH(**C**H_3)$_2$).*signal identified by $^{1}H/^{13}C$ HMBC.

$^{31}P\{^{1}H\}$ NMR (202 MHz, THF-d_8): δ (ppm) = −1791.

$^{31}P\{^{1}H\}$ NMR (121 MHz, 1,2-difluorobenzene): δ (ppm) = −1872.

^{15}N NMR (50.71 MHz, THF-d_8): –56.23 (d, $^{1}J_{N\text{–}H}$ = 88 Hz), –58.01 (d, $^{1}J_{N\text{–}H}$ = 88 Hz).

MS (LIFDI, THF) m/z found (calc)$[C_{23}H_{44}Br_2N_2OP_2Re]^+$: 772.9 (773.1).

IR (ATR): ν (cm^{-1}) = 1639 (C≡O)

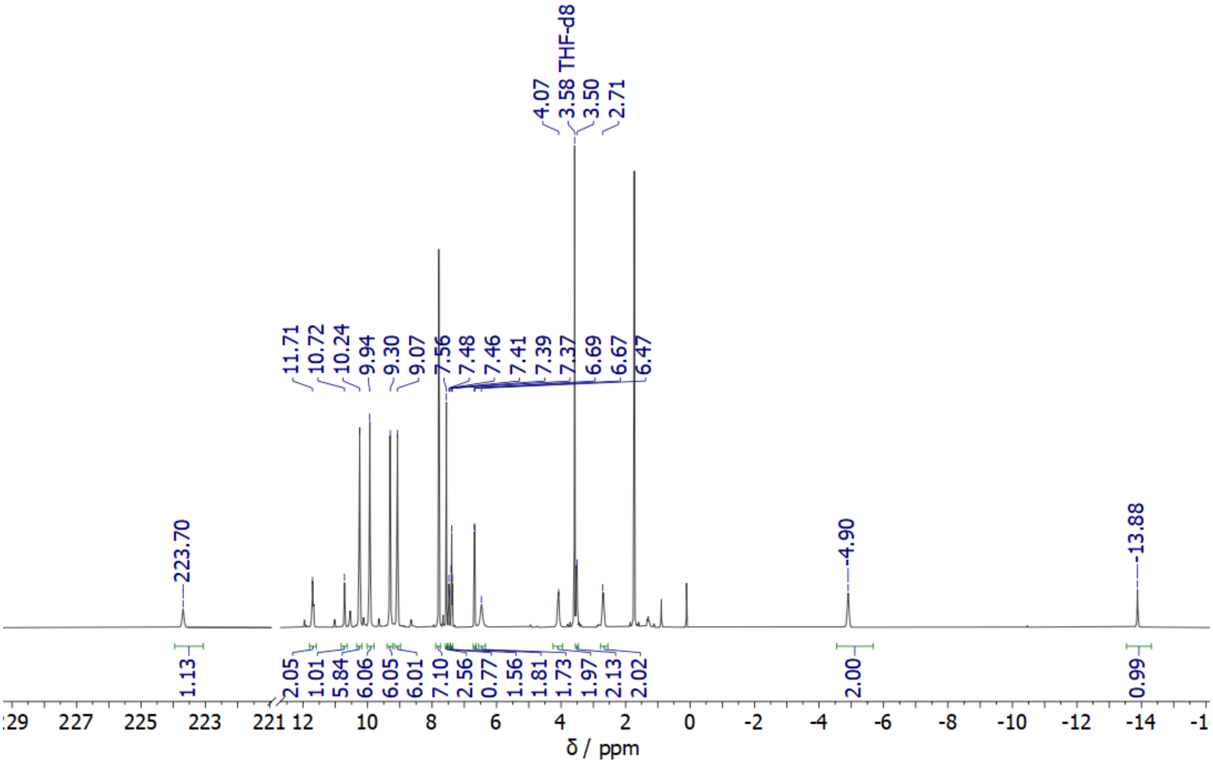

*Figure S17: ^{1}H NMR spectrum of trans-**5**(BAr^F_{24}) in THF-d_8 at room temperature.*

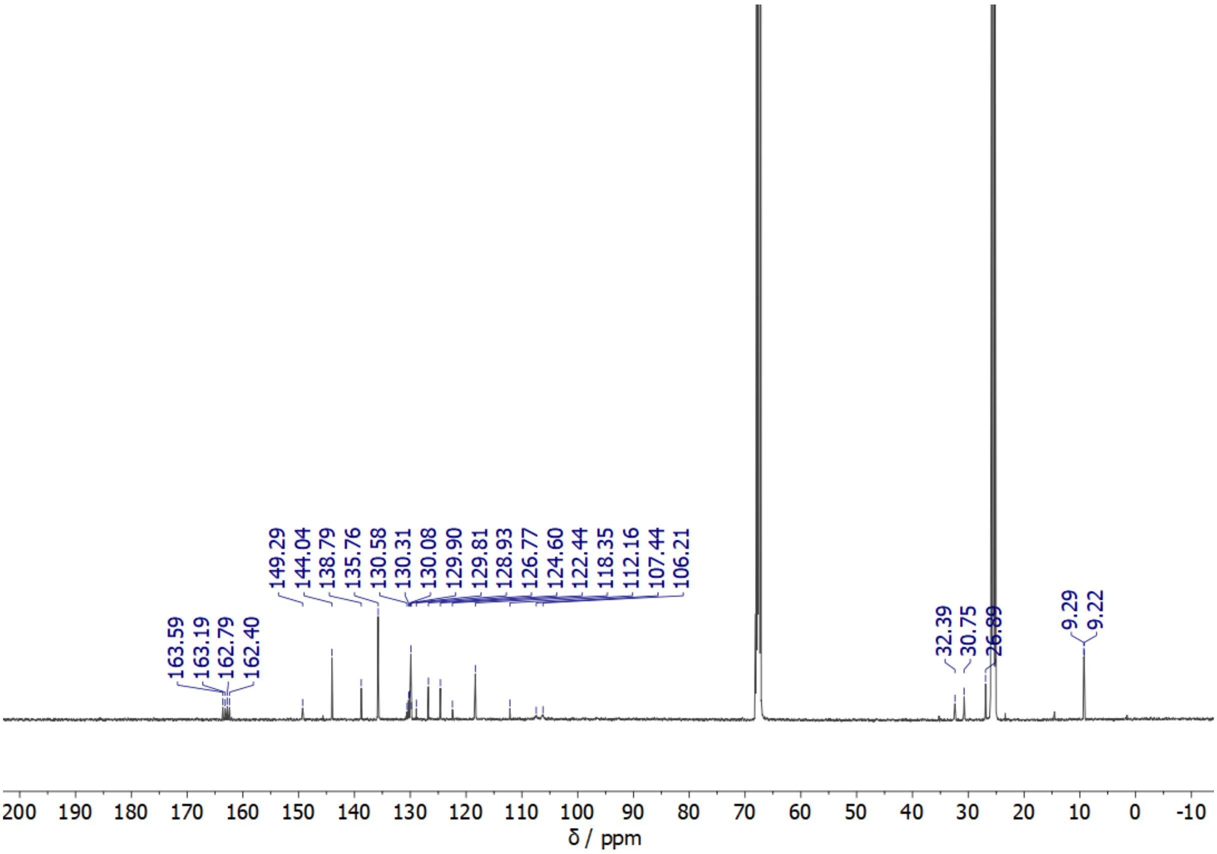

*Figure S18: ^{13}C{^{1}H} NMR spectrum of trans-**5**(BAr^F_{24}) in THF-d_8 at room temperature.*

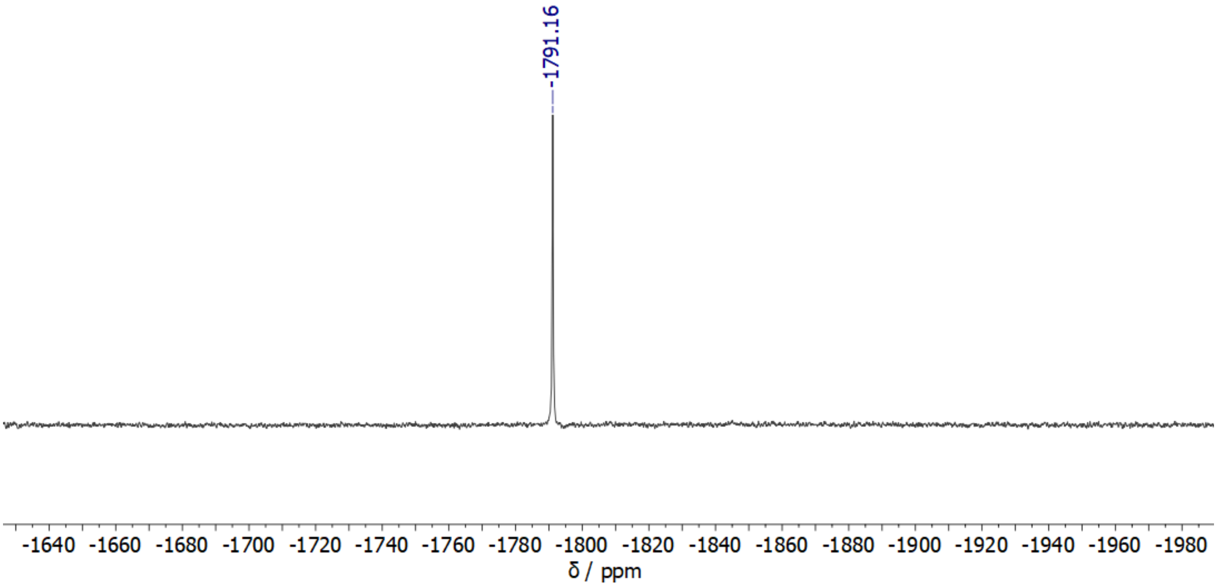

*Figure S19: $^{31}P\{^1H\}$ NMR spectrum of trans-**5**($BAr^F{}_{24}$) in THF-d_8 at room temperature*

1.3.7 UV-vis Spectra of **3** and **4**

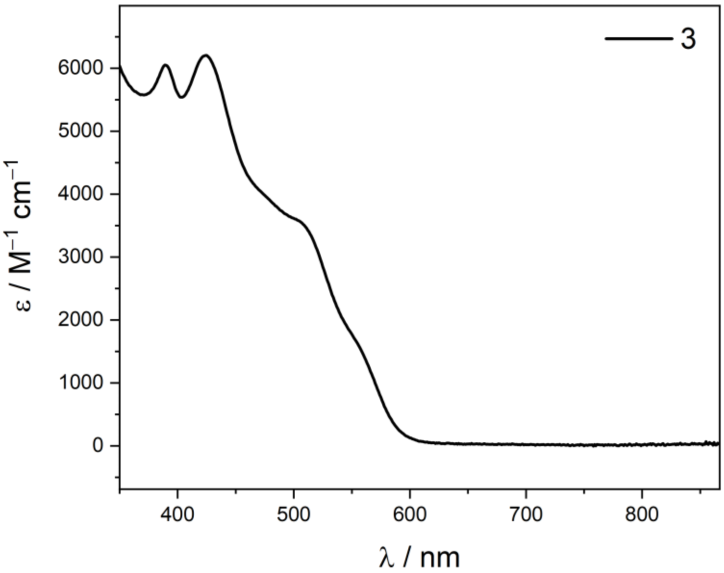

*Figure S20: UV-vis Absorption spectrum of **3** in THF at room temperature.*

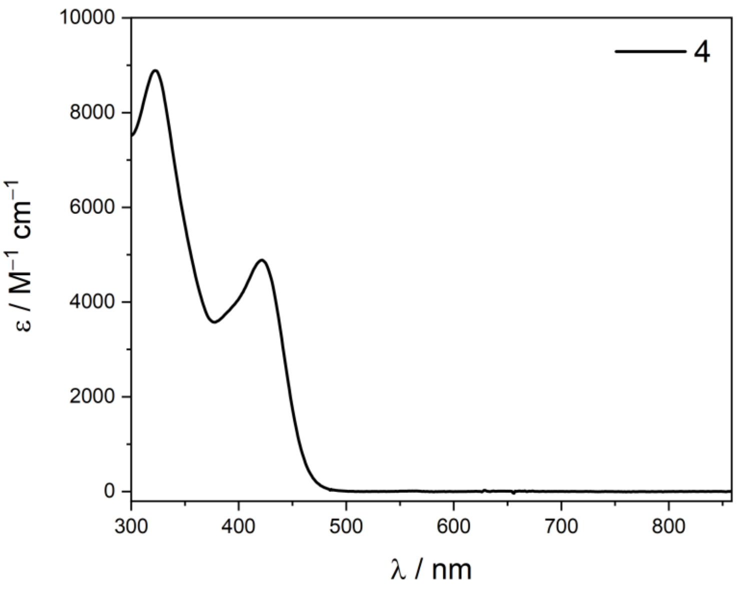

*Figure S21: UV-vis Absorption spectrum of **4** in THF at room temperature.*

1.4 Electrochemical Investigations

1.4.1 Electrochemical examination of **3**(BAr^F_{24}) in 1,2-difluorobenzene

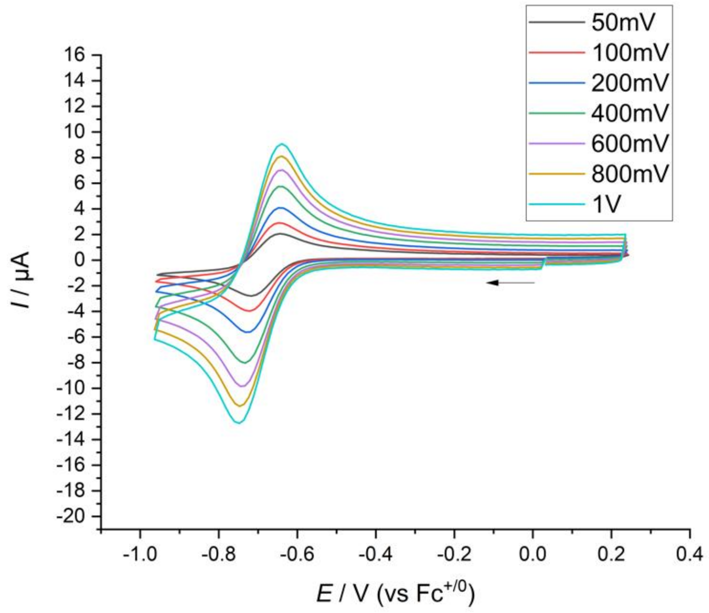

*Figure S22: Scan rate dependent CV of **3**(BAr^F_{24}) (1 mM, electrolyte: 0.1 M [$^nBu_4N$][PF_6] in 1,2-diflurobenzene).*

1.4.2 Electrochemical examination of **8** in 1,2-difluorobenzene

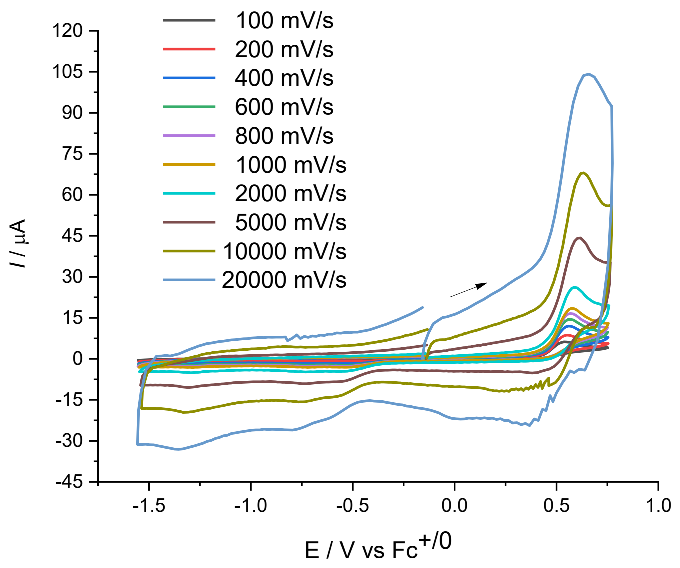

*Figure S23: Scan rate dependent CV of **8** (1 mM, electrolyte: 0.1 M [^{n}Bu$_4$N][PF$_6$]in 1,2-diflurobenzene).*

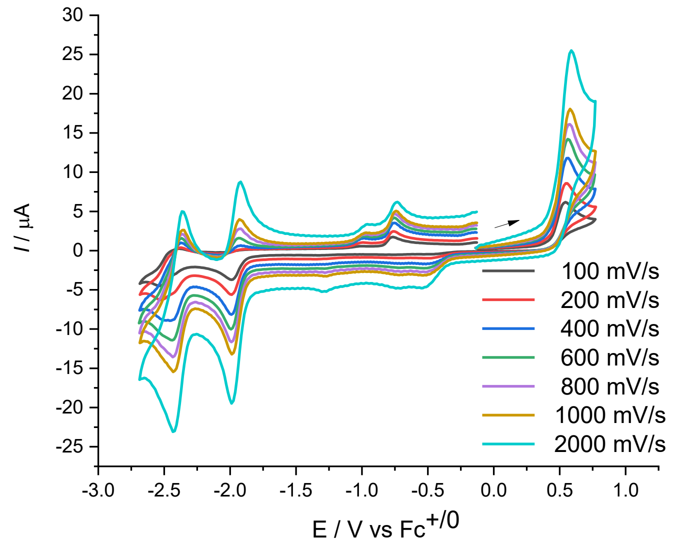

*Figure S24: Scan rate dependent CV of **8** (1 mM, electrolyte: 0.1 M [^{n}Bu$_4$N][PF$_6$]in 1,2-diflurobenzene).*

The number of electrons for each electrochemical event was determined by chronoamperometry using step potentials of E = 0.77 V, –2.2 V, –2.6 V, respectively, with a step time of 30 s, and a sample period of 0.01, 0.02, 0.05 and 0.1 s. The internal resistance is compensated by 90 %. The number of electrons are calculated with the Cottrell equation:

$$i = \frac{nFAc\sqrt{D}}{\sqrt{t\pi}}$$

i: current, *n*: number of electrons, *F*: Faraday constant, *A*: area of the electrode (0.02 cm^2), *c*: concentration (9.9 10^{-7} mol/cm^3), *D*: diffusion constant, *t*: time

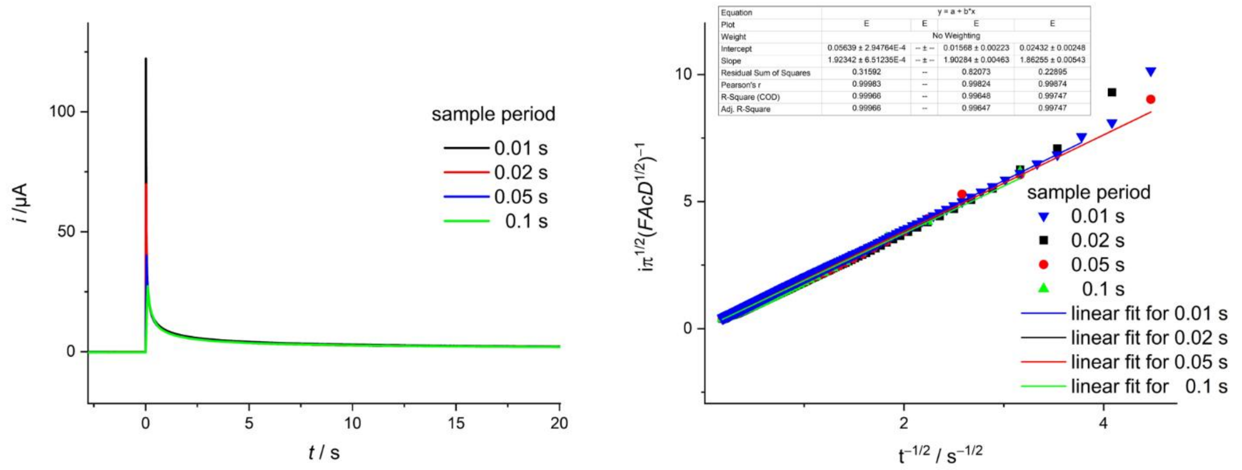

Figure S25: Left: Chronoamperometry at the step potential E = 0.77 V with different sample periods (0.01, 0.02, 0.05, 0.1 s). Right: Cottrell-plots with linear fit for different sample periods (0.01, 0.02, 0.05, 0.1 s).

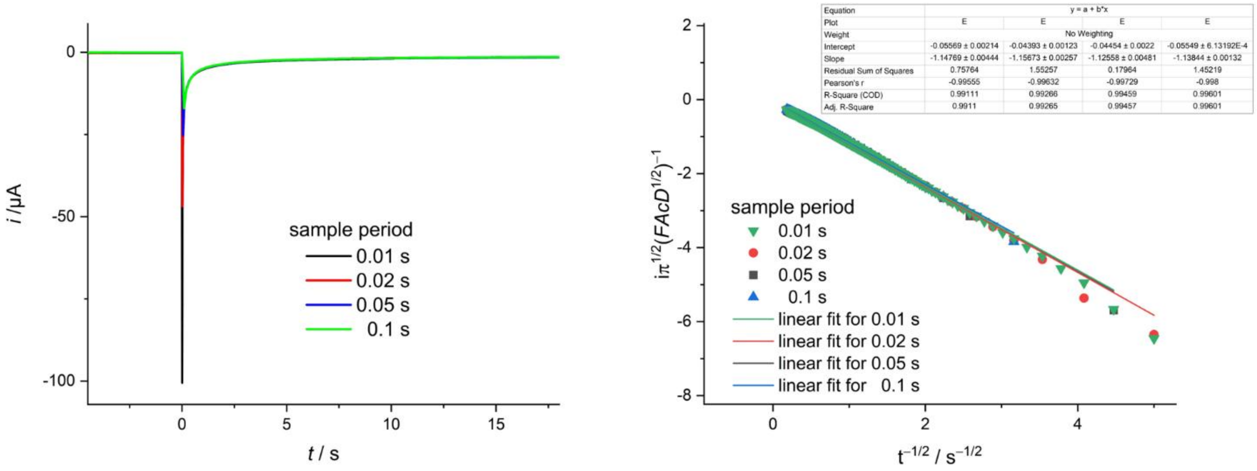

Figure S26: Left: Chronoamperometry at the step potential E = –2.2 V with different sample periods (0.01, 0.02, 0.05, 0.1 s). Right: Cottrell-plots with linear fit for different sample periods (0.01, 0.02, 0.05, 0.1 s).

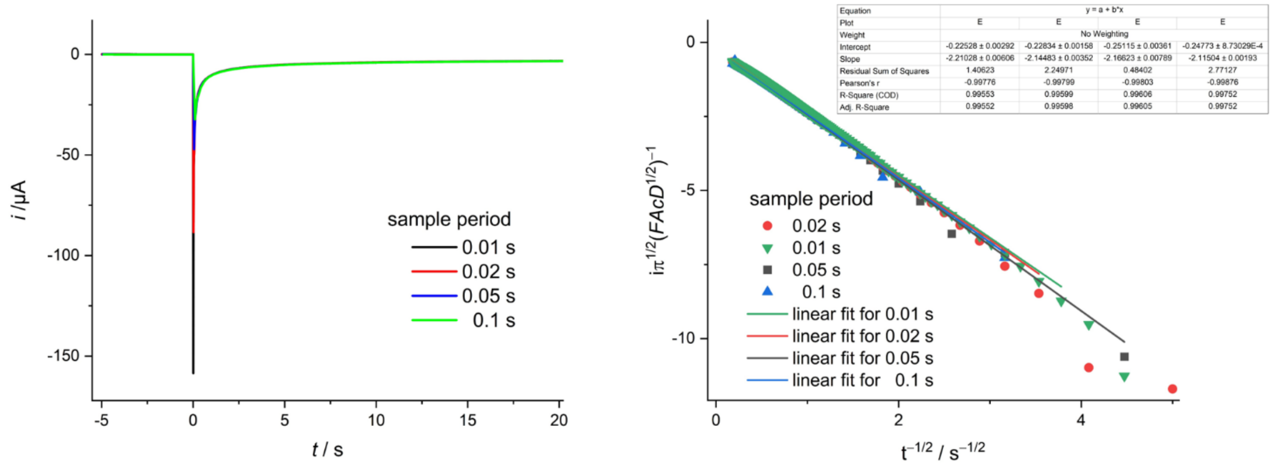

Figure S27: Left: Chronoamperometry at the step potential E = –2.6 V with different sample periods (0.01, 0.02, 0.05, 0.1 s). Right: Cottrell-plots for different sample periods (0.01, 0.02, 0.05, 0.1 s).

Electrochemical Event	Number of Electrons
0.54 V	2
−1.98 V	1
−2.53 V	1

1.5 Excited state thermochemical data of **8**.

1.5.1 Estimation of excited state thermochemical data of **8**.

The excited state oxidation potential (E^*) of **8** was estimated from the ground-state oxidation potential ($E^{0'}$) and the excited state emission energy (E_{00}) *via* the Rehm-Weller equation.[79] An upper limit for $E^{0'}$ was estimated from the irreversible 2-electron oxidation wave, assuming an ECE process with an upper limit for a unimolecular chemical step ($k \lesssim 10^{15}\ s^{-1}$):[8 8]

$$E^{0'} = E_p - \frac{R \cdot T}{n \cdot F}\left[0.780 + \frac{1}{2}\ln\left(\frac{n \cdot F \cdot v}{R \cdot T \cdot k}\right)\right] \lesssim 0.85\ V$$

E_p: peak potential, R: ideal gas constant, T: temperature, n: number of electrons, F: Faraday constant, v: scan rate

The energy of the thermalized phosphorescent state relative to the vibronic ground state (E_{00} = 633 nm) was estimated from the emission spectrum (Figure S7) as the x-intercept of a linear extrapolation of the high energy edge. Thus, E^* was estimated as:

$$E^* \approx E^{0'} - E_{00} \lesssim -1.1\ \mathrm{V}$$

Likewise, the excited state Ir–H bond strength $BDFE^*_{\text{Ir–H}}$ was estimated from the computed ground state BDFE (see section 5) and E_{00}:

$$\mathrm{BDFE_{Ir-H}}^* \approx \mathrm{BDFE_{Ir-H}} - E_{00} \approx 21\ \mathrm{kcal \cdot mol^{-1}}$$

2 Self-sensitized photochemical reactions of 3^+

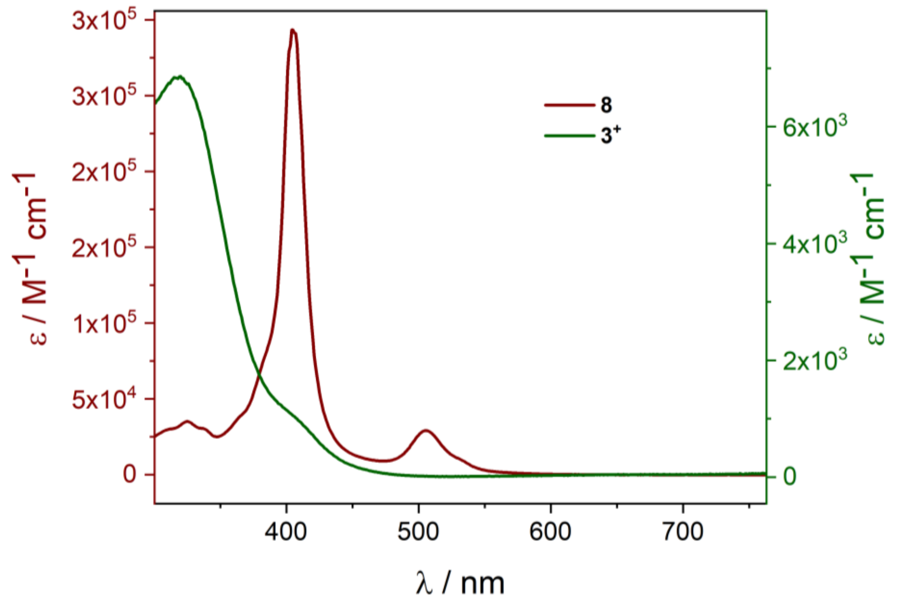

*Figure S28: Comparison of the electronic absorption spectra of **3**($BAr^F{}_{24}$) and **8** in 1,2-difluorobenzene.*

2.1 Photolysis of 3^+ in THF

3($BAr^F{}_{24}$) (6.6 mg, 4.0 µmol, 1.0 eq) is dissolved in THF-d_8 (0.5 mL) and photolyzed for 24 h with a blue LED (456 nm). After photolysis, the solvent is removed *in vacuo* and 1,3,5-trimethoxybenzene is added as internal standard. NMR spectroscopic examination of the product mixture reveals the formation $[ReBr_2(HPNP)PhCN]^+$ as main product (90 %). The addition of (^{n}Bu$_4$N)Br gives **2** and benzonitrile, both in 90% spectroscopic yield.

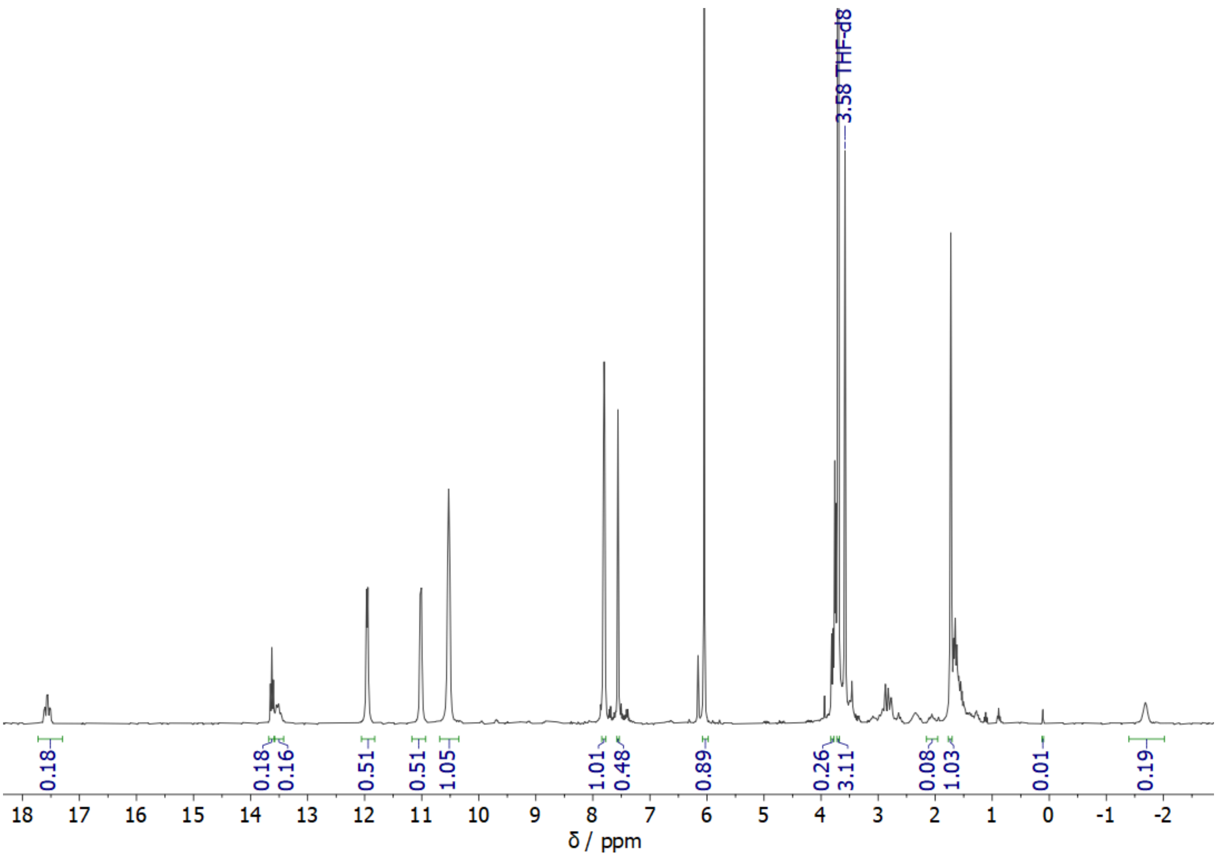

*Figure S29: ^{1}H NMR spectrum after the photolysis of **3**($BAr^F{}_{24}$) in THF-d_8.*

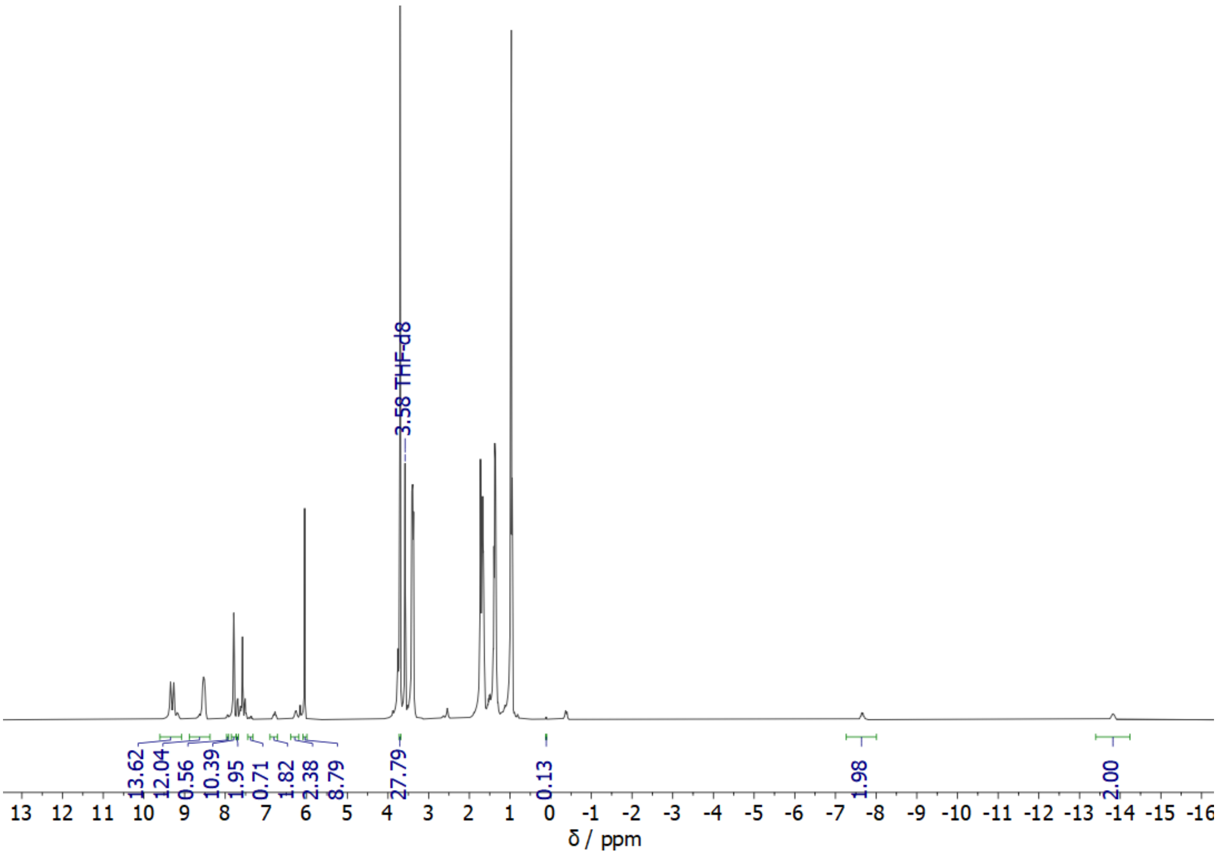

*Figure S30: ^{1}H NMR spectrum after the photolysis of **3**($BAr^F{}_{24}$) in THF-d_8 and subsequent addition of ($^nBu_4N$)Br.*

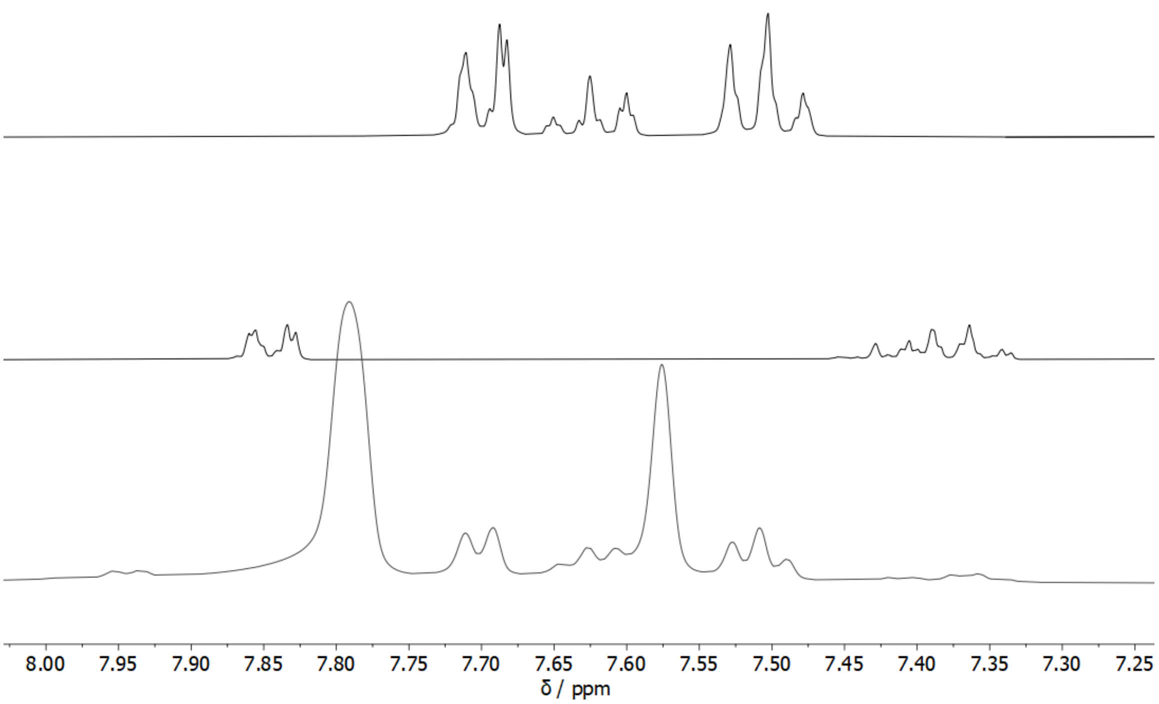

*Figure S31: Bottom: ¹H NMR spectrum after photolysis of **3**(BArF24) in THF-d8 and subsequent addition of (ⁿBu4N)Br. Center: ¹H NMR spectrum of benzamide. Top: ¹H NMR spectrum of benzonitrile.*

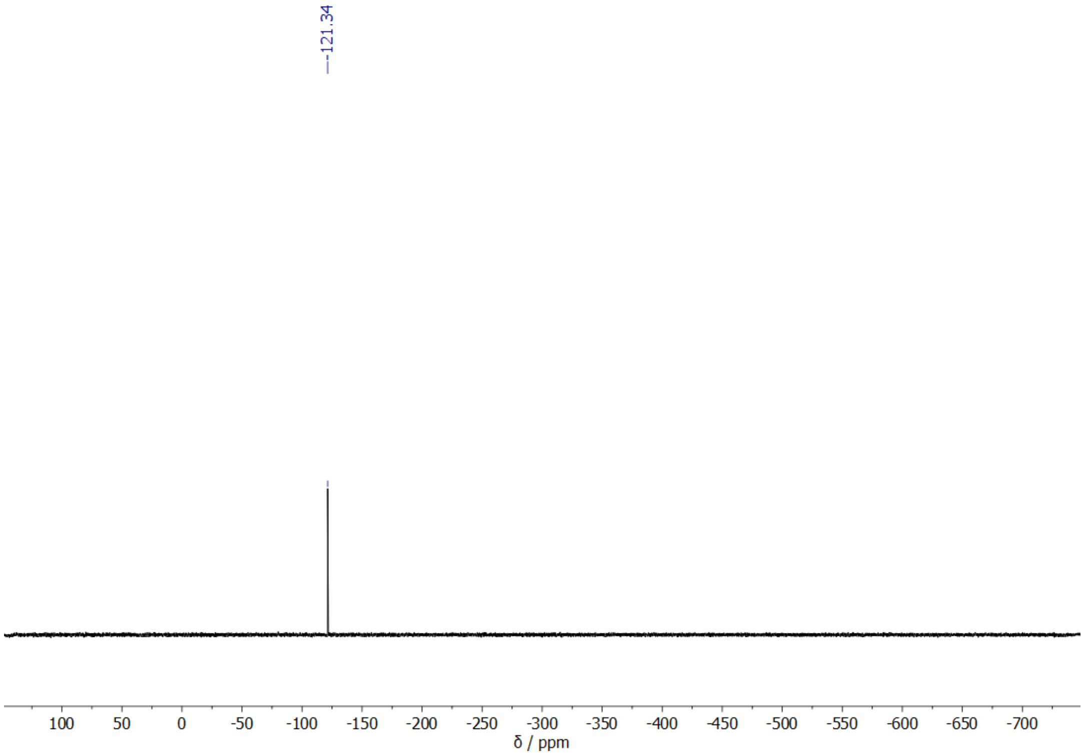

*Figure S32: ¹⁵N{¹H} NMR of the reaction mixture after photolysis of ¹⁵N labeled **3**(BArF24) in THF-d8 and addition of (ⁿBu4N)Br. The spectrum shows at δ=–121.34 ppm benzonitrile.*

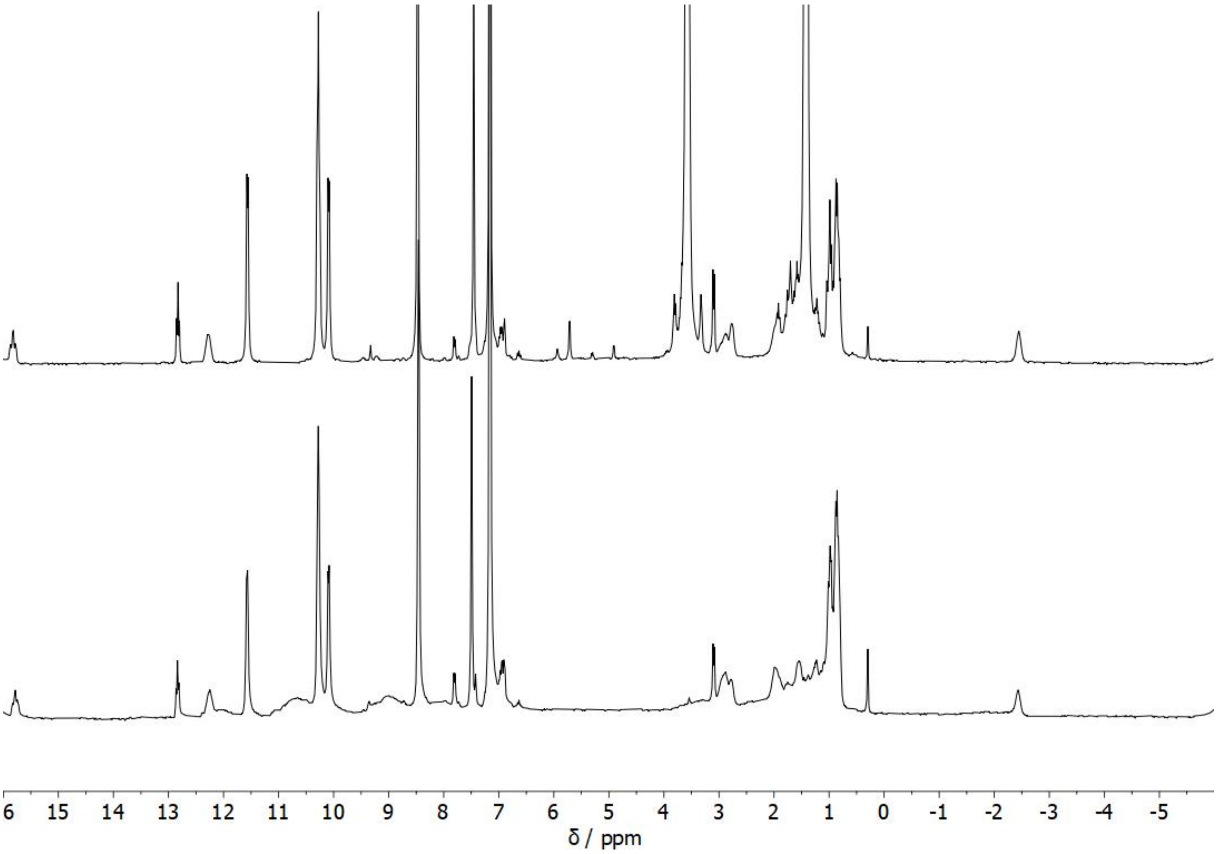

*Figure S33: Comparison of the ^{1}H NMR spectra after photolysis of **3**(BAr$^{F}_{24}$) in THF-d$_8$ (bottom) or THF-h$_8$ (top), respectively, after trap-to-trap removal of the solvent and redissolving in C_6D_6. The signals at δ = 4-6 ppm (top) are indicative of follow-up products from hydroxylated THF (see Figure 29).*

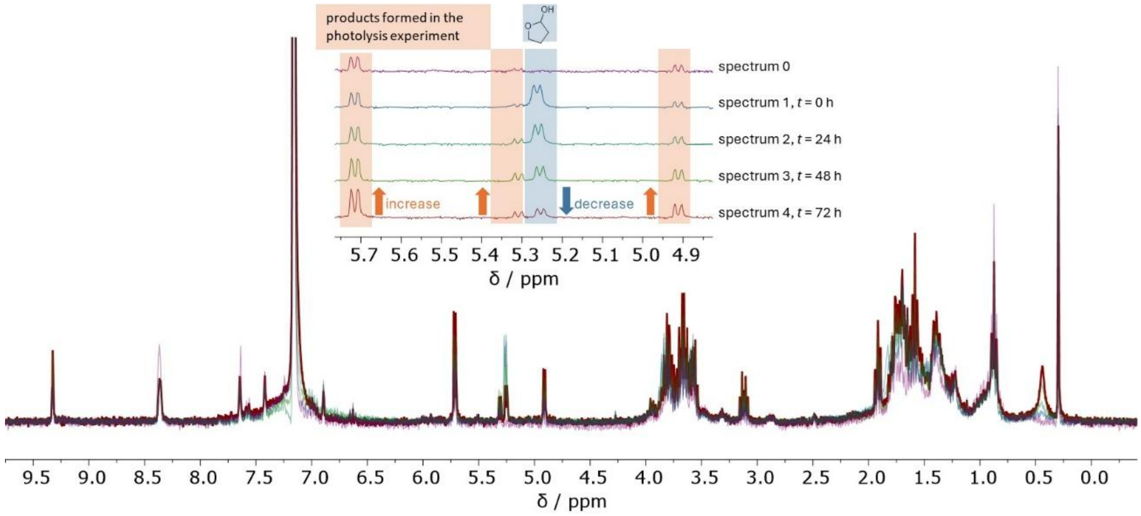

*Figure S34: ^{1}H NMR spectra of the organic side products from photolysis of **3**(BAr$^{F}_{24}$) in THF-h$_8$ and control experiment with 2-hydrohytetrahydrofuran , as potential product from light-induced oxygen transfer:*
After photolysis, the solvent was removed by trap-to-trap transfer. The organic product was then separated upon extraction with pentane. After filtration, the pentane fraction was evaporated, and the residue redissolved in C_6D_6 to obtain the first spectrum (spectrum 0). After that, 2-hydroxytetrahydrofuran was added (spectrum 1). The mixture was monitored by recording NMR spectra every 24 h (spectra 2-5). The decay of 2-hydroxytetrahydrofuran into the same products formed in the photolysis experiment supports the initial formation of 2-hydroxytetrahydrofuran, which is not stable under these conditions.

2.2 Photolysis of **3**$^+$ in 1,2-difluorobenzene

3($BAr^F{}_{24}$) (5.0 mg, 3.1 µmol, 1.0 eq) is dissolved in 1,2-difluorobenzene (0.5 mL) and irradiated for 24 h with a blue LED (456 nm). After photolysis, the solvent is removed in vacuo and ($^nBu_4N$)Br was added and the products were analyzed by nmr spectroscopy, giving **2** (11 %), **7** (3 %), benzonitrile (11 %) and benzamide (~ 5 %) as spectroscopic yield.

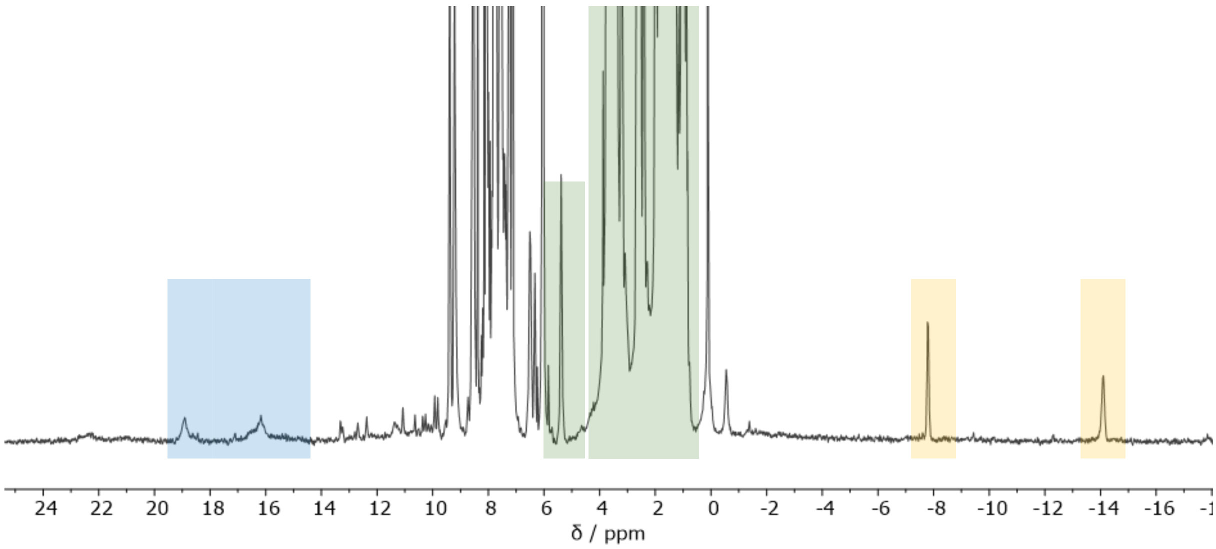

*Figure S35: ^1H NMR spectrum after the photolysis of **3**($BAr^F{}_{24}$) in 1,2-difluorobenzene, removal of the solvent, and addition of THF-d_8, ($^nBu_4N$)Br, and trimethoxybenzene (orange: **2**; blue: **7**; green: **3**).*

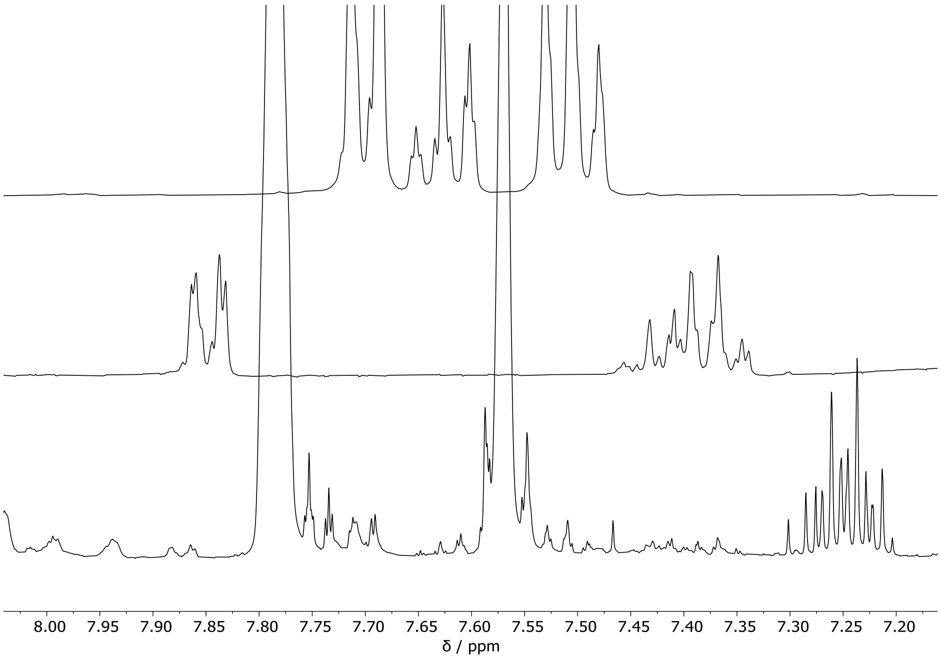

Figure S36: Bottom: Expansion of the ^1H NMR spectrum from Figure S32. Center: ^1H NMR spectrum of benzamide. Top: ^1H NMR spectrum of benzonitrile.

2.3 Photolysis of 3^+ and 1,4-CHD in 1,2-difluorobenzene

3(BAr^F_{24}) (6.6 mg, 4.0 µmol, 1.0 eq) and 1,4-cylohexadiene (1,4-CHD) (3.4 µL, 40 µmol, 10 eq) are dissolved in 1,2-difluorobenzene (0.5 mL) and irradiated with a blue LED (456 nm) for 12 h. $^{31}P\{^1H\}$ NMR spectroscopy showed the formation of two products, which were assigned to *trans*-[Re(HPNP){OC(NH_2)Ph}]$^+$ (*trans*-$\mathbf{5}^+$; δ = –1882 ppm) and *cis*-[Re(HPNP){OC(NH_2)Ph}]$^+$ (*cis*-$\mathbf{5}^+$; δ = –2117 ppm) respectively. The solvent is removed in vacuo and the crude product dissolved in THF-d_8. After the addition of ($^n Bu_4N$)Br (3.0 mg, 9.3 µmol, 2.3 eq) and trimethoxybenzene as internal NMR standard, NMR spectroscopic examination evidenced the formation of **2** (77 %), benzamide (70 %), and benzonitrile (7 %).

Photolysis at 525 nm gave no reaction.

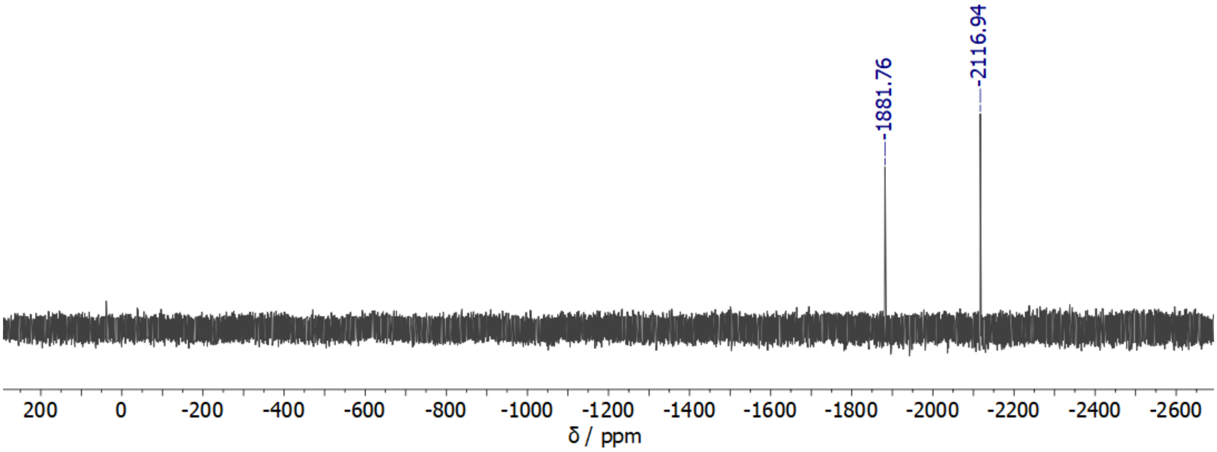

*Figure S37: $^{31}P\{^1H\}$ NMR spectrum directly after photolysis of **3**(BAr^F_{24}) and 1,4-CHD in 1,2-difluorobenzene.*

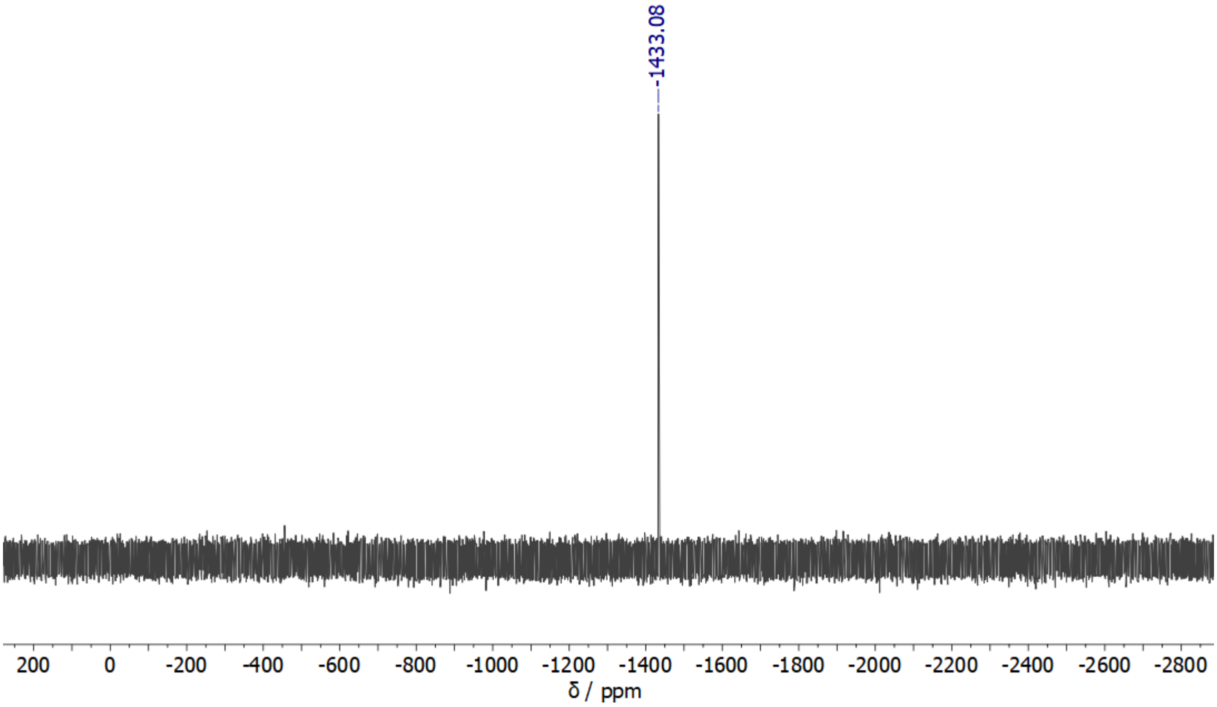

*Figure S38: $^{31}P\{^1H\}$ NMR spectrum after photolysis of **3**(BAr^F_{24}) and 1,4-CHD in 1,2-difluorobenzene, removal of the solvent, and addition of THF-d_8, ($^n Bu_4N$)Br, and trimethoxybenzene.*

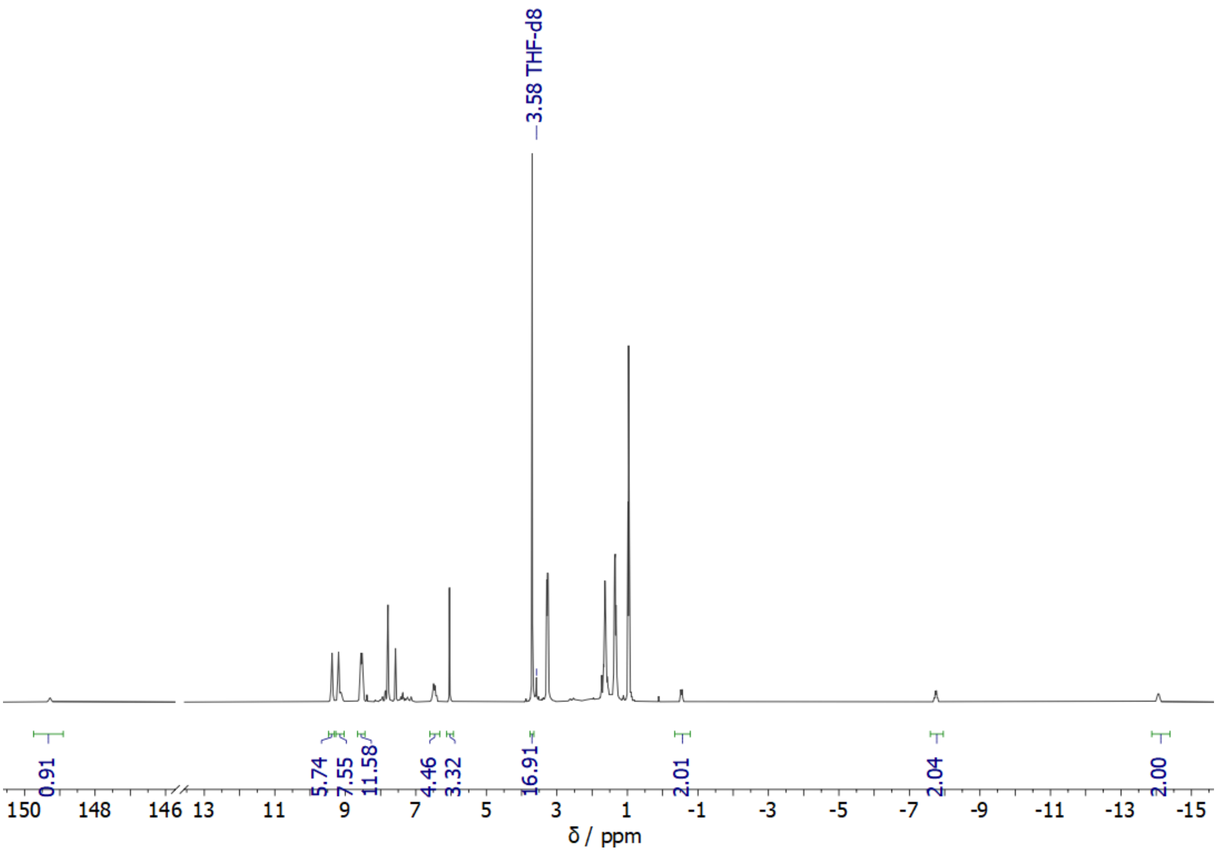

*Figure S39: ^{1}H NMR spectrum after photolysis of **3**(BAr^F$_{24}$) and 1,4-CHD in 1,2-difluorobenzene, removal of the solvent, and addition of THF-d$_8$, (^{n}Bu$_4$N)Br, and trimethoxybenzene.*

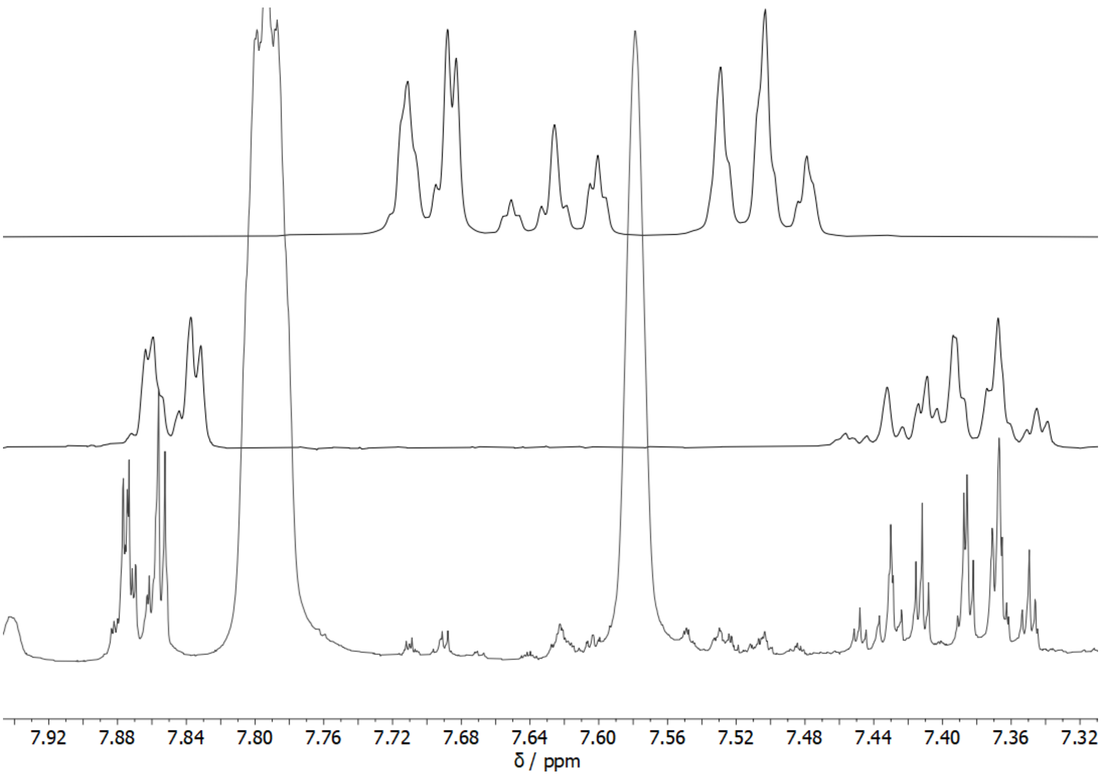

Figure S40: Bottom: Expansion of the ^{1}H NMR spectrum from Figure S39. Center: ^{1}H NMR spectrum of benzamide. Top: ^{1}H NMR spectrum of benzonitrile.

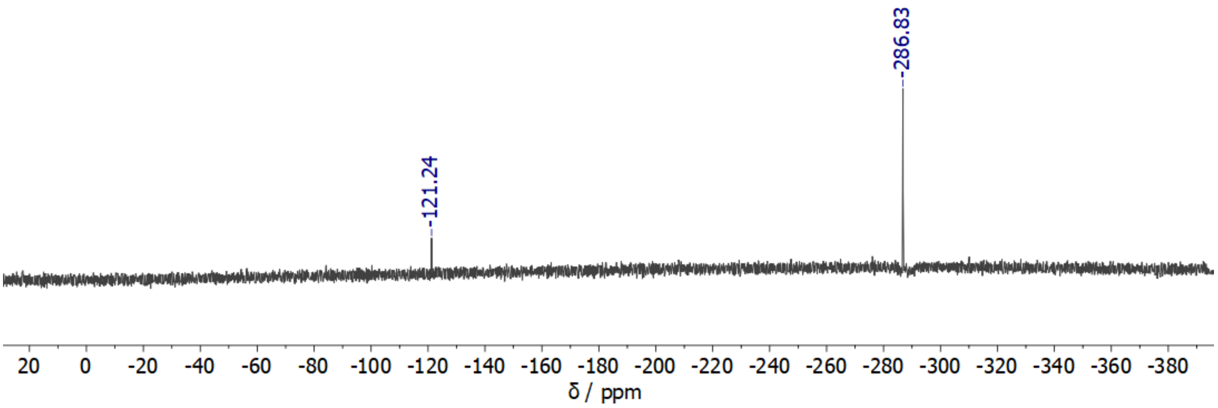

*Figure S41: $^{15}N\{^1H\}$ NMR spectrum after photolysis of **3**($BAr^F{}_{24}$) and 1,4-CHD in 1,2-difluorobenzene, removal of the solvent, and addition of THF-d_8, ($^nBu_4N$)Br, and trimethoxybenzene. The signals are assigned to benzamide (δ= –286.83 ppm) and benzonitrile (δ=–121.24 ppm).*

2.4 Photolysis of $\mathbf{3}^+$ and **8** in 1,2-difluorobenzene

3($BAr^F{}_{24}$) (2.0 mg, 1.2 µmol, 1.0 eq) and **8** (2.0 mg, 2.5 µmol, 2.0 eq) are dissolved in 1,2-difluorobenzene (0.5 mL) and irradiated with a blue LED (525 nm) for 2 h. The solvent is removed in vacuo and the crude product dissolved in THF-d_8. After the addition of ($^nBu_4N$)Br and trimethoxybenzene as internal NMR standard the reaction mixture was analyzed by NMR spectroscopy.

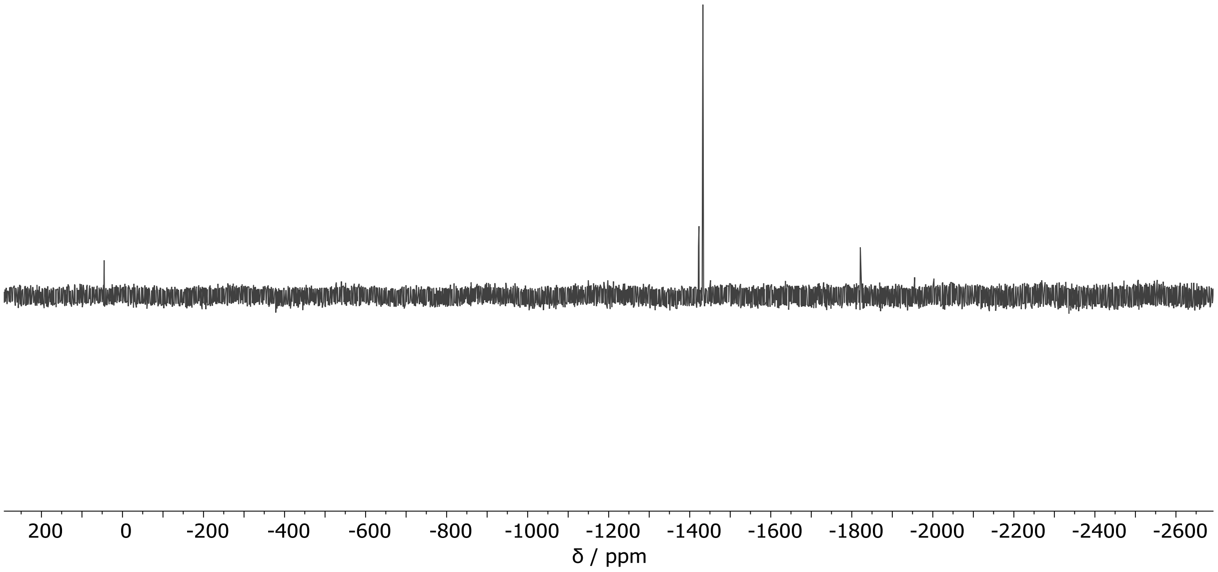

*Figure S42: $^{31}P\{^1H\}$ NMR spectrum after photolysis of **3**($BAr^F{}_{24}$) and **8** in 1,2-difluorobenzene with 525nm, removal of the solvent, and addition of THF-d_8, ($^nBu_4N$)Br, and trimethoxybenzene.*

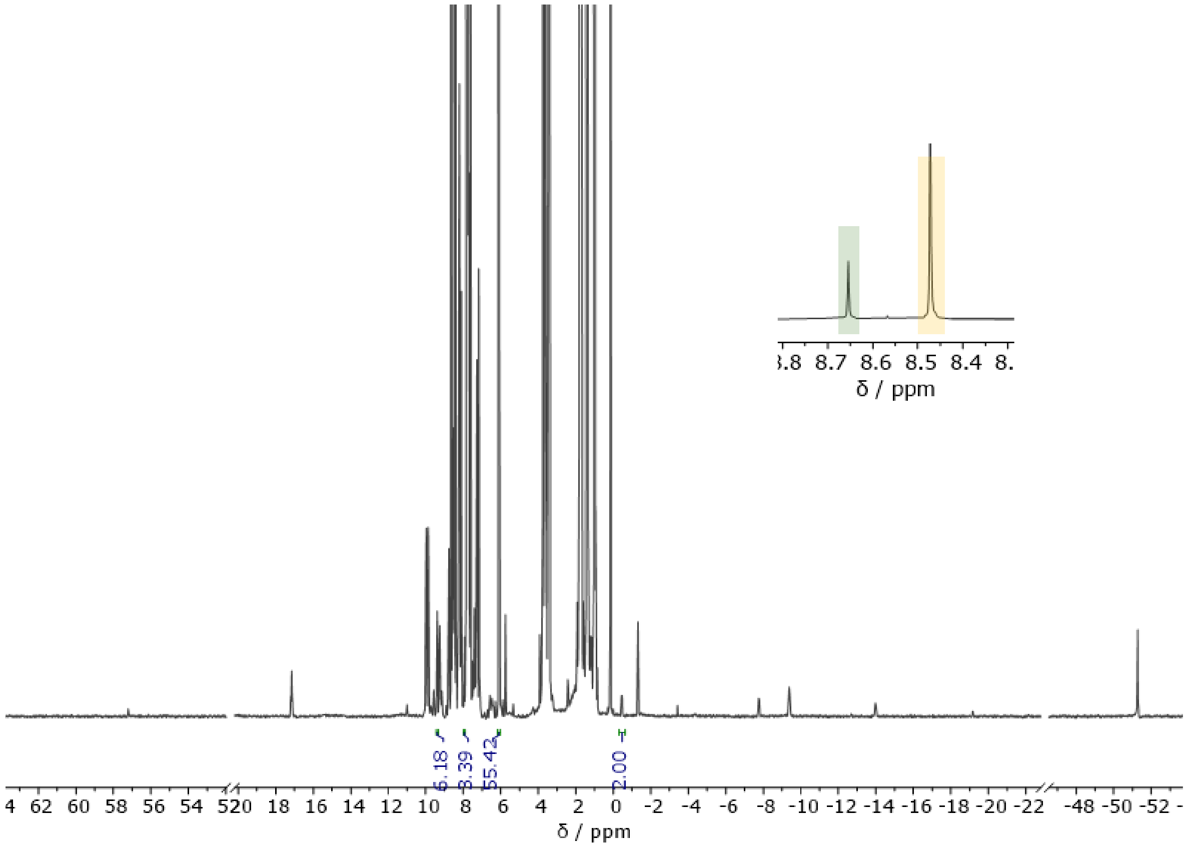

*Figure S43: ^{1}H NMR spectrum after photolysis of **3**(BAr$^F{}_{24}$) and **8** in 1,2-difluorobenzene with 525 nm, removal of the solvent, and addition of THF-d_8, (^{n}Bu$_4$N)Br, and trimethoxybenzene (green: **A**; orange **8**).*

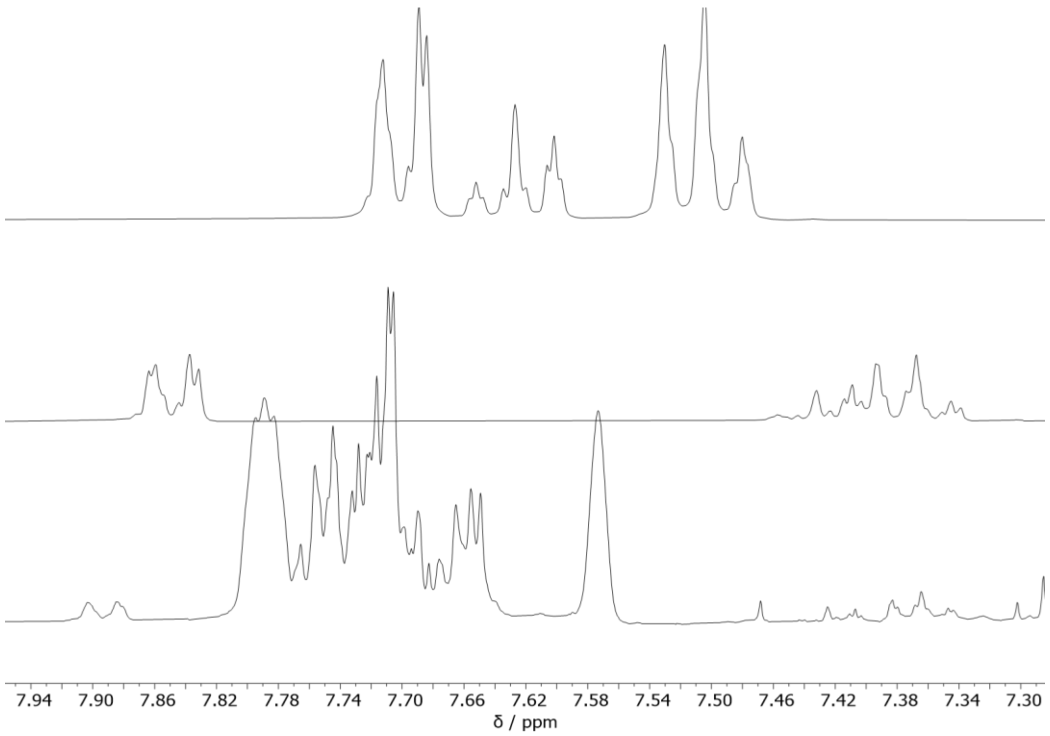

Figure S44: Bottom: Expansion of the ^{1}H NMR spectrum from Figure S40 Center: ^{1}H NMR spectrum of benzamide. Top: ^{1}H NMR spectrum of benzonitrile

2.5 Reaction of 3^+ and 9 in 1,2-difluorobenzene

3(BAr^F_{24}) (2.1 mg, 1.3 µmol, 1.0 eq) and **9** (2.1 mg, 1.3 µmol, 1.0 eq) are dissolved in 1,2-difluorobenzene (0.5 mL) and stirred for 30 minutes at room temperature. The color changed from dark brown to bright red. The solvent is removed in vacuo and the crude product dissolved in THF-d_8. NMR spectroscopic examination evidenced the formation of **8** and an unknown porphyrin product **A** (1:1).

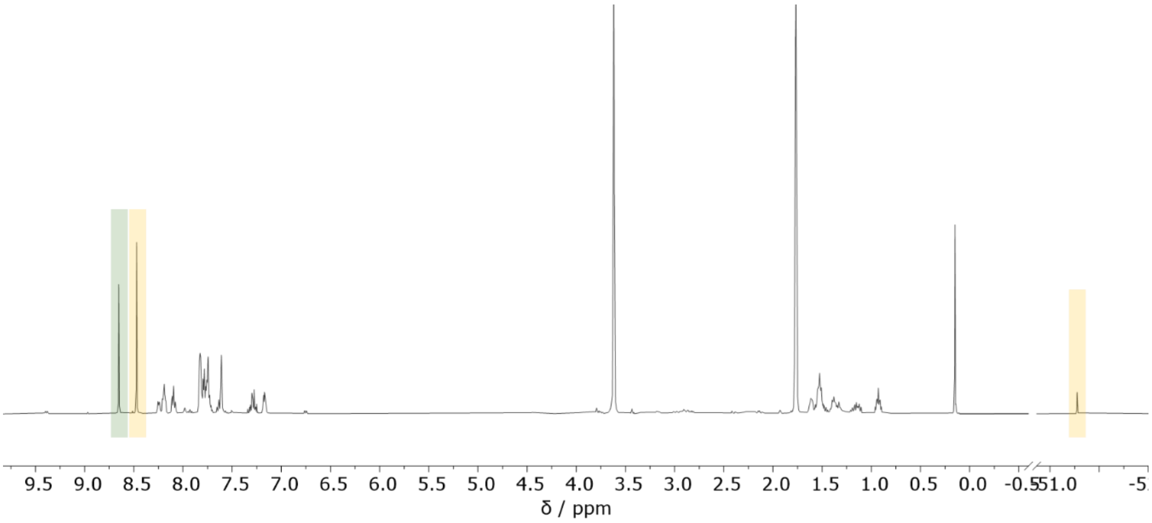

*Figure S45: ^1H NMR spectrum after the reaction of **3**(BAr^F_{24}) and **9** in 1,2-difluorobenzene, removal of the solvent, and addition of THF-d_8 (green: **A**; orange **8**).*

3 Photocatalytic hydrogenation

3.1 Synthetic Procedures

3(BAr^F_{24}) (6.1 mg, 3.7 µmol, 1.0 eq) and **8** (60.2 µg, 0.075 µmol, 0.02 eq) are dissolved in 1,2-difluorobenzene in a J-Young NMR tube. The solution is degassed by two *freeze-pump-thaw* cycles and the reaction vessel is backfilled with H_2 (1 bar). After photolysis (3 h), in case of a reaction a single Re products is observed by $^{31}P\{^1H\}$ NMR spectroscopy (δ = –2117 ppm), which is assigned to [*cis*-[Re(HPNP){OC(NH_2)Ph}]$^+$ (*cis*-**5**$^+$), which could not be isolated, but slowly isomerizes in the dark to *trans*-[Re(HPNP){OC(NH_2)Ph}]$^+$ (*trans*-**5**$^+$, δ = –1882 ppm) over several hours (Figure S46).

For product quantification, the solvent is removed *in vacuo* after photolysis. The residue is dissolved in THF-d_8 (0.5 mL). ($^nBu_4N$)Br (2.5 mg, 7.8 µmol, 2.1 eq) is added for quantitative conversion of **5**$^+$ to **2**, as well as trimethoxybenzene as internal NMR standard. The products were analyzed by NMR spectroscopy (Table S1).

*Table S1: Catalytic benzamide formation using visible light (525 nm): control experiments. All yields refer to **3**(BAr^F_{24}).*

Entry	Reaction conditions (amount of **8** relative to **3**$^+$, photolysis wavelength, temperature, gas atmosphere)	**2** yield / %	$PhCONH_2$ yield / %	PhCN yield / %
1	2mol%, 525nm, 25°C, H_2	98	92	6
2	525nm, 25°C, H_2	0	0	0
3	2mol%, 25°C, H_2	0	0	0
4	2mol%, 525nm, 25°C, Ar	2	0	0
5	10mol%, 525nm, 25°C, H_2	96	86	8
6	2mol%, 456nm, 25°C, H_2	98	97	2
7	2mol%, 525nm, 70°C, H_2	91	91	5

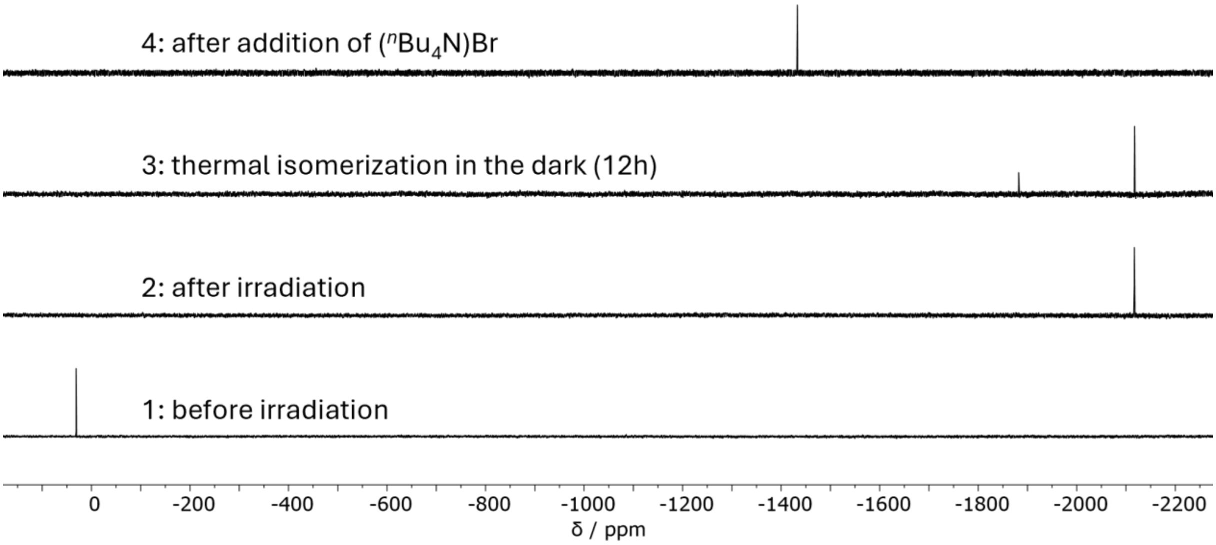

*Figure S46: $^{31}P\{^1H\}$ NMR spectra of the photolysis of **3**(BAr^F_{24}) in 1,2-difluorobenzene in the presence of **8** (2 mol%) under an H_2 atmosphere. Sample 1: before irradiation. Sample 2: directly after irradiation (λ = 525 nm, 100 min) Sample 3: Sample 2 after storage in the dark for 12 h. Sample 4: Sample 3 after the addition of (^{n}Bu$_4$N)Br.*

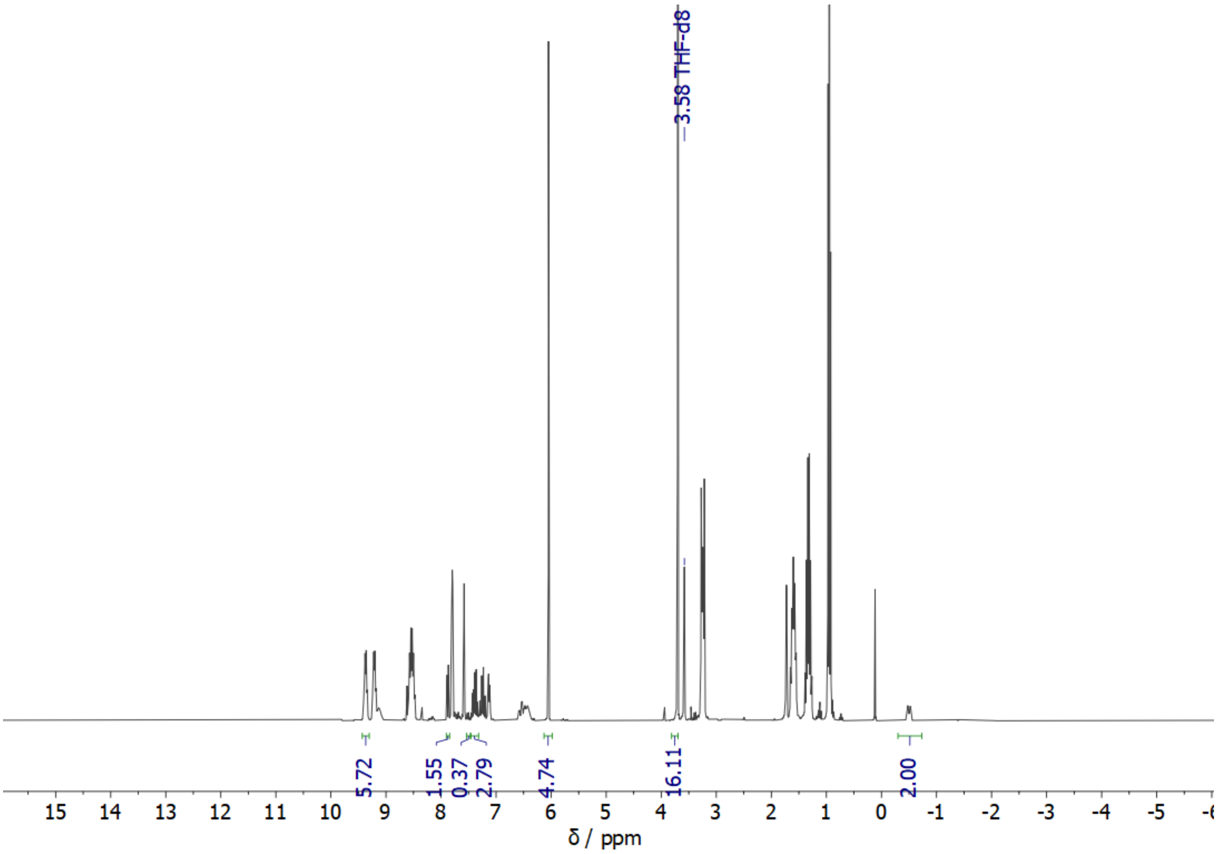

*Figure S47: ^1H NMR spectrum after photolysis of **3**(BAr^F_{24}) and **8** (2 mol%) under H_2 atmosphere in 1,2-difluorobenzene, removal of the solvent, and the addition of THF-d_8, (^{n}Bu$_4$N)Br, and trimethoxybenzene.*

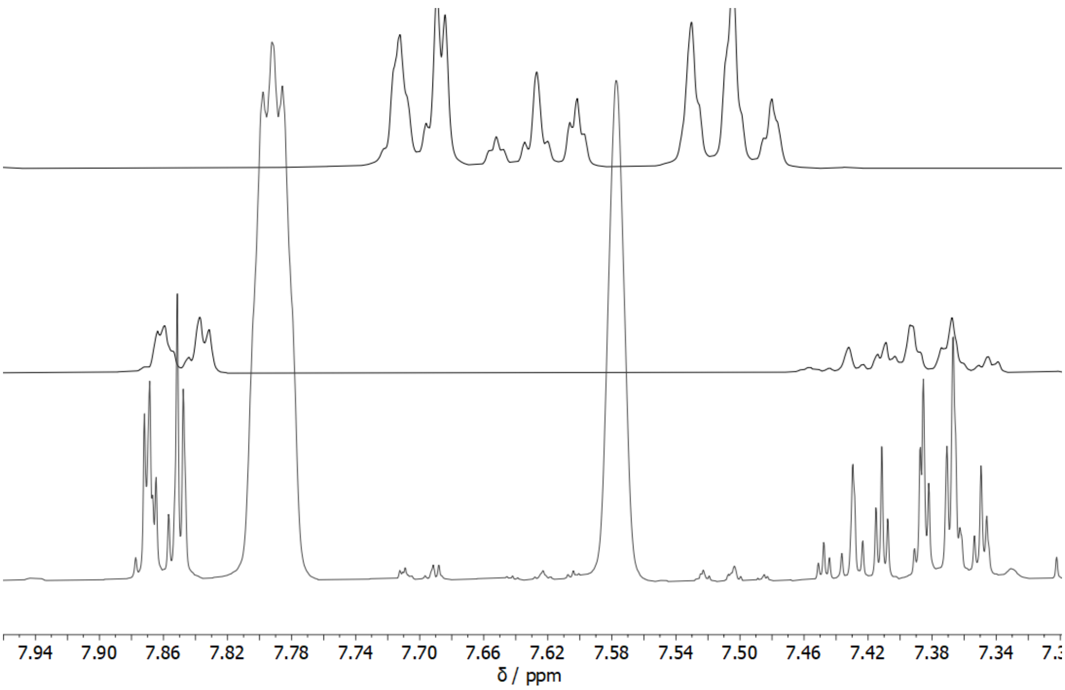

Figure S48: Bottom: Expansion of the ^1H NMR spectrum from Figure S47. Center: ^1H NMR spectrum of benzamide. Top: ^1H NMR spectrum of benzonitrile.

3.2 Isotopic Labelling

^{15}N Labelling

N_2-derived nitrogen transfer was confirmed by use of the ^{15}N labelled imido complex **3**($BAr^F{}_{24}$). After photolysis in 1,2-difluorobenzene, the solvent is removed *in vacuo* and the residue dissolved in THF-*d*$_8$. $^{15}N\{^1H\}$ NMR spectroscopy shows formation of labelled ^{15}N-*trans*-**5**$^+$ (Figure S49). After subsequent addition of ($^nBu_4N$)Br, PhC(O)$^{15}NH_2$ (δ = –285.6 ppm), and minor amounts of PhC^{15}N-(δ = –121.2 ppm) are observed (Figure S50).

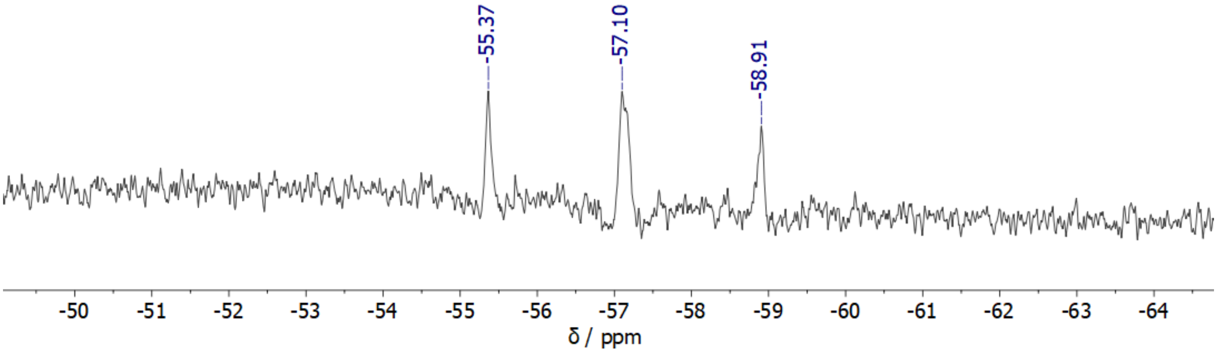

*Figure S49: ^{15}N NMR spectrum after photolysis of ^{15}N-labeled **3**($BAr^F{}_{24}$) in the presence of **8** (2 mol%) under a H_2 atmosphere in 1,2-difluorobenzene, evaporation of the solvent and redissolving THF-d$_8$.*

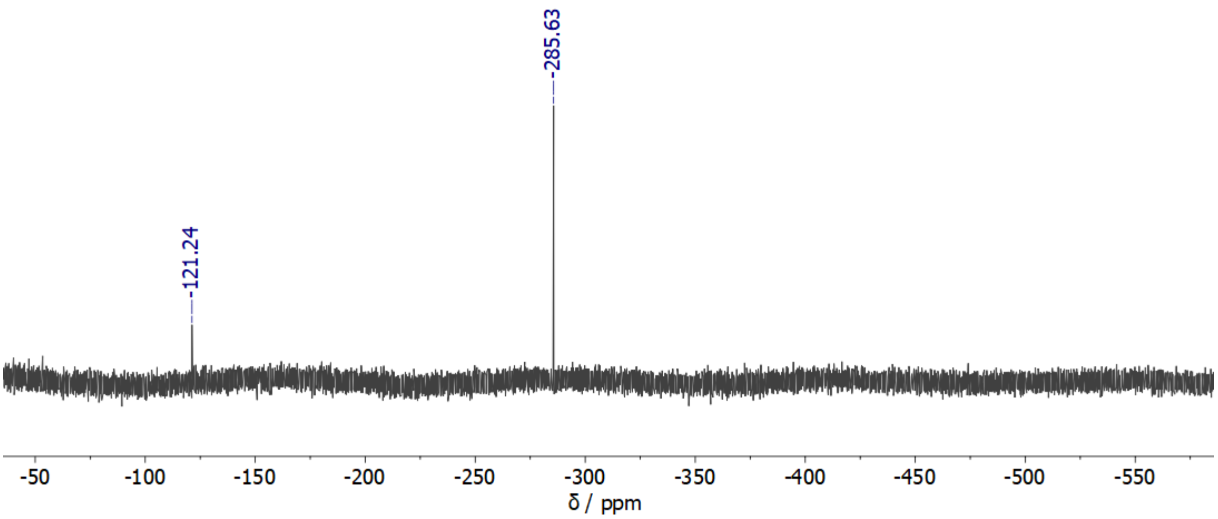

Figure S50: $^{15}N\{^{1}H\}$ NMR after the addition ($^{n}Bu_4N$)Br to the sample from Figure S49.

^{2}H Labelling

The nature of the hydrogen source was confirmed by use of D_2 instead of H_2. After photolysis in 1,2-difluorobenzene, the solvent is removed *in vacuo* and the residue redissolved in THF-h_8. Examination by ^{2}H NMR spectroscopy showed the signals of *trans*-**5**$^{+}$ for coordinated PhC(O)ND_2 and the N–D group of the pincer backbone (Figure S43). Addition of ($^{n}Bu_4N$)Br confirms the formation of [$ReBr_3$(DPNP)] and free PhC(O)ND_2 (Figure S52).

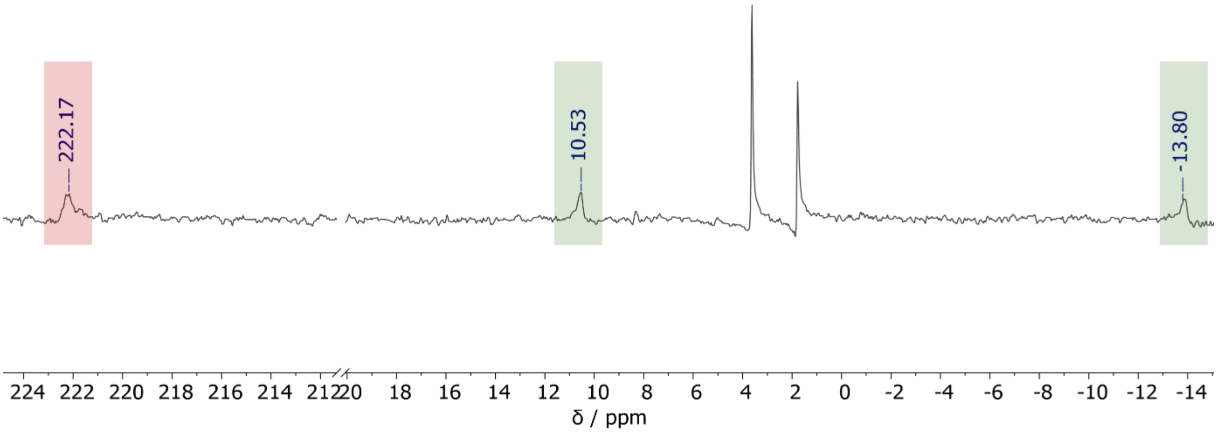

*Figure S51: ^{2}H NMR spectrum after photolysis of ^{15}N-labeled **3**(BAr^{F}_{24}) in the presence of **8** (2 mol%) under a D_2 atmosphere in 1,2-difluorobenzene, evaporation of the solvent, and redissolving THF-h_8. (green: coordinated PhC(O)ND_2; red: N–D group of the pincer backbone of trans-**5**$^{+}$.*

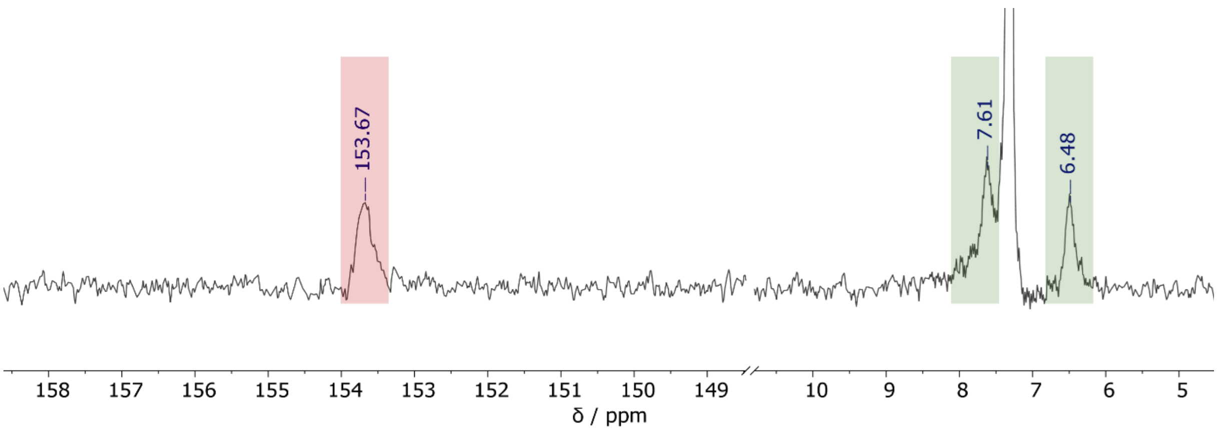

*Figure S52: ^{2}H NMR spectrum after the addition (^{n}Bu$_4$N)Br to the sample from Figure S51 (green: free PhC(O)ND$_2$; red: N–D group of the pincer backbone of **2**.*

3.3 Stern-Volmer Analysis

Stern-Volmer luminescence and lifetime quenching studies were carried out using $1 \cdot 10^{-5}$ M solution of **8** with variable concentrations of **3**(BAr$^F{}_{24}$) in 1,2-difluorobenzene at room temperature. Samples were prepared in a glovebox using a fluorescence quartz cuvette with a J-Young cap. Emission intensities (I) and lifetimes (τ) were measured after each addition of **3**(BAr$^F{}_{24}$). The ratios with intensities in the absence of the quencher (I_0 and τ_0), I_0/I and τ_0/τ, were plotted as a function of quencher concentration c(**3**) (Figure S55). The curvature of the emission intensity plot indicates a contribution from static quenching. From the lifetime plot, a quenching constant $k_q = 6.4 \cdot 10^9$ M^{-1}·s^{-1} was derived.

Identical Stern-Volmer plots were obtained for **8^D** and thus a kinetic isotope effect KIE = 1 for quenching with **3**(BAr$^F{}_{24}$).

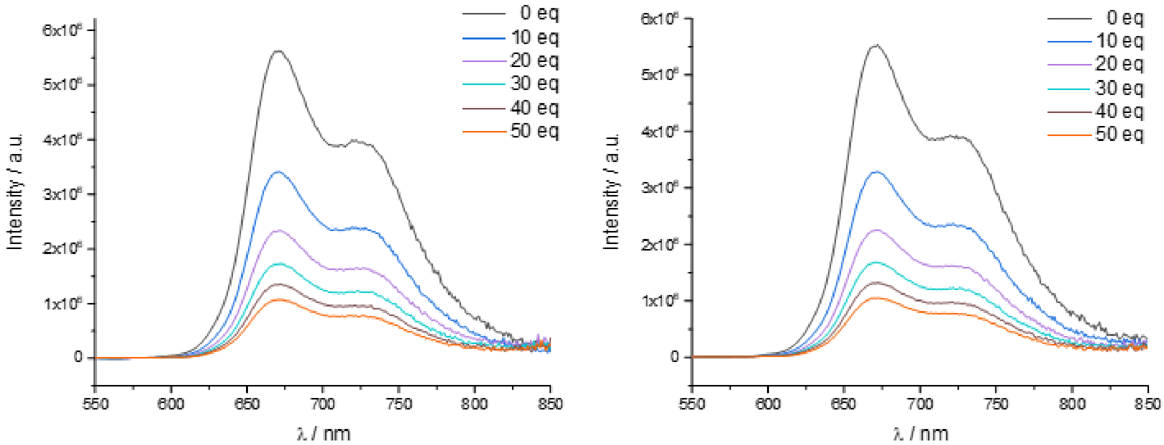

*Figure S53: Luminescence quenching of **8** (left) and **8^D** (right) with **3**(BAr$^F{}_{24}$) in 1,2-difluorobenzene.*

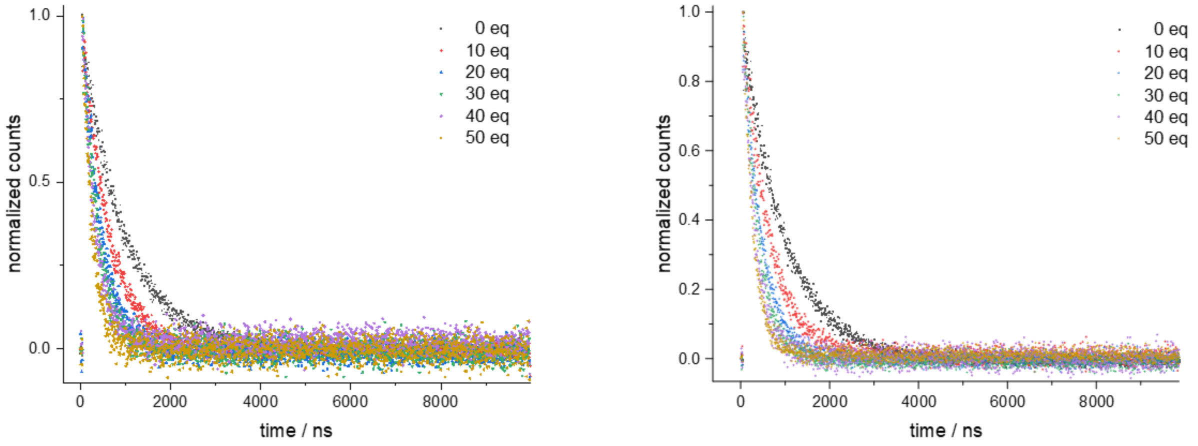

*Figure S54: Time-dependent emission quenching of **8** (left) and **8**^{D} (right) with **3**(BAr$^F{}_{24}$) in 1,2-difluorobenzene.*

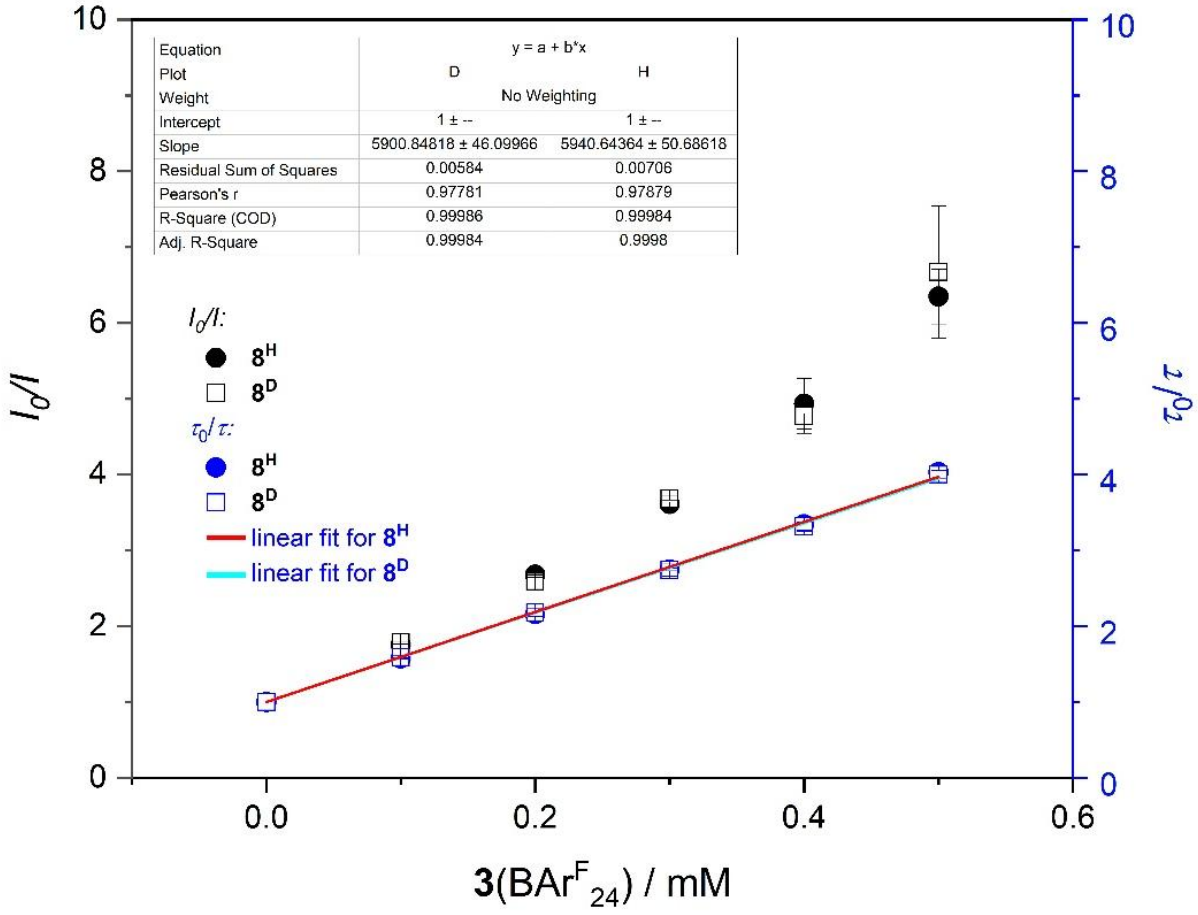

*Figure S55: Stern-Volmer luminescence (black) and lifetime (blue) quenching studies of **8** (circles) and **8**^{D} (squares) with **3**(BAr$^F{}_{24}$) in 1,2-difluorobenzene.*

3.4 Quantum Yields

The quantum yield was derived either via a photolysis experiment inside an NMR spectrometer using a fiber optic setup from Mountain Photonics, as described in section 1.2 or outside the NMR spectrometer with a blue LED. Each experiment was carried out twice.

3.4.1 Quantum yield of the photoconversion of **3**($BAr^F{}_{24}$) to **6**($BAr^F{}_{24}$)

The photon flux of the 450nm LED (I = 3.4·10^{-8} mol·s^{-1}) is determined by photoisomerization of the actinometer 2,4-dinitrobenzaldehyde with a quantum yield of ϕ = 0.08 (λ = 440nm).[9] **3**($BAr^F{}_{24}$) (6.8 mg, 13.9 mM) is dissolved in THF-d_8 and the mixture is photolyzed in the NMR spectrometer for 300 s at room temperature. The absorbance (A_{450}) is determined by UV-vis spectroscopy. The quantum yield is calculated from the following equation:

$$\phi_{450} = \frac{\Delta n}{I \cdot t \cdot (1 - 10^{-A_{450}})} = (0.11 \pm 0.08)\%$$

3.4.2 Quantum yield of the photoconversion of **3**($BAr^F{}_{24}$) with 1,4-CHD

The photon flux of the 427nm LED (I = 2.8·10^{-6} mol·s^{-1}) is determined by photoisomerization of the actinometer 2,4-dinitrobenzaldehyde with a quantum yield of ϕ = 0.08 (λ = 440nm).[9] The photon flux at 427 nm is converted to the 456 nm LED (I = 3.1·10^{-6} mol·s^{-1}), taking into account the output energies of the LEDs. **3**($BAr^F{}_{24}$) (7.0 mg, 4.3 µmol, 1.0 eq) and 1,4-cyclohexadiene (3.4 mg, 3.7 µL, 43 µmol, 10 eq) are dissolved in 1,2-difluorobenzene (0.4 mL) and triphenylphosphine is added as external standard. The mixture is photolyzed with a 456 nm LED for five minutes at room temperature. The absorbance (A_{456}) is determined by UV-vis spectroscopy. The quantum yield is calculated from the following equation:

$$\phi_{456} = \frac{\Delta n}{I \cdot t \cdot (1 - 10^{-A_{456}})} = (0.02 \pm 0.008)\%$$

3.4.3 Quantum yield of the photohydrogenation of **3**($BAr^F{}_{24}$) with photocatalyst **8**

The photon flux of the 427nm LED (I = 2.8 · 10^{-6} mol s^{-1}) is determined using photoisomerization of the actinometer 2,4-dinitrobenzaldehyde with a quantum yield of ϕ = 0.08 (λ = 440nm).[9] The photon flux at 427 nm is converted to the 525 nm LED (I = 1.5·10^{-6} mol·s^{-1}), taking into account the output energies of the LEDs. **3**($BAr^F{}_{24}$) (5.0 mg, 3.1 µmol, 1.0 eq) and **8** (24 µg, 0.31 µmol, 0.1 eq) are dissolved in 1,2-difluorobenzene (0.3 mL), triphenylphosphine is added as external standard. The solution is degassed via 2 freeze-pump-thaw cycles and the vessel is backfilled with H_2. The mixture is photolyzed with a 525 nm LED for three minutes at room temperature. The absorbance (A_{525}) is determined by UV-vis spectroscopy. The quantum yield is calculated from the following equation:

$$\phi_{525} = \frac{\Delta n}{I \cdot t \cdot (1 - 10^{-A_{525}})} = (0.11 \pm 0.10)\%$$

4 Transient Absorption Spectroscopy

4.1 Transient Absorption Spectroscopy of **3**⁺

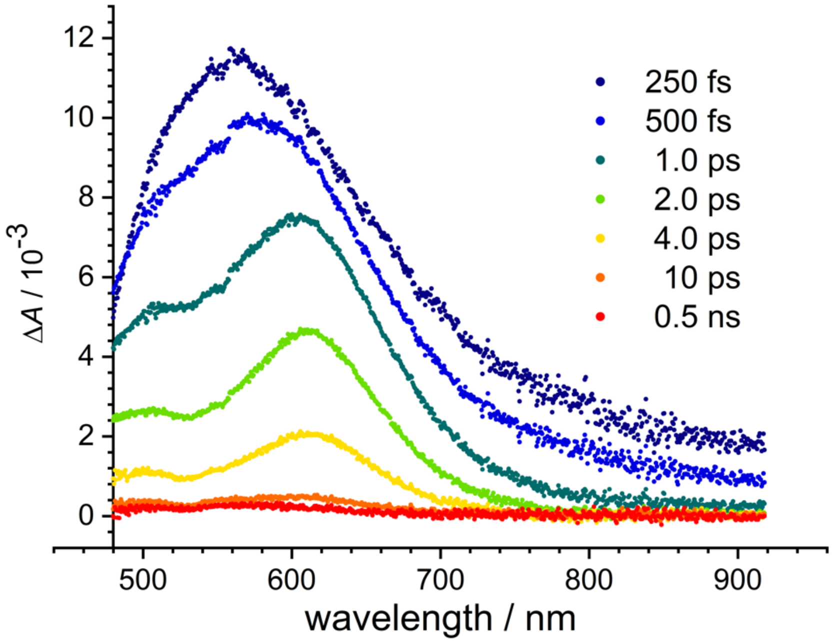

*Figure S56:Transient absorption spectra of **3⁺** in 1,2-difluorobenzene (c = 5 mmol/L) after excitation at 400 nm.*

4.2 Transient Absorption Spectroscopy of **8**

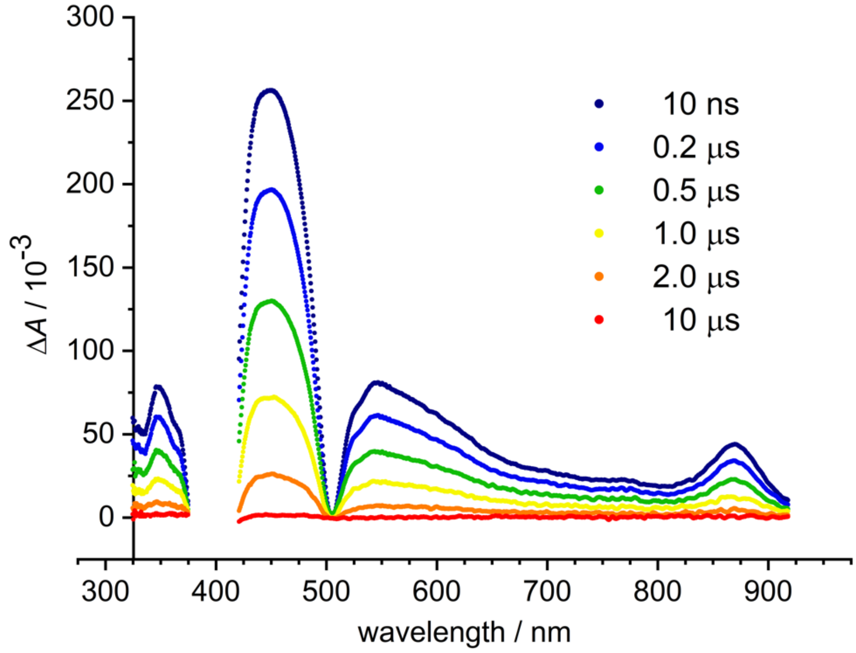

*Figure S57: Transient absorption spectra of **8** in 1,2-difluorobenzene (c = 0.33 mmol/L) after excitation at 505 nm.*

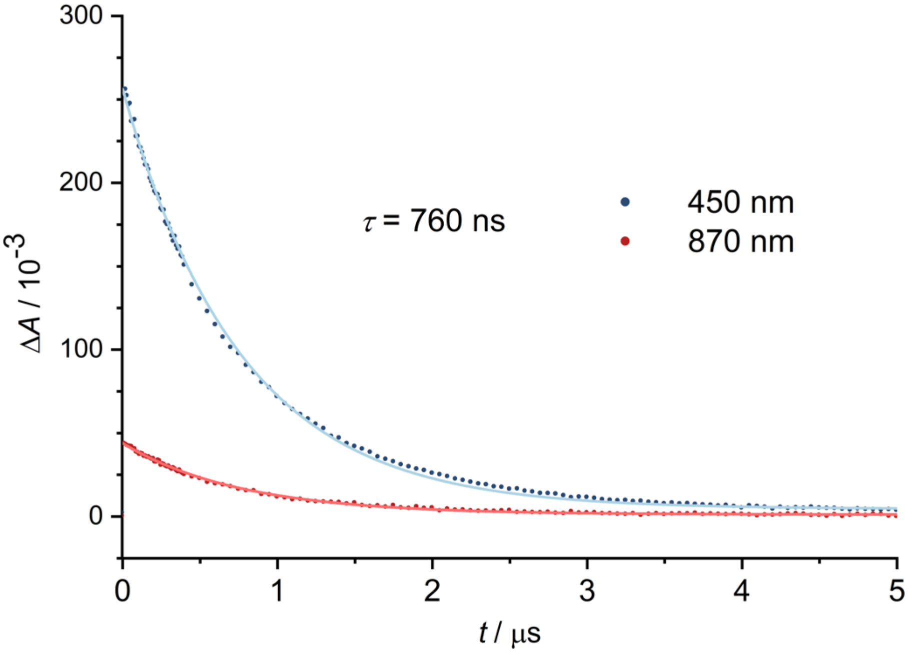

*Figure S58: Kinetic trace (circles) and monoexponential fit (line) of **8** (c = 0.33 mmol/L) in 1,2-difluorobenzene at 450 nm (blue) and 870 nm (red) upon excitation at 505 nm. The resulting excited state lifetime of 760 ns is significantly shorter than the emission lifetime of 1.0 μs. The discrepancy could be explained by the high excited state concentration during the TA-measurement, which allows for additional higher order deactivation pathways, such as triplet-triplet annihilation. This interpretation is also supported by the minor deviation of the decay curves from purely monoexponential behavior.*

4.3 Transient Absorption Spectroscopy of **8** with **3⁺**

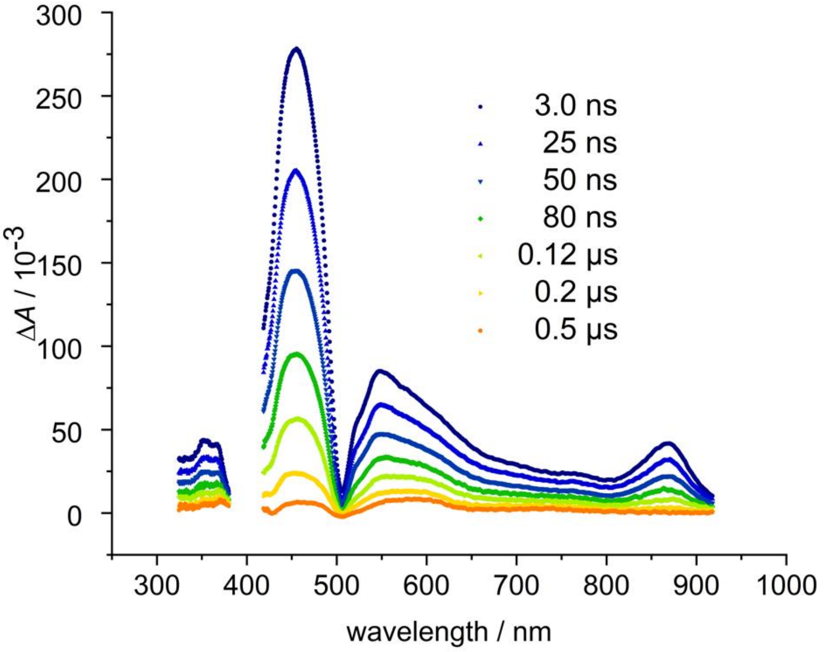

*Figure S59: Transient absorption spectra of **8** (c = 0.33 mmol/L) and **3⁺** (c = 2.5 mmol/L) in 1,2-difluorobenzene after excitation at 505 nm.*

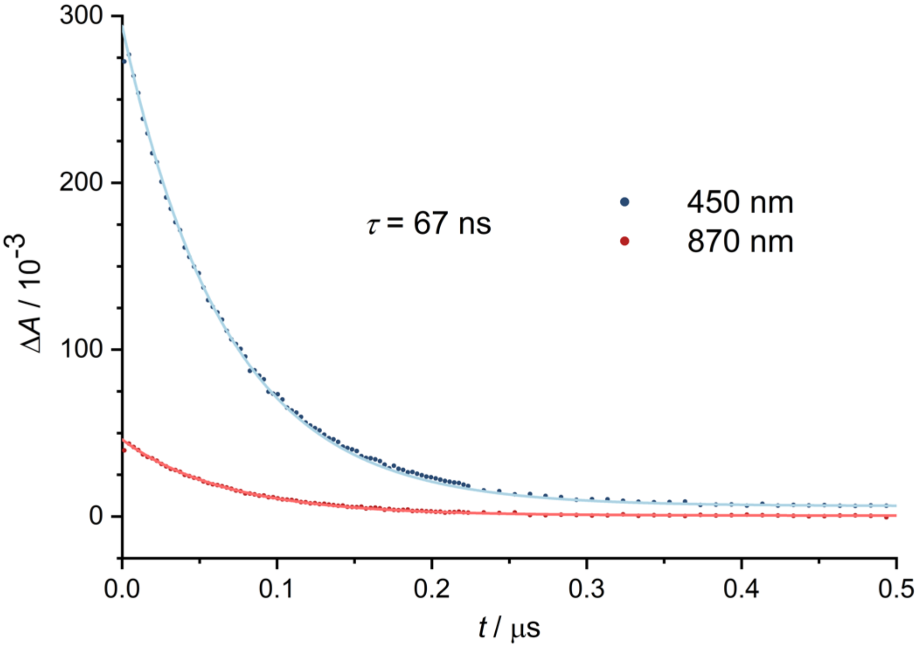

*Figure S60: Kinetic trace (circles) and monoexponential fit (line) of the transient absorption spectra of **8** (c = 0.33 mmol/L) and **3**+ (c=2.5 mmol/L) in 1,2-difluorobenzene at 450 nm (blue) and 870 nm (red) upon excitation at 505 nm.*

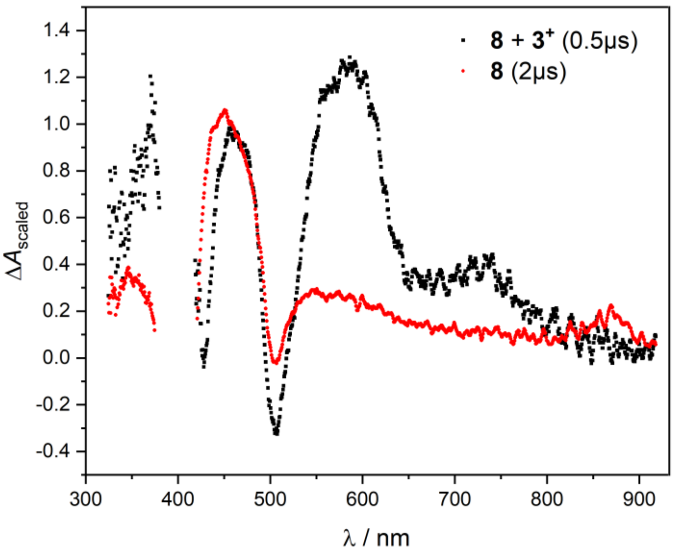

*Figure S61: Comparison of the transient absorption spectra of **8** (at 2µs) with **8**+**3**+ (at 0.5µs).*

4.4 Transient Absorption Spectroscopy of Br_2 in 1,2-difluorobenzene

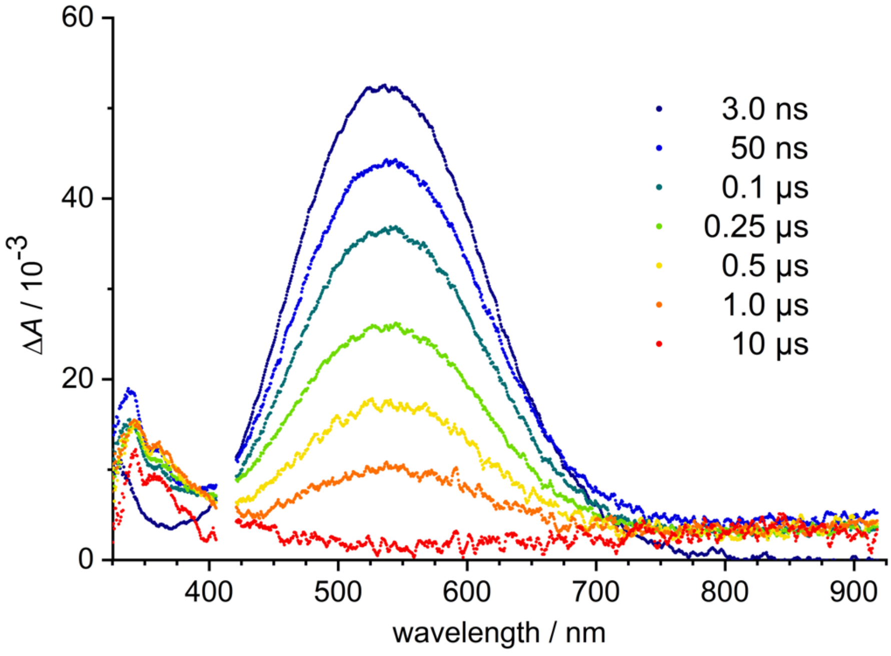

Figure S62: Transient absorption spectra of Br_2 in 1,2-difluorobenzene (c = 0.02 mol/L) after excitation at 415 nm.

5 Crystallographic Details

CCDC 2440224 (**6**(BAr^F_{24})) and 2440225 (*trans*-**5**(BAr^F_{24})) contain the supplementary crystallographic data for this paper. These data can be obtained free of charge from https://www.ccdc.cam.ac.uk/structures/ (or from Cambridge Crystallographic Data Centre, 12 Union Road, Cambridge, CB2 1EZ, UK. Fax: +44-1223-336-033; e-mail: deposit@ccdc.cam.ac.uk).

Suitable single crystals for X-ray structure determination were selected from the mother liquor under an inert gas atmosphere and transferred in protective perfluoro polyether oil on a microscope slide. The selected and mounted crystals were transferred to the cold gas stream on the diffractometer. The diffraction data were obtained at 100 K on a Bruker D8 three-circle diffractometer, equipped with a PHOTON III detector and an INCOATEC microfocus source with Quazar mirror optics (Mo-Kα radiation, λ = 0.71073 Å).

The data obtained were integrated with SAINT and a semi-empirical absorption correction from equivalents with SADABS was applied. The structure was solved and refined using the Bruker SHELX 2014 software package.[10,11,12,13] All non-hydrogen atoms were refined with anisotropic displacement parameters. All C-H hydrogen atoms were refined isotropically on calculated positions by using a riding model with their U_{iso} values constrained to 1.5 U_{eq} of their pivot atoms for terminal sp^3 carbon atoms and 1.2 times for all other atoms.

5.1 Crystal Structure of **6**($BAr^F{}_{24}$)·C_5H_{12}

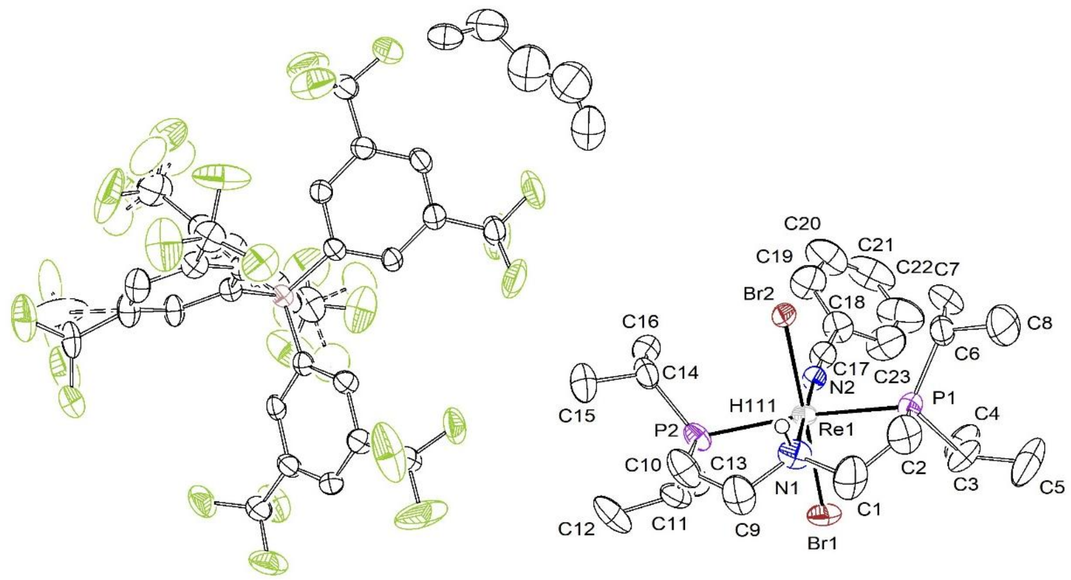

*Figure S63: Thermal ellipsoid plot of **6**($BAr^F{}_{24}$) with the anisotropic displacement parameters drawn at the 50% probability level. The asymmetric unit contains one complex molecule, one disordered counter ion and a half solvent molecule. The disordered counter ion was refined with populations of 0.77(9), 0.67(5) and 0.68(9) on the main domain using some restraints and constraints (SADI, ISOR, FREE). The reflections -1 1 1, -1 0 2, 1 0 2, 1 1 2, -3 0 2, -3 0 4, 0 1 4, 1 1 4, 2 1 4 and 1 1 6 are removed from the refinement using OMIT commands. The asymmetric unit contains two complex molecules. The N-H hydrogen atoms was calculated and constraint to 0.9 Å by using DFIX and SADI and isotropically refined.*

*Table S2: Crystal data and structure refinement for **6**(BAr^F_{24}).*

Identification code	mo_KW_23092001_LZJ_0m_a	
Empirical formula	$C_{115}H_{120}B_2Br_4F_{48}N_4P_4Re_2$	
Formula weight	3307.68	
Temperature	101(2) K	
Wavelength	0.71073 Å	
Crystal system	Monoclinic	
Space group	$P2_1/c$	
Unit cell dimensions	a = 16.0598(7) Å	α= 90°
	b = 16.5505(7) Å	β= 95.692(2)°
	c = 25.4262(12) Å	γ = 90°
Volume	6724.9(5) $Å^3$	
Z	2	
Density (calculated)	1.633 Mg/m^3	
Absorption coefficient	3.147 mm^{-1}	
F(000)	3260	
Crystal size	0.118 x 0.103 x 0.095 mm^3	
Crystal shape and color	Block, clear light orange	
Theta range for data collection	2.461 to 28.299°.	
Index ranges	-21<=h<=21, -22<=k<=22, -33<=l<=33	
Reflections collected	166782	
Independent reflections	16688 [R(int) = 0.0706]	
Completeness to theta = 25.242°	99.8 %	
Refinement method	Full-matrix least-squares on F^2	
Data / restraints / parameters	16688 / 89 / 920	
Goodness-of-fit on F^2	1.159	
Final R indices [I>2sigma(I)]	R1 = 0.0680,	wR2 = 0.1405
R indices (all data)	R1 = 0.0866,	wR2 = 0.1525
Largest diff. peak and hole	2.527 and -2.049 $eÅ^{-3}$	

*Table S3: Bond lengths [Å] and angles [°] for **6**(BAr^F_{24}).*

Re(1)-N(2)	2.020(5)	C(33)-C(34)	1.390(9)
Re(1)-N(1)	2.165(6)	C(34)-C(35)	1.384(10)
Re(1)-P(1)	2.4471(19)	C(34)-C(38)	1.493(11)
Re(1)-P(2)	2.4582(19)	C(35)-C(36)	1.384(10)
Re(1)-Br(1)	2.4635(8)	C(36)-C(37)	1.397(9)
Re(1)-Br(2)	2.4903(7)	C(36)-C(39)	1.491(10)
P(1)-C(6)	1.830(8)	C(38)-F(8)	1.280(9)
P(1)-C(2)	1.837(8)	C(38)-F(9)	1.315(9)
P(1)-C(3)	1.843(8)	C(38)-F(7)	1.362(11)
P(2)-C(10)	1.840(8)	C(39)-F(11)	1.318(10)
P(2)-C(14)	1.849(9)	C(39)-F(12)	1.321(10)
P(2)-C(11)	1.854(8)	C(39)-F(10)	1.339(9)
N(1)-C(1)	1.429(11)	C(40)-C(45)	1.395(9)
N(1)-C(9)	1.514(11)	C(40)-C(41)	1.402(9)
N(1)-H(111)	0.90(2)	C(40)-B(1)	1.636(9)
N(2)-C(17)	1.142(9)	C(41)-C(42)	1.393(9)
C(1)-C(2)	1.504(14)	C(42)-C(43)	1.371(10)
C(3)-C(5)	1.505(13)	C(42)-C(46)	1.509(10)
C(3)-C(4)	1.535(13)	C(43)-C(44)	1.377(11)
C(6)-C(7)	1.514(14)	C(44)-C(45)	1.390(10)
C(6)-C(8)	1.531(13)	C(44)-C(47)	1.490(15)
C(9)-C(10)	1.431(13)	C(44)-C(47A)	1.67(5)
C(11)-C(12)	1.511(12)	C(46)-F(15)	1.316(9)
C(11)-C(13)	1.529(12)	C(46)-F(13)	1.331(9)
C(14)-C(15)	1.521(11)	C(46)-F(14)	1.336(9)
C(14)-C(16)	1.527(12)	C(48)-C(53)	1.396(9)
C(17)-C(18)	1.435(9)	C(48)-C(49)	1.397(10)
C(18)-C(23)	1.369(13)	C(48)-B(1)	1.643(9)
C(18)-C(19)	1.374(13)	C(49)-C(50)	1.395(9)
C(19)-C(20)	1.375(14)	C(50)-C(51)	1.380(12)
C(20)-C(21)	1.309(19)	C(50)-C(54)	1.506(14)
C(21)-C(22)	1.325(19)	C(51)-C(52)	1.381(12)
C(22)-C(23)	1.380(14)	C(52)-C(53)	1.383(10)
C(24)-C(25)	1.399(9)	C(52)-C(55)	1.508(11)
C(24)-C(29)	1.405(9)	C(54)-F(21A)	1.07(3)
C(24)-B(1)	1.639(10)	C(54)-F(19)	1.253(14)
C(25)-C(26)	1.388(9)	C(54)-F(20A)	1.30(3)
C(26)-C(27)	1.373(10)	C(54)-F(20)	1.360(17)
C(26)-C(30)	1.500(10)	C(54)-F(21)	1.38(2)
C(27)-C(28)	1.385(10)	C(54)-F(19A)	1.45(3)
C(28)-C(29)	1.395(10)	C(55)-F(22A)	1.18(2)
C(28)-C(31)	1.516(10)	C(55)-F(23)	1.291(13)
C(30)-F(1)	1.286(10)	C(55)-F(24)	1.295(12)
C(30)-F(2)	1.300(9)	C(55)-F(23A)	1.35(3)
C(30)-F(3)	1.358(11)	C(55)-F(22)	1.350(13)
C(31)-F(6)	1.327(10)	C(55)-F(24A)	1.54(3)
C(31)-F(5)	1.333(9)	C(90)-C(91)	1.34(4)
C(31)-F(4)	1.336(9)	C(91)-C(92)	1.12(4)
C(32)-C(37)	1.390(9)	C(92)-C(93)	1.44(4)
C(32)-C(33)	1.404(9)	C(93)-C(94)	1.32(3)
C(32)-B(1)	1.635(9)	F(16)-C(47A)	1.23(9)

F(16)-C(47)	1.367(15)
F(17)-C(47)	1.288(18)
F(18)-C(47)	1.42(2)
F(17A)-C(47A)	1.23(6)
F(18A)-C(47A)	1.32(5)
N(2)-Re(1)-N(1)	178.2(2)
N(2)-Re(1)-P(1)	96.97(17)
N(1)-Re(1)-P(1)	81.86(19)
N(2)-Re(1)-P(2)	99.57(17)
N(1)-Re(1)-P(2)	81.60(19)
P(1)-Re(1)-P(2)	163.46(6)
N(2)-Re(1)-Br(1)	92.70(16)
N(1)-Re(1)-Br(1)	88.70(19)
P(1)-Re(1)-Br(1)	90.48(5)
P(2)-Re(1)-Br(1)	88.72(5)
N(2)-Re(1)-Br(2)	91.11(16)
N(1)-Re(1)-Br(2)	87.49(19)
P(1)-Re(1)-Br(2)	88.68(5)
P(2)-Re(1)-Br(2)	91.02(5)
Br(1)-Re(1)-Br(2)	176.17(3)
C(6)-P(1)-C(2)	104.0(4)
C(6)-P(1)-C(3)	110.3(4)
C(2)-P(1)-C(3)	106.0(5)
C(6)-P(1)-Re(1)	118.4(3)
C(2)-P(1)-Re(1)	98.8(3)
C(3)-P(1)-Re(1)	116.9(3)
C(10)-P(2)-C(14)	103.8(5)
C(10)-P(2)-C(11)	104.8(4)
C(14)-P(2)-C(11)	111.6(4)
C(10)-P(2)-Re(1)	99.9(3)
C(14)-P(2)-Re(1)	117.2(3)
C(11)-P(2)-Re(1)	117.0(3)
C(1)-N(1)-C(9)	115.4(7)
C(1)-N(1)-Re(1)	116.2(6)
C(9)-N(1)-Re(1)	113.2(5)
C(1)-N(1)-H(111)	102(4)
C(9)-N(1)-H(111)	95(3)
Re(1)-N(1)-H(111)	112(6)
C(17)-N(2)-Re(1)	174.0(6)
N(1)-C(1)-C(2)	113.1(8)
C(1)-C(2)-P(1)	112.0(6)
C(5)-C(3)-C(4)	113.1(9)
C(5)-C(3)-P(1)	118.3(7)
C(4)-C(3)-P(1)	110.0(6)
C(7)-C(6)-C(8)	111.5(9)
C(7)-C(6)-P(1)	112.5(6)
C(8)-C(6)-P(1)	116.0(7)
C(10)-C(9)-N(1)	114.6(8)
C(9)-C(10)-P(2)	111.2(7)
C(12)-C(11)-C(13)	110.6(8)
C(12)-C(11)-P(2)	115.9(7)
C(13)-C(11)-P(2)	112.5(5)
C(15)-C(14)-C(16)	111.7(7)
C(15)-C(14)-P(2)	116.3(7)
C(16)-C(14)-P(2)	110.5(6)
N(2)-C(17)-C(18)	175.8(8)
C(23)-C(18)-C(19)	118.4(8)
C(23)-C(18)-C(17)	119.4(8)
C(19)-C(18)-C(17)	122.1(8)
C(18)-C(19)-C(20)	118.7(11)
C(21)-C(20)-C(19)	122.5(12)
C(20)-C(21)-C(22)	119.7(10)
C(21)-C(22)-C(23)	121.0(13)
C(18)-C(23)-C(22)	119.7(12)
C(25)-C(24)-C(29)	115.5(6)
C(25)-C(24)-B(1)	122.0(6)
C(29)-C(24)-B(1)	122.1(6)
C(26)-C(25)-C(24)	122.1(6)
C(27)-C(26)-C(25)	121.6(6)
C(27)-C(26)-C(30)	119.5(6)
C(25)-C(26)-C(30)	118.9(6)
C(26)-C(27)-C(28)	118.0(6)
C(27)-C(28)-C(29)	120.6(6)
C(27)-C(28)-C(31)	120.9(7)
C(29)-C(28)-C(31)	118.5(6)
C(28)-C(29)-C(24)	122.2(6)
F(1)-C(30)-F(2)	110.9(8)
F(1)-C(30)-F(3)	102.9(8)
F(2)-C(30)-F(3)	103.0(7)
F(1)-C(30)-C(26)	114.1(7)
F(2)-C(30)-C(26)	113.9(6)
F(3)-C(30)-C(26)	111.0(7)
F(6)-C(31)-F(5)	106.3(7)
F(6)-C(31)-F(4)	108.3(7)
F(5)-C(31)-F(4)	106.2(7)
F(6)-C(31)-C(28)	113.1(6)
F(5)-C(31)-C(28)	111.2(7)
F(4)-C(31)-C(28)	111.4(7)
C(37)-C(32)-C(33)	115.8(6)
C(37)-C(32)-B(1)	124.3(6)
C(33)-C(32)-B(1)	119.3(6)
C(34)-C(33)-C(32)	122.1(6)
C(35)-C(34)-C(33)	120.8(7)
C(35)-C(34)-C(38)	120.4(6)
C(33)-C(34)-C(38)	118.7(7)
C(34)-C(35)-C(36)	118.2(6)
C(35)-C(36)-C(37)	120.6(7)
C(35)-C(36)-C(39)	120.0(6)
C(37)-C(36)-C(39)	119.4(7)
C(32)-C(37)-C(36)	122.4(6)
F(8)-C(38)-F(9)	109.2(8)
F(8)-C(38)-F(7)	106.0(8)
F(9)-C(38)-F(7)	101.7(7)
F(8)-C(38)-C(34)	114.7(7)

F(9)-C(38)-C(34)	112.3(7)	F(20)-C(54)-F(21)	97.3(16)
F(7)-C(38)-C(34)	112.0(7)	F(21A)-C(54)-F(19A)	98(2)
F(11)-C(39)-F(12)	107.4(8)	F(20A)-C(54)-F(19A)	96(2)
F(11)-C(39)-F(10)	105.4(7)	F(21A)-C(54)-C(50)	116.6(16)
F(12)-C(39)-F(10)	105.5(6)	F(19)-C(54)-C(50)	117.1(12)
F(11)-C(39)-C(36)	112.9(6)	F(20A)-C(54)-C(50)	108.2(15)
F(12)-C(39)-C(36)	112.1(6)	F(20)-C(54)-C(50)	114.5(9)
F(10)-C(39)-C(36)	113.1(7)	F(21)-C(54)-C(50)	110.7(11)
C(45)-C(40)-C(41)	115.6(6)	F(19A)-C(54)-C(50)	103.4(14)
C(45)-C(40)-B(1)	121.4(6)	F(23)-C(55)-F(24)	109.2(11)
C(41)-C(40)-B(1)	122.7(6)	F(22A)-C(55)-F(23A)	112(2)
C(42)-C(41)-C(40)	121.8(6)	F(23)-C(55)-F(22)	106.0(9)
C(43)-C(42)-C(41)	121.1(7)	F(24)-C(55)-F(22)	105.1(9)
C(43)-C(42)-C(46)	121.5(7)	F(22A)-C(55)-C(52)	122.8(18)
C(41)-C(42)-C(46)	117.4(6)	F(23)-C(55)-C(52)	113.3(8)
C(42)-C(43)-C(44)	118.3(7)	F(24)-C(55)-C(52)	111.9(8)
C(43)-C(44)-C(45)	120.8(7)	F(23A)-C(55)-C(52)	112(2)
C(43)-C(44)-C(47)	119.6(8)	F(22)-C(55)-C(52)	110.8(9)
C(45)-C(44)-C(47)	119.4(8)	F(22A)-C(55)-F(24A)	100.4(18)
C(43)-C(44)-C(47A)	117(3)	F(23A)-C(55)-F(24A)	93.1(17)
C(45)-C(44)-C(47A)	118(3)	C(52)-C(55)-F(24A)	112.0(12)
C(44)-C(45)-C(40)	122.2(7)	C(92)-C(91)-C(90)	114(4)
F(15)-C(46)-F(13)	108.0(7)	C(91)-C(92)-C(93)	146(4)
F(15)-C(46)-F(14)	106.1(7)	C(94)-C(93)-C(92)	114(3)
F(13)-C(46)-F(14)	105.0(6)	C(32)-B(1)-C(40)	111.6(5)
F(15)-C(46)-C(42)	113.0(6)	C(32)-B(1)-C(24)	113.4(5)
F(13)-C(46)-C(42)	112.4(7)	C(40)-B(1)-C(24)	104.1(5)
F(14)-C(46)-C(42)	111.9(6)	C(32)-B(1)-C(48)	103.8(5)
C(53)-C(48)-C(49)	115.9(6)	C(40)-B(1)-C(48)	113.1(5)
C(53)-C(48)-B(1)	121.4(6)	C(24)-B(1)-C(48)	111.1(5)
C(49)-C(48)-B(1)	122.3(6)	F(17)-C(47)-F(16)	109.2(12)
C(50)-C(49)-C(48)	122.1(7)	F(17)-C(47)-F(18)	102.4(16)
C(51)-C(50)-C(49)	120.2(8)	F(16)-C(47)-F(18)	103.8(11)
C(51)-C(50)-C(54)	120.4(8)	F(17)-C(47)-C(44)	116.1(11)
C(49)-C(50)-C(54)	119.3(9)	F(16)-C(47)-C(44)	111.7(11)
C(50)-C(51)-C(52)	118.8(7)	F(18)-C(47)-C(44)	112.6(11)
C(51)-C(52)-C(53)	120.5(7)	F(17A)-C(47A)-F(16)	127(5)
C(51)-C(52)-C(55)	120.0(7)	F(17A)-C(47A)-F(18A)	114(6)
C(53)-C(52)-C(55)	119.5(8)	F(16)-C(47A)-F(18A)	95(5)
C(52)-C(53)-C(48)	122.4(7)	F(17A)-C(47A)-C(44)	115(6)
F(21A)-C(54)-F(20A)	128(2)	F(16)-C(47A)-C(44)	108(4)
F(19)-C(54)-F(20)	108.9(13)	F(18A)-C(47A)-C(44)	89(3)
F(19)-C(54)-F(21)	106.2(14)		

Symmetry transformations used to generate equivalent atoms:

*Table S4: Torsion angles [°] for **6**(BAr$^{F}_{24}$).*

C(9)-N(1)-C(1)-C(2)	-178.8(9)	C(3)-P(1)-C(2)-C(1)	-95.0(8)
Re(1)-N(1)-C(1)-C(2)	45.2(11)	Re(1)-P(1)-C(2)-C(1)	26.4(8)
N(1)-C(1)-C(2)-P(1)	-47.4(12)	C(6)-P(1)-C(3)-C(5)	54.0(10)
C(6)-P(1)-C(2)-C(1)	148.7(8)	C(2)-P(1)-C(3)-C(5)	-58.0(10)

Re(1)-P(1)-C(3)-C(5)	-166.9(9)
C(6)-P(1)-C(3)-C(4)	-78.2(8)
C(2)-P(1)-C(3)-C(4)	169.8(7)
Re(1)-P(1)-C(3)-C(4)	60.9(8)
C(2)-P(1)-C(6)-C(7)	-176.1(7)
C(3)-P(1)-C(6)-C(7)	70.6(7)
Re(1)-P(1)-C(6)-C(7)	-67.8(7)
C(2)-P(1)-C(6)-C(8)	53.8(9)
C(3)-P(1)-C(6)-C(8)	-59.5(9)
Re(1)-P(1)-C(6)-C(8)	162.2(7)
C(1)-N(1)-C(9)-C(10)	174.7(9)
Re(1)-N(1)-C(9)-C(10)	-48.0(11)
N(1)-C(9)-C(10)-P(2)	49.0(12)
C(14)-P(2)-C(10)-C(9)	-147.6(8)
C(11)-P(2)-C(10)-C(9)	95.2(8)
Re(1)-P(2)-C(10)-C(9)	-26.3(8)
C(10)-P(2)-C(11)-C(12)	61.2(8)
C(14)-P(2)-C(11)-C(12)	-50.6(8)
Re(1)-P(2)-C(11)-C(12)	170.6(6)
C(10)-P(2)-C(11)-C(13)	-170.2(7)
C(14)-P(2)-C(11)-C(13)	78.0(7)
Re(1)-P(2)-C(11)-C(13)	-60.8(7)
C(10)-P(2)-C(14)-C(15)	-43.5(8)
C(11)-P(2)-C(14)-C(15)	68.9(8)
Re(1)-P(2)-C(14)-C(15)	-152.5(6)
C(10)-P(2)-C(14)-C(16)	-172.2(6)
C(11)-P(2)-C(14)-C(16)	-59.8(7)
Re(1)-P(2)-C(14)-C(16)	78.9(6)
C(23)-C(18)-C(19)-C(20)	1.6(16)
C(17)-C(18)-C(19)-C(20)	176.9(9)
C(18)-C(19)-C(20)-C(21)	0.0(18)
C(19)-C(20)-C(21)-C(22)	-1(2)
C(20)-C(21)-C(22)-C(23)	1(2)
C(19)-C(18)-C(23)-C(22)	-2.1(18)
C(17)-C(18)-C(23)-C(22)	-177.5(11)
C(21)-C(22)-C(23)-C(18)	1(2)
C(29)-C(24)-C(25)-C(26)	0.4(10)
B(1)-C(24)-C(25)-C(26)	173.3(6)
C(24)-C(25)-C(26)-C(27)	-1.3(11)
C(24)-C(25)-C(26)-C(30)	179.8(7)
C(25)-C(26)-C(27)-C(28)	1.0(11)
C(30)-C(26)-C(27)-C(28)	179.9(7)
C(26)-C(27)-C(28)-C(29)	0.1(11)
C(26)-C(27)-C(28)-C(31)	178.4(7)
C(27)-C(28)-C(29)-C(24)	-1.0(11)
C(31)-C(28)-C(29)-C(24)	-179.3(7)
C(25)-C(24)-C(29)-C(28)	0.8(10)
B(1)-C(24)-C(29)-C(28)	-172.2(6)
C(27)-C(26)-C(30)-F(1)	7.6(13)
C(25)-C(26)-C(30)-F(1)	-173.4(9)
C(27)-C(26)-C(30)-F(2)	136.2(8)
C(25)-C(26)-C(30)-F(2)	-44.8(11)
C(27)-C(26)-C(30)-F(3)	-108.0(8)
C(25)-C(26)-C(30)-F(3)	70.9(9)
C(27)-C(28)-C(31)-F(6)	-110.5(8)
C(29)-C(28)-C(31)-F(6)	67.7(9)
C(27)-C(28)-C(31)-F(5)	129.9(8)
C(29)-C(28)-C(31)-F(5)	-51.9(10)
C(27)-C(28)-C(31)-F(4)	11.7(11)
C(29)-C(28)-C(31)-F(4)	-170.1(7)
C(37)-C(32)-C(33)-C(34)	-2.2(9)
B(1)-C(32)-C(33)-C(34)	-173.7(6)
C(32)-C(33)-C(34)-C(35)	0.5(11)
C(32)-C(33)-C(34)-C(38)	179.5(7)
C(33)-C(34)-C(35)-C(36)	1.3(11)
C(38)-C(34)-C(35)-C(36)	-177.6(7)
C(34)-C(35)-C(36)-C(37)	-1.4(10)
C(34)-C(35)-C(36)-C(39)	177.6(7)
C(33)-C(32)-C(37)-C(36)	2.1(9)
B(1)-C(32)-C(37)-C(36)	173.1(6)
C(35)-C(36)-C(37)-C(32)	-0.4(10)
C(39)-C(36)-C(37)-C(32)	-179.3(6)
C(35)-C(34)-C(38)-F(8)	-8.1(12)
C(33)-C(34)-C(38)-F(8)	172.9(8)
C(35)-C(34)-C(38)-F(9)	117.2(8)
C(33)-C(34)-C(38)-F(9)	-61.7(10)
C(35)-C(34)-C(38)-F(7)	-129.1(8)
C(33)-C(34)-C(38)-F(7)	52.0(10)
C(35)-C(36)-C(39)-F(11)	133.2(8)
C(37)-C(36)-C(39)-F(11)	-47.8(10)
C(35)-C(36)-C(39)-F(12)	-105.4(8)
C(37)-C(36)-C(39)-F(12)	73.6(9)
C(35)-C(36)-C(39)-F(10)	13.7(10)
C(37)-C(36)-C(39)-F(10)	-167.3(6)
C(45)-C(40)-C(41)-C(42)	-3.3(10)
B(1)-C(40)-C(41)-C(42)	-177.2(6)
C(40)-C(41)-C(42)-C(43)	2.4(11)
C(40)-C(41)-C(42)-C(46)	-177.0(6)
C(41)-C(42)-C(43)-C(44)	0.8(11)
C(46)-C(42)-C(43)-C(44)	-179.7(7)
C(42)-C(43)-C(44)-C(45)	-3.0(12)
C(42)-C(43)-C(44)-C(47)	171.3(10)
C(42)-C(43)-C(44)-C(47A)	-160(2)
C(43)-C(44)-C(45)-C(40)	2.1(13)
C(47)-C(44)-C(45)-C(40)	-172.2(10)
C(47A)-C(44)-C(45)-C(40)	158.7(19)
C(41)-C(40)-C(45)-C(44)	1.1(11)
B(1)-C(40)-C(45)-C(44)	175.0(7)
C(43)-C(42)-C(46)-F(15)	-111.7(9)
C(41)-C(42)-C(46)-F(15)	67.8(9)
C(43)-C(42)-C(46)-F(13)	10.8(11)
C(41)-C(42)-C(46)-F(13)	-169.7(7)
C(43)-C(42)-C(46)-F(14)	128.6(8)
C(41)-C(42)-C(46)-F(14)	-51.9(9)

C(53)-C(48)-C(49)-C(50)	0.5(10)	C(37)-C(32)-B(1)-C(40)	148.4(6)
B(1)-C(48)-C(49)-C(50)	172.7(7)	C(33)-C(32)-B(1)-C(40)	-40.9(8)
C(48)-C(49)-C(50)-C(51)	-1.5(12)	C(37)-C(32)-B(1)-C(24)	31.2(8)
C(48)-C(49)-C(50)-C(54)	179.7(8)	C(33)-C(32)-B(1)-C(24)	-158.1(6)
C(49)-C(50)-C(51)-C(52)	1.3(12)	C(37)-C(32)-B(1)-C(48)	-89.5(7)
C(54)-C(50)-C(51)-C(52)	-179.9(8)	C(33)-C(32)-B(1)-C(48)	81.2(7)
C(50)-C(51)-C(52)-C(53)	-0.3(12)	C(45)-C(40)-B(1)-C(32)	158.9(6)
C(50)-C(51)-C(52)-C(55)	-179.7(8)	C(41)-C(40)-B(1)-C(32)	-27.6(8)
C(51)-C(52)-C(53)-C(48)	-0.7(11)	C(45)-C(40)-B(1)-C(24)	-78.5(7)
C(55)-C(52)-C(53)-C(48)	178.7(7)	C(41)-C(40)-B(1)-C(24)	95.0(7)
C(49)-C(48)-C(53)-C(52)	0.6(10)	C(45)-C(40)-B(1)-C(48)	42.2(8)
B(1)-C(48)-C(53)-C(52)	-171.7(6)	C(41)-C(40)-B(1)-C(48)	-144.3(6)
C(51)-C(50)-C(54)-F(21A)	-141(3)	C(25)-C(24)-B(1)-C(32)	36.6(8)
C(49)-C(50)-C(54)-F(21A)	37(3)	C(29)-C(24)-B(1)-C(32)	-150.9(6)
C(51)-C(50)-C(54)-F(19)	15.8(19)	C(25)-C(24)-B(1)-C(40)	-84.8(7)
C(49)-C(50)-C(54)-F(19)	-165.5(15)	C(29)-C(24)-B(1)-C(40)	87.6(7)
C(51)-C(50)-C(54)-F(20A)	66(2)	C(25)-C(24)-B(1)-C(48)	153.1(6)
C(49)-C(50)-C(54)-F(20A)	-115(2)	C(29)-C(24)-B(1)-C(48)	-34.4(8)
C(51)-C(50)-C(54)-F(20)	145.2(12)	C(53)-C(48)-B(1)-C(32)	79.0(7)
C(49)-C(50)-C(54)-F(20)	-36.1(16)	C(49)-C(48)-B(1)-C(32)	-92.9(7)
C(51)-C(50)-C(54)-F(21)	-106.1(16)	C(53)-C(48)-B(1)-C(40)	-159.9(6)
C(49)-C(50)-C(54)-F(21)	72.7(17)	C(49)-C(48)-B(1)-C(40)	28.2(9)
C(51)-C(50)-C(54)-F(19A)	-35(2)	C(53)-C(48)-B(1)-C(24)	-43.3(8)
C(49)-C(50)-C(54)-F(19A)	143.9(19)	C(49)-C(48)-B(1)-C(24)	144.9(6)
C(51)-C(52)-C(55)-F(22A)	-28(3)	C(43)-C(44)-C(47)-F(17)	154.1(15)
C(53)-C(52)-C(55)-F(22A)	152(3)	C(45)-C(44)-C(47)-F(17)	-32(2)
C(51)-C(52)-C(55)-F(23)	143.2(11)	C(43)-C(44)-C(47)-F(16)	28.0(17)
C(53)-C(52)-C(55)-F(23)	-36.3(14)	C(45)-C(44)-C(47)-F(16)	-157.7(10)
C(51)-C(52)-C(55)-F(24)	-92.8(14)	C(43)-C(44)-C(47)-F(18)	-88.3(13)
C(53)-C(52)-C(55)-F(24)	87.8(13)	C(45)-C(44)-C(47)-F(18)	86.0(13)
C(51)-C(52)-C(55)-F(23A)	109(2)	C(43)-C(44)-C(47A)-F(17A)	120(5)
C(53)-C(52)-C(55)-F(23A)	-70(2)	C(45)-C(44)-C(47A)-F(17A)	-38(5)
C(51)-C(52)-C(55)-F(22)	24.2(13)	C(43)-C(44)-C(47A)-F(16)	-29(4)
C(53)-C(52)-C(55)-F(22)	-155.3(10)	C(45)-C(44)-C(47A)-F(16)	173(2)
C(51)-C(52)-C(55)-F(24A)	-147.9(19)	C(43)-C(44)-C(47A)-F(18A)	-124(3)
C(53)-C(52)-C(55)-F(24A)	33(2)	C(45)-C(44)-C(47A)-F(18A)	78(4)
C(90)-C(91)-C(92)-C(93)	-173(5)		
C(91)-C(92)-C(93)-C(94)	-100(7)		

Symmetry transformations used to generate equivalent atoms:

5.2 Crystal Structure of *trans*-**5**(BAr^F_{24})

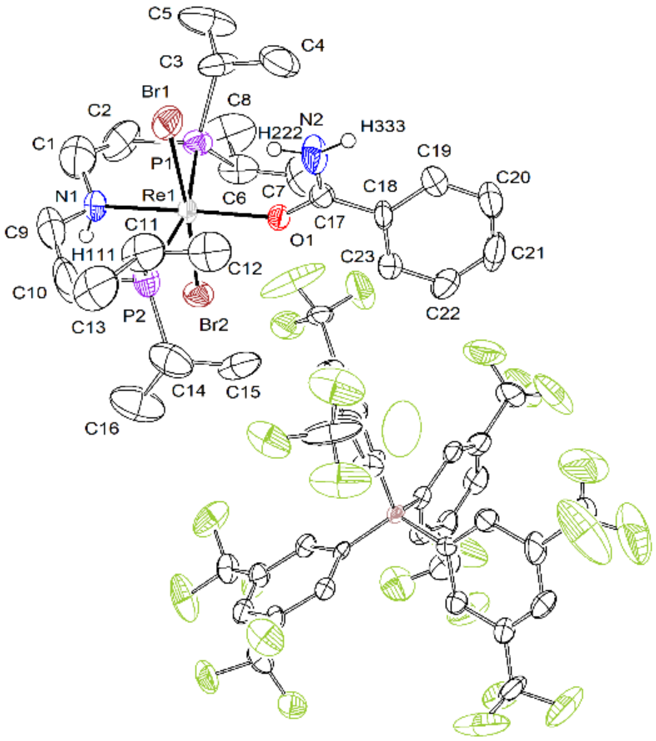

*Figure S64: Thermal ellipsoid plot of trans-**5**(BAr^F_{24}) with the anisotropic displacement parameters drawn at the 50% probability level. The asymmetric unit contains one complex molecule and one disordered counter ion. The complex molecule and the disordered counter ion were refined with population of 0.62(8) on the main domain using some restraints and constraints (FREE, ISOR, SADI). The reflections 1 1 0, 0 -1 1, 1 0 1, 0 1 1, -3 3 0, -2 0 1, -2,7 1, 1 -1 2 and 0 -2 3 are removed from the refinement using OMIT commands. The N-H atoms were found from the residual density map and isotropically refined using DFIX. The SQUEEZE routine has been applied.[14]*

*Table S5: Crystal data and structure refinement for trans-**5**(BAr^F_{24}).*

Identification code	mo_lzj_240725_ko_0m_a_sq	
Empirical formula	$C_{55}H_{56}BBr_2F_{24}N_2OP_2Re$	
Formula weight	1635.78	
Temperature	100(2) K	
Wavelength	0.71073 Å	
Crystal system	Triclinic	
Space group	P-1	
Unit cell dimensions	a = 12.7866(3) Å	α= 89.857(2)°
	b = 15.7003(4) Å	β= 81.9030(10)°
	c = 16.7092(4) Å	γ = 84.6880(10)°
Volume	3306.54(14) Å^3	
Z	2	
Density (calculated)	1.643 Mg/m^3	
Absorption coefficient	3.200 mm^{-1}	
F(000)	1608	
Crystal size	0.162 x 0.106 x 0.087 mm^3	

Theta range for data collection	2.371 to 25.025°.
Index ranges	–15<=h<=15, -18<=k<=18, -19<=l<=19
Reflections collected	98492
Independent reflections	11655 [R(int) = 0.0715]
Completeness to theta = 25.025°	99.8 %
Max. and min. transmission	0.77 and 0.70
Refinement method	Full-matrix least-squares on F^2
Data / restraints / parameters	11655 / 35 / 821
Goodness-of-fit on F^2	1.112
Final R indices [I>2sigma(I)]	R1 = 0.0768, wR2 = 0.1561
R indices (all data)	R1 = 0.0998, wR2 = 0.1732
Largest diff. peak and hole	2.157 and –2.043 eÅ^{-3}

*Table S6: Bond lengths [Å] and angles [°] for trans-**5**(BAr$^F{}_{24}$).*

Re(1)-O(1)	2.076(6)	Re(1)-N(1)	2.145(9)
Re(1)-P(1)	2.447(3)	Re(1)-P(2)	2.462(3)
Re(1)-Br(2)	2.4877(12)	Re(1)-Br(1)	2.4918(13)
P(1)-C(2)	1.850(14)	P(1)-C(3)	1.860(12)
P(1)-C(6)	1.876(13)	P(2)-C(10)	1.832(14)
P(2)-C(14)	1.833(16)	P(2)-C(11)	1.846(16)
F(3)-C(30)	1.298(13)	F(4)-C(31)	1.247(16)
F(6)-C(31)	1.199(16)	F(7)-C(38)	1.328(13)
F(8)-C(38)	1.349(12)	F(9)-C(38)	1.330(13)
F(10)-C(39)	1.325(13)	F(11)-C(39)	1.342(13)
F(12)-C(39)	1.317(12)	F(13)-C(46)	1.343(16)
F(14)-C(46)	1.292(14)	F(15)-C(46)	1.317(15)
F(16)-C(47)	1.258(16)	F(17)-C(47)	1.292(15)
F(18)-C(47)	1.322(14)	F(19)-C(54)	1.291(15)
F(20)-C(54)	1.315(15)	F(21)-C(54)	1.328(14)
F(22)-C(55)	1.37(2)	F(23)-C(55)	1.22(2)
F(24)-C(55)	1.25(2)	O(1)-C(17)	1.241(11)
N(1)-C(1)	1.39(2)	N(1)-C(9)	1.507(19)
N(1)-H(111)	0.91(2)	N(2)-C(17)	1.333(14)
N(2)-H(222)	0.90(13)	N(2)-H(333)	0.94(12)
C(1)-C(2)	1.53(2)	C(11)-C(12)	1.53(2)
C(11)-C(13)	1.541(18)	C(14)-C(16)	1.50(2)
C(14)-C(15)	1.51(2)	C(17)-C(18)	1.480(14)
C(18)-C(23)	1.363(16)	C(18)-C(19)	1.386(15)
C(19)-C(20)	1.372(18)	C(20)-C(21)	1.35(2)
C(21)-C(22)	1.357(19)	C(22)-C(23)	1.377(16)
C(3)-C(5)	1.511(18)	C(3)-C(4)	1.521(19)
C(24)-C(29)	1.398(13)	C(24)-C(25)	1.406(13)
C(24)-B(1)	1.654(13)	C(25)-C(26)	1.381(14)
C(26)-C(27)	1.391(15)	C(26)-C(30)	1.502(14)
C(27)-C(28)	1.403(15)	C(28)-C(29)	1.381(15)
C(28)-C(31)	1.533(17)	C(30)-F(1)	1.278(14)
C(30)-F(2)	1.290(14)	C(32)-C(33)	1.409(13)

C(32)-C(37)	1.412(13)	C(32)-B(1)	1.614(13)
C(33)-C(34)	1.392(13)	C(34)-C(35)	1.386(14)
C(34)-C(38)	1.505(14)	C(35)-C(36)	1.364(15)
C(36)-C(37)	1.374(14)	C(36)-C(39)	1.488(13)
C(40)-C(45)	1.391(13)	C(40)-C(41)	1.400(13)
C(40)-B(1)	1.645(14)	C(41)-C(42)	1.391(13)
C(42)-C(43)	1.361(16)	C(42)-C(46)	1.514(16)
C(43)-C(44)	1.372(16)	C(44)-C(45)	1.398(13)
C(44)-C(47)	1.508(15)	C(48)-C(53)	1.387(14)
C(48)-C(49)	1.414(13)	C(48)-B(1)	1.636(13)
C(49)-C(50)	1.393(13)	C(50)-C(51)	1.368(16)
C(50)-C(54)	1.484(16)	C(51)-C(52)	1.389(18)
C(52)-C(53)	1.404(15)	C(52)-C(55)	1.493(19)
C(6)-C(7)	1.518(19)	C(6)-C(8)	1.535(19)
C(9)-C(10)	1.37(2)	C(31)-F(5A)	1.45(3)
C(31)-F(5)	1.71(3)		
O(1)-Re(1)-N(1)	176.0(4)	O(1)-Re(1)-P(1)	95.7(2)
N(1)-Re(1)-P(1)	82.0(3)	O(1)-Re(1)-P(2)	100.1(2)
N(1)-Re(1)-P(2)	82.2(3)	P(1)-Re(1)-P(2)	164.18(11)
O(1)-Re(1)-Br(2)	88.48(18)	N(1)-Re(1)-Br(2)	88.1(3)
P(1)-Re(1)-Br(2)	89.48(8)	P(2)-Re(1)-Br(2)	90.90(9)
O(1)-Re(1)-Br(1)	95.84(18)	N(1)-Re(1)-Br(1)	87.5(3)
P(1)-Re(1)-Br(1)	90.13(8)	P(2)-Re(1)-Br(1)	88.30(9)
Br(2)-Re(1)-Br(1)	175.68(4)	C(2)-P(1)-C(3)	106.2(7)
C(2)-P(1)-C(6)	104.9(7)	C(3)-P(1)-C(6)	109.5(7)
C(2)-P(1)-Re(1)	98.9(5)	C(3)-P(1)-Re(1)	117.6(4)
C(6)-P(1)-Re(1)	117.5(4)	C(10)-P(2)-C(14)	107.7(8)
C(10)-P(2)-C(11)	105.5(8)	C(14)-P(2)-C(11)	102.4(7)
C(10)-P(2)-Re(1)	98.1(5)	C(14)-P(2)-Re(1)	123.7(5)
C(11)-P(2)-Re(1)	117.7(5)	C(17)-O(1)-Re(1)	144.0(7)
C(1)-N(1)-C(9)	116.9(12)	C(1)-N(1)-Re(1)	117.8(9)
C(9)-N(1)-Re(1)	113.5(9)	C(1)-N(1)-H(111)	100.9(18)
C(9)-N(1)-H(111)	92.1(15)	Re(1)-N(1)-H(111)	111.4(18)
C(17)-N(2)-H(222)	113(8)	C(17)-N(2)-H(333)	121(3)
H(222)-N(2)-H(333)	124(9)	N(1)-C(1)-C(2)	114.3(14)
C(1)-C(2)-P(1)	111.3(10)	C(12)-C(11)-C(13)	110.1(14)
C(12)-C(11)-P(2)	111.3(9)	C(13)-C(11)-P(2)	115.4(13)
C(16)-C(14)-C(15)	111.3(14)	C(16)-C(14)-P(2)	115.1(13)
C(15)-C(14)-P(2)	111.0(9)	O(1)-C(17)-N(2)	122.5(10)
O(1)-C(17)-C(18)	118.1(9)	N(2)-C(17)-C(18)	119.4(9)
C(23)-C(18)-C(19)	119.2(10)	C(23)-C(18)-C(17)	119.0(9)
C(19)-C(18)-C(17)	121.7(10)	C(20)-C(19)-C(18)	120.6(13)
C(21)-C(20)-C(19)	118.7(12)	C(20)-C(21)-C(22)	121.9(12)
C(21)-C(22)-C(23)	119.6(13)	C(5)-C(3)-C(4)	114.8(14)
C(5)-C(3)-P(1)	117.2(10)	C(4)-C(3)-P(1)	110.5(9)
C(18)-C(23)-C(22)	120.0(12)	C(29)-C(24)-C(25)	116.7(8)
C(29)-C(24)-B(1)	118.4(8)	C(25)-C(24)-B(1)	124.5(8)
C(26)-C(25)-C(24)	121.9(9)	C(25)-C(26)-C(27)	121.5(9)
C(25)-C(26)-C(30)	119.4(9)	C(27)-C(26)-C(30)	119.1(9)
C(26)-C(27)-C(28)	116.6(9)	C(29)-C(28)-C(27)	122.4(10)
C(29)-C(28)-C(31)	119.3(10)	C(27)-C(28)-C(31)	118.1(10)
C(28)-C(29)-C(24)	121.0(10)	F(1)-C(30)-F(2)	108.8(12)

F(1)-C(30)-F(3)	105.0(11)	F(2)-C(30)-F(3)	104.0(10)
F(1)-C(30)-C(26)	113.0(9)	F(2)-C(30)-C(26)	113.0(10)
F(3)-C(30)-C(26)	112.3(9)	C(33)-C(32)-C(37)	114.7(8)
C(33)-C(32)-B(1)	121.9(8)	C(37)-C(32)-B(1)	123.2(8)
C(34)-C(33)-C(32)	121.8(8)	C(35)-C(34)-C(33)	121.0(9)
C(35)-C(34)-C(38)	121.5(9)	C(33)-C(34)-C(38)	117.5(9)
C(36)-C(35)-C(34)	118.3(9)	C(35)-C(36)-C(37)	121.3(9)
C(35)-C(36)-C(39)	120.4(9)	C(37)-C(36)-C(39)	118.3(10)
C(36)-C(37)-C(32)	122.8(9)	F(7)-C(38)-F(9)	105.8(10)
F(7)-C(38)-F(8)	106.9(9)	F(9)-C(38)-F(8)	106.5(9)
F(7)-C(38)-C(34)	112.4(9)	F(9)-C(38)-C(34)	112.9(9)
F(8)-C(38)-C(34)	111.8(9)	F(12)-C(39)-F(10)	106.7(9)
F(12)-C(39)-F(11)	105.0(9)	F(10)-C(39)-F(11)	104.5(9)
F(12)-C(39)-C(36)	113.6(9)	F(10)-C(39)-C(36)	114.1(9)
F(11)-C(39)-C(36)	112.0(9)	C(45)-C(40)-C(41)	116.0(9)
C(45)-C(40)-B(1)	122.2(8)	C(41)-C(40)-B(1)	120.8(8)
C(42)-C(41)-C(40)	121.6(9)	C(43)-C(42)-C(41)	121.7(10)
C(43)-C(42)-C(46)	121.6(10)	C(41)-C(42)-C(46)	116.6(10)
C(42)-C(43)-C(44)	117.7(9)	C(43)-C(44)-C(45)	121.7(10)
C(43)-C(44)-C(47)	120.2(10)	C(45)-C(44)-C(47)	118.1(10)
C(40)-C(45)-C(44)	121.2(10)	F(14)-C(46)-F(15)	109.8(12)
F(14)-C(46)-F(13)	104.7(11)	F(15)-C(46)-F(13)	99.0(12)
F(14)-C(46)-C(42)	113.8(11)	F(15)-C(46)-C(42)	114.0(10)
F(13)-C(46)-C(42)	114.2(10)	F(16)-C(47)-F(17)	109.0(14)
F(16)-C(47)-F(18)	107.4(11)	F(17)-C(47)-F(18)	101.7(11)
F(16)-C(47)-C(44)	113.8(10)	F(17)-C(47)-C(44)	112.4(10)
F(18)-C(47)-C(44)	111.7(11)	C(53)-C(48)-C(49)	116.3(9)
C(53)-C(48)-B(1)	123.2(8)	C(49)-C(48)-B(1)	120.0(8)
C(50)-C(49)-C(48)	122.6(9)	C(51)-C(50)-C(49)	119.2(10)
C(51)-C(50)-C(54)	121.1(10)	C(49)-C(50)-C(54)	119.7(10)
C(50)-C(51)-C(52)	120.4(10)	C(51)-C(52)-C(53)	119.8(11)
C(51)-C(52)-C(55)	120.3(11)	C(53)-C(52)-C(55)	119.8(12)
C(48)-C(53)-C(52)	121.7(10)	F(19)-C(54)-F(20)	106.1(12)
F(19)-C(54)-F(21)	106.3(11)	F(20)-C(54)-F(21)	105.5(11)
F(19)-C(54)-C(50)	113.0(10)	F(20)-C(54)-C(50)	112.7(10)
F(21)-C(54)-C(50)	112.6(11)	F(23)-C(55)-F(24)	114.2(19)
F(23)-C(55)-F(22)	100.2(18)	F(24)-C(55)-F(22)	99.3(14)
F(23)-C(55)-C(52)	117.3(13)	F(24)-C(55)-C(52)	112.5(17)
F(22)-C(55)-C(52)	110.9(15)	C(32)-B(1)-C(48)	112.5(7)
C(32)-B(1)-C(40)	112.7(7)	C(48)-B(1)-C(40)	103.0(7)
C(32)-B(1)-C(24)	103.9(7)	C(48)-B(1)-C(24)	111.2(7)
C(40)-B(1)-C(24)	113.8(8)	C(7)-C(6)-C(8)	113.6(14)
C(7)-C(6)-P(1)	111.2(9)	C(8)-C(6)-P(1)	114.1(11)
C(10)-C(9)-N(1)	117.2(13)	C(9)-C(10)-P(2)	113.2(11)
F(6)-C(31)-F(4)	123.0(15)	F(6)-C(31)-F(5A)	90.8(16)
F(4)-C(31)-F(5A)	97.5(13)	F(6)-C(31)-C(28)	115.7(11)
F(4)-C(31)-C(28)	113.8(13)	F(5A)-C(31)-C(28)	110.0(15)
F(6)-C(31)-F(5)	75.8(16)	F(4)-C(31)-F(5)	71.4(16)
C(28)-C(31)-F(5)	97.5(15)		

Symmetry transformations used to generate equivalent atoms:

*Table S7: Torsion angles [°] for trans-**5**(BAr$^{F}{}_{24}$).*

C(9)-N(1)-C(1)-C(2)	-175.9(14)	Re(1)-N(1)-C(1)-C(2)	44(2)
N(1)-C(1)-C(2)-P(1)	-43.7(19)	C(3)-P(1)-C(2)-C(1)	-99.2(13)
C(6)-P(1)-C(2)-C(1)	144.8(12)	Re(1)-P(1)-C(2)-C(1)	23.1(13)
C(10)-P(2)-C(11)-C(12)	178.2(11)	C(14)-P(2)-C(11)-C(12)	65.6(12)
Re(1)-P(2)-C(11)-C(12)	-73.6(11)	C(10)-P(2)-C(11)-C(13)	51.8(14)
C(14)-P(2)-C(11)-C(13)	-60.8(13)	Re(1)-P(2)-C(11)-C(13)	160.0(10)
C(10)-P(2)-C(14)-C(16)	25.8(15)	C(11)-P(2)-C(14)-C(16)	136.8(13)
Re(1)-P(2)-C(14)-C(16)	-87.2(13)	C(10)-P(2)-C(14)-C(15)	153.3(11)
C(11)-P(2)-C(14)-C(15)	-95.7(12)	Re(1)-P(2)-C(14)-C(15)	40.3(13)
Re(1)-O(1)-C(17)-N(2)	10(2)	Re(1)-O(1)-C(17)-C(18)	-169.8(8)
O(1)-C(17)-C(18)-C(23)	-18.1(16)	N(2)-C(17)-C(18)-C(23)	162.2(12)
O(1)-C(17)-C(18)-C(19)	160.9(11)	N(2)-C(17)-C(18)-C(19)	-18.8(17)
C(23)-C(18)-C(19)-C(20)	-1(2)	C(17)-C(18)-C(19)-C(20)	-179.7(12)
C(18)-C(19)-C(20)-C(21)	0(2)	C(19)-C(20)-C(21)-C(22)	1(2)
C(20)-C(21)-C(22)-C(23)	-2(2)	C(2)-P(1)-C(3)-C(5)	-53.2(16)
C(6)-P(1)-C(3)-C(5)	59.7(15)	Re(1)-P(1)-C(3)-C(5)	-162.7(13)
C(2)-P(1)-C(3)-C(4)	172.8(12)	C(6)-P(1)-C(3)-C(4)	-74.4(12)
Re(1)-P(1)-C(3)-C(4)	63.2(13)	C(19)-C(18)-C(23)-C(22)	0.7(19)
C(17)-C(18)-C(23)-C(22)	179.7(12)	C(21)-C(22)-C(23)-C(18)	0(2)
C(29)-C(24)-C(25)-C(26)	2.3(14)	B(1)-C(24)-C(25)-C(26)	174.5(8)
C(24)-C(25)-C(26)-C(27)	-1.6(15)	C(24)-C(25)-C(26)-C(30)	179.0(9)
C(25)-C(26)-C(27)-C(28)	-0.4(16)	C(30)-C(26)-C(27)-C(28)	179.0(10)
C(26)-C(27)-C(28)-C(29)	1.5(18)	C(26)-C(27)-C(28)-C(31)	-173.5(14)
C(27)-C(28)-C(29)-C(24)	-0.6(18)	C(31)-C(28)-C(29)-C(24)	174.3(14)
C(25)-C(24)-C(29)-C(28)	-1.2(15)	B(1)-C(24)-C(29)-C(28)	-173.9(10)
C(25)-C(26)-C(30)-F(1)	-65.5(15)	C(27)-C(26)-C(30)-F(1)	115.1(13)
C(25)-C(26)-C(30)-F(2)	170.3(11)	C(27)-C(26)-C(30)-F(2)	-9.1(16)
C(25)-C(26)-C(30)-F(3)	53.0(14)	C(27)-C(26)-C(30)-F(3)	-126.4(11)
C(37)-C(32)-C(33)-C(34)	-0.2(12)	B(1)-C(32)-C(33)-C(34)	175.0(8)
C(32)-C(33)-C(34)-C(35)	1.9(14)	C(32)-C(33)-C(34)-C(38)	-179.7(9)
C(33)-C(34)-C(35)-C(36)	-1.8(14)	C(38)-C(34)-C(35)-C(36)	179.9(10)
C(34)-C(35)-C(36)-C(37)	-0.1(15)	C(34)-C(35)-C(36)-C(39)	177.9(9)
C(35)-C(36)-C(37)-C(32)	1.9(16)	C(39)-C(36)-C(37)-C(32)	-176.1(9)
C(33)-C(32)-C(37)-C(36)	-1.7(13)	B(1)-C(32)-C(37)-C(36)	-176.8(9)
C(35)-C(34)-C(38)-F(7)	-133.7(10)	C(33)-C(34)-C(38)-F(7)	48.0(13)
C(35)-C(34)-C(38)-F(9)	106.6(11)	C(33)-C(34)-C(38)-F(9)	-71.7(12)
C(35)-C(34)-C(38)-F(8)	-13.4(14)	C(33)-C(34)-C(38)-F(8)	168.2(9)
C(35)-C(36)-C(39)-F(12)	-120.4(11)	C(37)-C(36)-C(39)-F(12)	57.7(14)
C(35)-C(36)-C(39)-F(10)	2.3(15)	C(37)-C(36)-C(39)-F(10)	-179.6(10)
C(35)-C(36)-C(39)-F(11)	120.8(11)	C(37)-C(36)-C(39)-F(11)	-61.1(13)
C(45)-C(40)-C(41)-C(42)	-2.7(14)	B(1)-C(40)-C(41)-C(42)	-171.4(9)
C(40)-C(41)-C(42)-C(43)	1.7(16)	C(40)-C(41)-C(42)-C(46)	178.6(9)
C(41)-C(42)-C(43)-C(44)	-0.4(16)	C(46)-C(42)-C(43)-C(44)	-177.2(10)
C(42)-C(43)-C(44)-C(45)	0.4(16)	C(42)-C(43)-C(44)-C(47)	178.8(10)
C(41)-C(40)-C(45)-C(44)	2.7(13)	B(1)-C(40)-C(45)-C(44)	171.3(8)
C(43)-C(44)-C(45)-C(40)	-1.7(15)	C(47)-C(44)-C(45)-C(40)	179.9(10)
C(43)-C(42)-C(46)-F(14)	-16.3(18)	C(41)-C(42)-C(46)-F(14)	166.8(12)
C(43)-C(42)-C(46)-F(15)	110.7(14)	C(41)-C(42)-C(46)-F(15)	-66.2(15)
C(43)-C(42)-C(46)-F(13)	-136.5(12)	C(41)-C(42)-C(46)-F(13)	46.6(15)

C(43)-C(44)-C(47)-F(16)	-106.8(15)	C(45)-C(44)-C(47)-F(16)	71.6(15)
C(43)-C(44)-C(47)-F(17)	128.6(14)	C(45)-C(44)-C(47)-F(17)	-53.0(17)
C(43)-C(44)-C(47)-F(18)	15.1(16)	C(45)-C(44)-C(47)-F(18)	-166.5(10)
C(53)-C(48)-C(49)-C(50)	-2.2(15)	B(1)-C(48)-C(49)-C(50)	169.9(9)
C(48)-C(49)-C(50)-C(51)	3.1(16)	C(48)-C(49)-C(50)-C(54)	-178.8(10)
C(49)-C(50)-C(51)-C(52)	-2.8(18)	C(54)-C(50)-C(51)-C(52)	179.1(12)
C(50)-C(51)-C(52)-C(53)	2(2)	C(50)-C(51)-C(52)-C(55)	-176.5(14)
C(49)-C(48)-C(53)-C(52)	1.0(16)	B(1)-C(48)-C(53)-C(52)	-170.8(11)
C(51)-C(52)-C(53)-C(48)	-0.9(19)	C(55)-C(52)-C(53)-C(48)	177.4(14)
C(51)-C(50)-C(54)-F(19)	104.3(14)	C(49)-C(50)-C(54)-F(19)	-73.7(16)
C(51)-C(50)-C(54)-F(20)	-135.4(12)	C(49)-C(50)-C(54)-F(20)	46.6(15)
C(51)-C(50)-C(54)-F(21)	-16.2(18)	C(49)-C(50)-C(54)-F(21)	165.8(11)
C(51)-C(52)-C(55)-F(23)	151.6(18)	C(53)-C(52)-C(55)-F(23)	-27(3)
C(51)-C(52)-C(55)-F(24)	-73(2)	C(53)-C(52)-C(55)-F(24)	108.9(18)
C(51)-C(52)-C(55)-F(22)	37(2)	C(53)-C(52)-C(55)-F(22)	-141.0(15)
C(33)-C(32)-B(1)-C(48)	36.9(11)	C(37)-C(32)-B(1)-C(48)	-148.4(8)
C(33)-C(32)-B(1)-C(40)	152.8(8)	C(37)-C(32)-B(1)-C(40)	-32.5(11)
C(33)-C(32)-B(1)-C(24)	-83.6(9)	C(37)-C(32)-B(1)-C(24)	91.2(10)
C(53)-C(48)-B(1)-C(32)	-148.2(9)	C(49)-C(48)-B(1)-C(32)	40.3(12)
C(53)-C(48)-B(1)-C(40)	90.2(11)	C(49)-C(48)-B(1)-C(40)	-81.3(10)
C(53)-C(48)-B(1)-C(24)	-32.1(13)	C(49)-C(48)-B(1)-C(24)	156.4(8)
C(45)-C(40)-B(1)-C(32)	154.7(8)	C(41)-C(40)-B(1)-C(32)	-37.3(11)
C(45)-C(40)-B(1)-C(48)	-83.8(10)	C(41)-C(40)-B(1)-C(48)	84.2(10)
C(45)-C(40)-B(1)-C(24)	36.7(11)	C(41)-C(40)-B(1)-C(24)	-155.3(8)
C(29)-C(24)-B(1)-C(32)	76.6(10)	C(25)-C(24)-B(1)-C(32)	-95.4(10)
C(29)-C(24)-B(1)-C(48)	-44.7(11)	C(25)-C(24)-B(1)-C(48)	143.3(9)
C(29)-C(24)-B(1)-C(40)	-160.4(8)	C(25)-C(24)-B(1)-C(40)	27.5(12)
C(2)-P(1)-C(6)-C(7)	-176.9(10)	C(3)-P(1)-C(6)-C(7)	69.4(11)
Re(1)-P(1)-C(6)-C(7)	-68.2(11)	C(2)-P(1)-C(6)-C(8)	53.0(15)
C(3)-P(1)-C(6)-C(8)	-60.6(14)	Re(1)-P(1)-C(6)-C(8)	161.7(12)
C(1)-N(1)-C(9)-C(10)	176.2(16)	Re(1)-N(1)-C(9)-C(10)	-41.5(18)
N(1)-C(9)-C(10)-P(2)	46(2)	C(14)-P(2)-C(10)-C(9)	-156.2(14)
C(11)-P(2)-C(10)-C(9)	95.0(15)	Re(1)-P(2)-C(10)-C(9)	-26.9(16)
C(29)-C(28)-C(31)-F(6)	-3(3)	C(27)-C(28)-C(31)-F(6)	172.4(17)
C(29)-C(28)-C(31)-F(4)	148.1(15)	C(27)-C(28)-C(31)-F(4)	-37(2)
C(29)-C(28)-C(31)-F(5A)	-103.7(14)	C(27)-C(28)-C(31)-F(5A)	71.4(16)
C(29)-C(28)-C(31)-F(5)	75.1(17)	C(27)-C(28)-C(31)-F(5)	-109.7(16)

Symmetry transformations used to generate equivalent atoms:

6. Computational Details

All geometry optimizations and harmonic frequency calculations were performed using the Gaussian16 program package.[15] The B3LYP[16,17,18] density functional combined with the def2-SVP[19] basis set and the Grimme D3[20] dispersion correction was used and zero-point vibrational energies and thermal contributions to Gibbs free energies at 298.15K were obtained at this level of density functional theory. Optimized structures were characterized as minima by eigenvalue analysis of the computed Hessians. Single point calculations on optimized geometries were performed with the TURBOMOLE program package (Version 7.9) employing the scLH22t[21] local hybrid functional in combination with the def2-TZVPP[19] basis set and the D4[22] dispersion correction.[23,24] The total energies obtained at this level were corrected to free energies by adding the respective increments obtained at the B3LYP-D3/def2-SVP level of DFT along with a volume-based correction of entropic contributions in solution as suggested by Ariai and Gellrich.[25] Further, free-energy-of-solvation contributions were added to these energies as obtained with the openCOSMO-RS[26] solvation model implemented in ORCA 6.0.1[27,28] with ortho-difluorobenzene (DFB) as solvent, unless specified otherwise. As we were unable to achieve SCF convergence for the Ir-dimer **9** with the openCOSMO-RS default method BP86/def2-TZVPD, the PBE0[29,30] hybrid functional was used instead in combination the def2-TZVPP basis set throughout to avoid diffuse basis functions and critically small HOMO-LUMO gaps. Differences in BDFEs between the two methods obtained for all other cases were insignificant (see Table S3). Benchmark single point calculations were performed using explicitly correlated coupled cluster theory, CCSD(T)-F12b[31,32] with Molpro (version 2020.2).[33] Here, the one-particle space was described with the cc-pVDZ-F12[34] basis for all non-metal atoms and the aug-cc-pVDZ-PP[35] basis for iridium; the corresponding JKfit triple-zeta auxiliary fit basis sets,[36,37,38,39] the MP2fit sets for density fitting and the OptRI/JKfit sets for construction of the complementary auxiliary basis set (OptRI keyword for non-metal atoms, JKfit for iridium) were used as implemented in Molpro. TD-DFT calculations for iridium porphyrin complexes were performed using the TURBOMOLE program package (Version 7.9) employing the M06L[40] density functional combined with the D4 dispersion correction and the def2-TZVPP basis set. Solvation effects were accounted for implicitly using COSMO[41] with 1,2-difluorobenezene as the solvent. Additional relativistic spin-orbit TD-DFT calculations for **10⁺** were performed in AMS 2024.102 using the X2C approach together with the M06L functional and the TZP basis set. Solvation effects were accounted for implicitly using COSMO[41] with 1,2-difluorobenezene as the solvent. Comparison with the (scalar relativistic) ECP TD-DFT computations on **10⁺** shows only moderate blue-shifts by about 20 nm for the two absorptions relevant for the photoproduct assignments as discussed in the main text. As also the character of the computed NTOs remains consistent (Schemes S16 and S17 below), we refrained from further SOC-TD-DFT calculations for other species due to enormous computational costs.

In addition to the pure DFT energies for the full molecular systems, improved single point energies were computed with a two-layer ONIOM(QM:QM)[42,43,44] approach, in which high-level (HL) CCSD(T)-F12b energies for H-truncated model systems are combined with the low-level (LL) COSMO-RS(DFB)-scLH22t energies for the H-truncated model and the full (‘real’) molecular system. Hence, while the coupled cluster calculations on the model system were performed under gas-phase conditions, the DFT calculations on the real system include implicit solvation effects. The H-truncated model was constructed by replacing certain parts of the complexes with hydrogen atoms and reoptimizing only the new R–H bond lengths while keeping all other degrees of freedom frozen. The individual truncation pattern for each complex is illustrated below. The ONIOM energy is given as:

$$E_{\mathrm{ONIOM(HL:LL)}} = E_{\mathrm{LL}}^{\mathrm{real}} - E_{\mathrm{LL}}^{\mathrm{model}} + E_{\mathrm{HL}}^{\mathrm{model}}$$

Spin-orbit stabilizations of relevant rhenium and iridium complexes were calculated in ORCA at the DFT ground-state geometries using state-averaged CASSCF/NEVPT2[45,46,47] calculations followed the quasi-degenerate perturbation theory (QDPT) treatment via a spin-orbit mean field (SOMF)[48]. For the CASSCF calculations the ZORA[49] approximation was used combined with the ZORA-def2-TZVP[50] basis set for all atoms except for iridium and rhenium for which the SARC-ZORA-TZVP basis set was used. RIJK was used for fitting of Coulomb and exchange integrals employing the def2/JK[51] auxiliary basis set. The chosen active space and state averaging is shown below for the respective complex. As the influence of the Ph-substituents in the TPP ligand on BDFEs was found to be small the calculations of spin orbit stabilizations were performed on model complexes containing a parent porphyrin ligand to decrease the computational demand.

Pictures of molecular structures were generated with the ChemDraw program. All energies given are relative total energies (Δ*E*) or free energies at 298.15 K and 1 atm (ΔG^{298}) in kcal mol^{-1}.

For benchmarking purpose, we investigated the Ir–H BDFE of the parent porphyrin complex and the N–H BDFEs of benzamide and the O–H BDFE of TEMPOH (Table S1). Overall, the DFT values are in good agreement with the CCSD(T)-F12b results and only minor errors relative to the experimental data are observed.

Table S8: BDFE benchmark, Gibbs free energies in kcal mol^{-1}.

	CCSD(T)-F12b[a]	scLH22t[a]	Exp.
(Porphyrin)Ir–H	73.7	72.9	–
Ph–C(O)–NH_2 (N–H)	107.7	108.9	102.6 ± 2 (DMSO)[52,53]
Ph–C(O)–NH$^\bullet$ (N–H)	96.5	95.8	–
TEMPO–H	68.5	65.5	65.5 ± 1 (THF)[54]
H–H	101.2	101.0	104 (THF)[55]

[a] The BDFEs include implicit solvation effects and entropy corrections as described above. DFB was used for the Ir–H BDFE. For better comparison with the experimental values, DMSO was used for benzamide and THF for TEMPOH and H_2 in the COSMO-RS calculations.

Table S9: Comparison of ONIOM(CC-F12:scLH22t), scLH22t and spin-orbit corrected scLH22t energies for the full molecular complexes, energies in kcal mol^{-1}.

	ONIOM(CC:DFT[a])	scLH22t[a]	scLH22t + ΔE_{SOC}[b]
(TPP)Ir–H (**8**), BDFE	74.2	73.0	66.1
(TPP)Ir–Ir(TPP) (**9**), BDFE	–	44.1	30.3
**9** + H_2 → 2 x **8**	–	–1.0	–1.0
(PNP)Re–O=C(Ph)(NH_2)$^+$ (**5**$^+$), N–H BDFE	57.8	58.6	61.8
(PNP)Re–N=C(Ph)(OH)$^+$ (**4**$^+$), O–H BDFE	43.8	48.1	53.9
$\Delta E_{T\text{-}S}$ **3**$^+$, adiabatic	21.3	19.0	n.d.
$\Delta E_{T\text{-}S}$ **3**$^+$, vertical	27.6	24.4	n.d.
Hydrogenation of **3**$^+$	–0.4	–5.7	–14.7
H–H, BDFE	101.2[c]	101.0	n.d.
(TPP)Ir–H$^+$ (**8**$^+$), BDFE	–	67.0	60.6
(PNP)Re–N(H)–C(O)(Ph), N–H BDFE	–	49.2	53.3
(PNP)Re–N(H)–C(O)(Ph), trans-cis Isomerization to **4**	–	–8.1	–8.7

[a] HL method: CCSD(T)-F12b/vdz-F12, LL method: scLH22t-D4/def2-TZVPP; scLH22t calculations for the full molecular system include implicit COSMO-RS(DFB) solvation effects as described above. [b] Spin orbit contributions from SA-CASSCF/NEVPT2/QDPT computations, see above. [c] Calculated using only CCSD(T)-F12b energies.

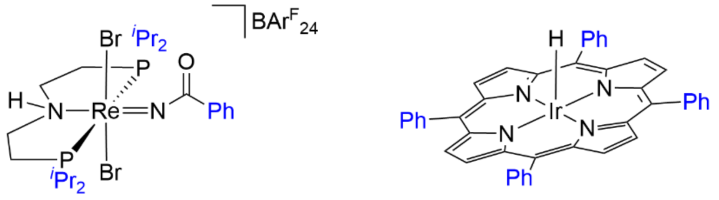

*Scheme S1: Truncation scheme employed to construct the molecular models of the iridium and rhenium complexes used in ONIOM calculations exemplified for complexes **3**⁺ (left) and **8** (right); substituents (blue) are replaced with hydrogen in the model systems.*

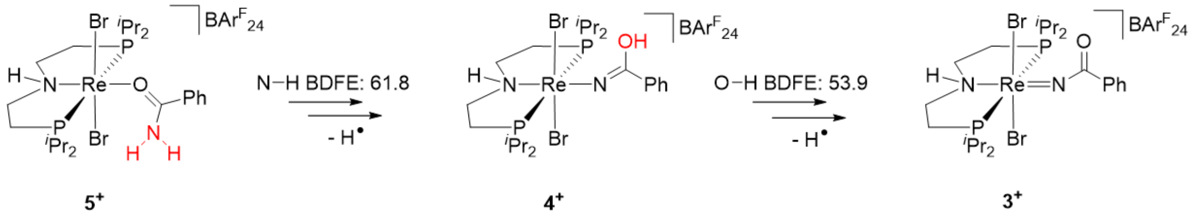

*Scheme S2: BDFEs for bonds relevant in the hydrogenation of **3**⁺ (in red), scLH22t+SOC energies in kcal mol⁻¹.*

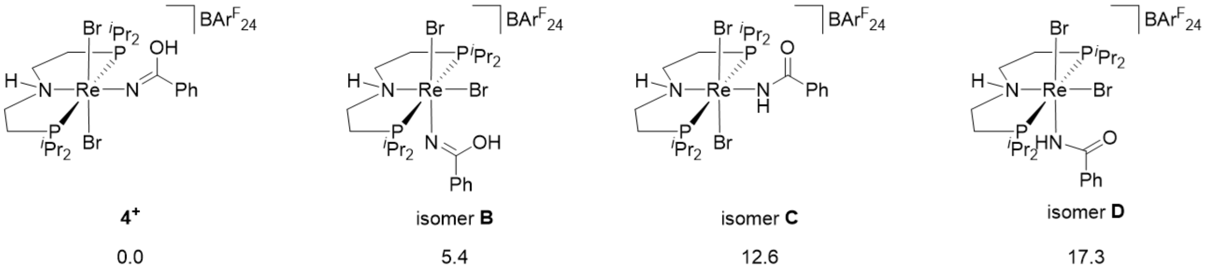

*Scheme S3: Relative stability of possible isomers and conformers of the rhenium complex **4**⁺ after the first HAT (scLH22t-D4/def2-TZVPP gas phase data), energies in kcal mol⁻¹.*

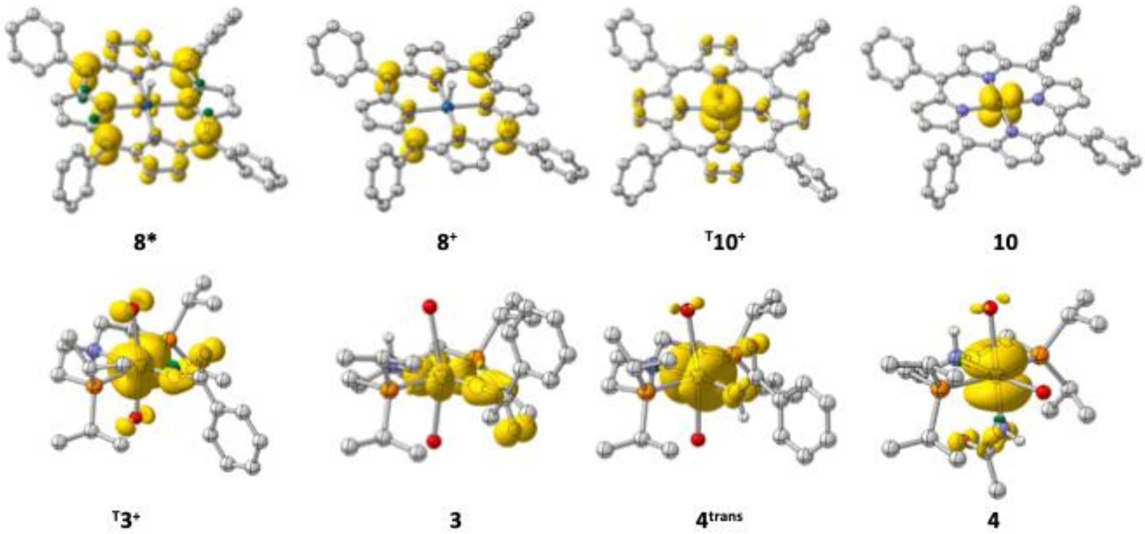

Figure S65:Spin density plots of relevant open-shell species, (scLH22t-D4/def2-TZVPP gas phase data), isovalue at 0.005 a_0^{-3}.

Table S10: Comparison of the free energies of solvation (DFB as solvent) obtained with openCOSMO-RS using the default and the modified DFT method, energies in kcal mol⁻¹.

	BP86/def2-TZVPD	PBE0/def2-TZVPP
$H^{\bullet}$	–0.06	–0.05
H_2	–0.18	–0.19
(TPP)Ir–H (**8**)	–32.92	–34.04
(TPP)$Ir^{\bullet}$ (**10**)	–32.38	–33.45
(TPP)Ir–Ir(TPP) (**9**)	–	–57.99
(PNP)Re=NC(O)$(Ph)^+$ (**3⁺**)	–55.20	–56.01
(PNP)Re–O=C(Ph)$(NH_2)^+$ (**5⁺**)	–54.32	–54.93
(PNP)Re–N=C(Ph)$(OH)^+$ (**4⁺**)	–54.04	–54.73

As can be seen from Table S10, the differences between $G_{\mathrm{solv}}^{298.15}$ values obtained with the two methods are moderate, and the resulting differences in BDFEs are below 1 kcal mol^{-1}.

Table S11: Total energies of the real systems and H-truncated model systems, ONIOM extrapolated total energies, and corrections to free energies ($G_{corr}^{298.15}$), free energies of solvation ($G_{solv}^{298.15}$), entropic corrections according to Ariai and Gellrich (S_{Solv}), and spin-orbit stabilization for the full system used for ONIOM calculations (energies in hartrees, S_{Solv} in cal mol–1 K–1, kcal mol–1).

	scLH22t		CC-F12	ONIOM				CASSCF/ NEVPT2 /QDPT
Species	$E_{\mathrm{LL}}^{\mathrm{real}}$	$E_{\mathrm{LL}}^{\mathrm{model}}$	$E_{\mathrm{HL}}^{\mathrm{model}}$	$E_{\mathrm{ONIOM(HL:LL)}}^{real}$	$G_{\mathrm{corr}}^{298.15}$	$G_{\mathrm{solv}}^{298.15}$	S_{Solv}	ΔE_{SOC}
H•	-0.495619	–	-0.499809	–	-0.010654	-0.000082	-15.726	
H_2	-1.164606	–	-1.173228	–	-0.001575	-0.000305	-16.306	
**8**	-2015.588839	-1092.309151	-1091.641253	-2014.291153	0.536406	-0.054251	-30.261	
(TPP)Ir• (**10**)	-2014.965058	-1091.685969	-1091.012064	-2014.920940	0.526914	-0.053299	-30.364	–6.9
**9**	-4030.045397	–	–	–	1.098164	-0.092407	-32.315	
**3**	-6992.969554	-6290.847900	-1974.459278	-2676.580932	0.553487	-0.089250	-26.844	
(PNP)Re–O=C(Ph)(NH_2)$^+$ (**5**$^+$)	-6994.161348	-6292.064920	-1975.676554	-2677.772982	0.575760	-0.087538	-26.901	–3.4
(PNP)Re–N=C(Ph)(OH)$^+$ (**4**$^+$)	-6993.557910	-6291.447423	-1975.056087	-2677.166574	0.564251	-0.087221	-26.871	–5.8
**3**$^+$, triplet	-6992.939234	-6290.820930	-1974.428629	-2676.546934	0.551549	-0.092721	–	
**3**$^+$, triplet (vertical)	-6992.930662	-6290.814993	-1974.421220	-2676.536889	–	–	–	
**4**$^+$-Isomer **B**	-6993.549328	–	–	–	–	–	–	
**4**$^+$-Isomer **C**	-6993.537907	–	–	–	–	–	–	–5.8
**4**$^+$-Isomer **D**	-6993.530321	–	–	–	–	–	–	
**8**$^+$	-2015.350778	–	–	–	0.535879	-0.093184	-30.258	–2.3
**10**$^+$	-2014.738418	–	–	–	0.527315	-0.091403	-30.365	–8.7
**4**	-6993.759697	–	–	–	0.560726	-0.045079	-26.926	–8.0
**4**, Br trans	-6993.746851	–	–	–	0.563288	-0.047670	-27.021	–7.4
**trans5**$^+$	-6994.161348	–	–	–	0.575760	-0.087538	-26.901	–9.0

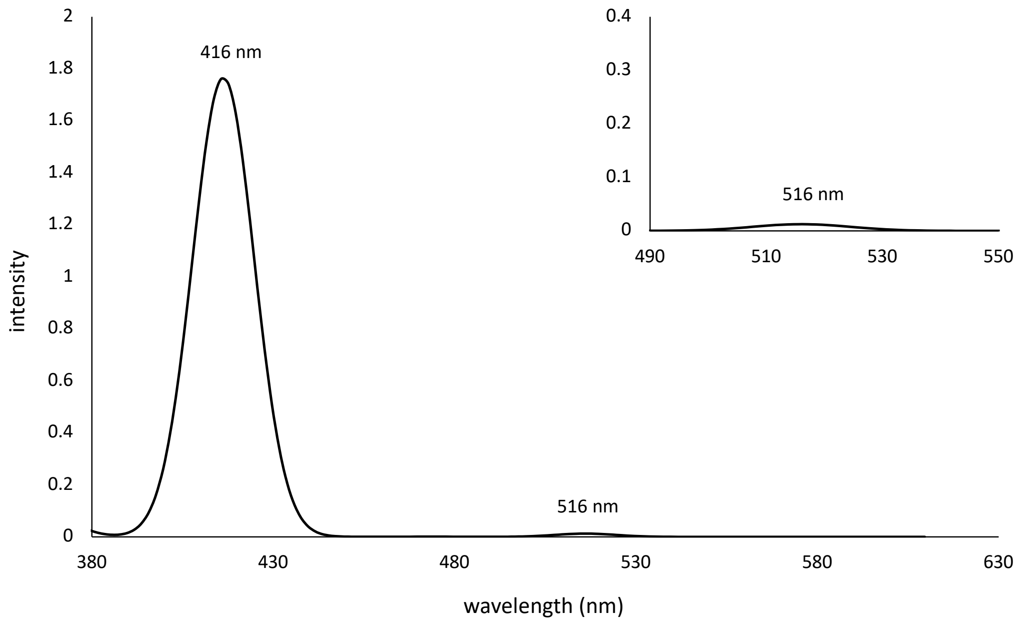

*Figure S66: TD-DFT spectrum of **8**, inset: enlarged Q-band region (COSMO-M06L-D4/def2-TZVPP data).*

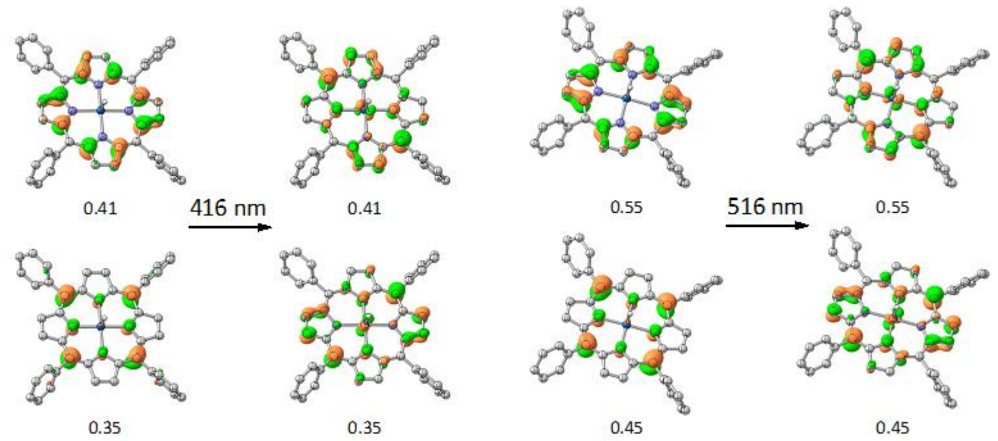

*Scheme S4: Natural transition orbitals contributing to the calculated TD-DFT absorption bands of **8** (COSMO-M06L-D4/def2-TZVPP data), isovalue at 0.05 $a_0^{-3/2}$.*

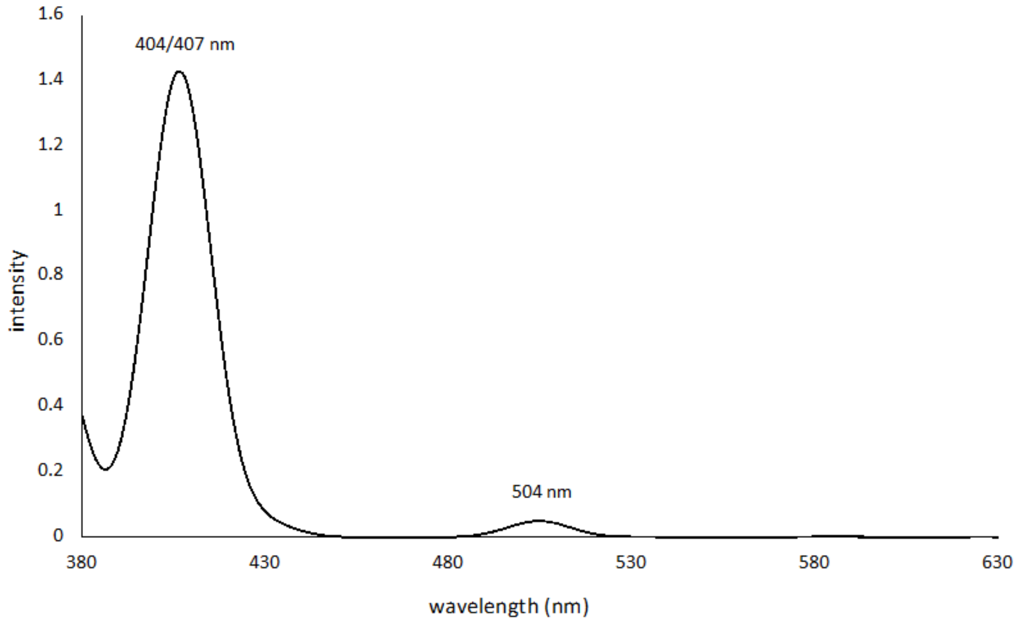

*Figure S67: TD-DFT spectrum of (TPP)Ir• (**10**), (COSMO-M06L-D4/def2-TZVPPdata).*

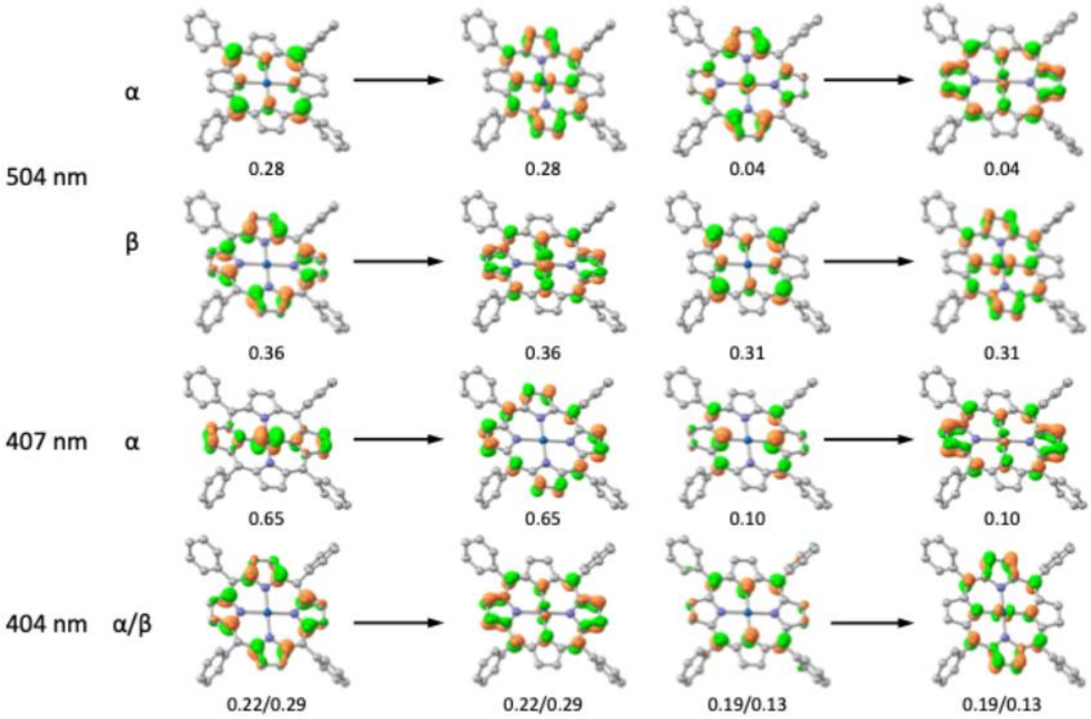

*Scheme S5: Natural transition orbitals contributing to the calculated TD-DFT absorption bands of (TPP)Ir• (**10**) (COSMO-M06L-D4/def2-TZVPP data), isovalue at 0.05 $a_0^{-3/2}$.*

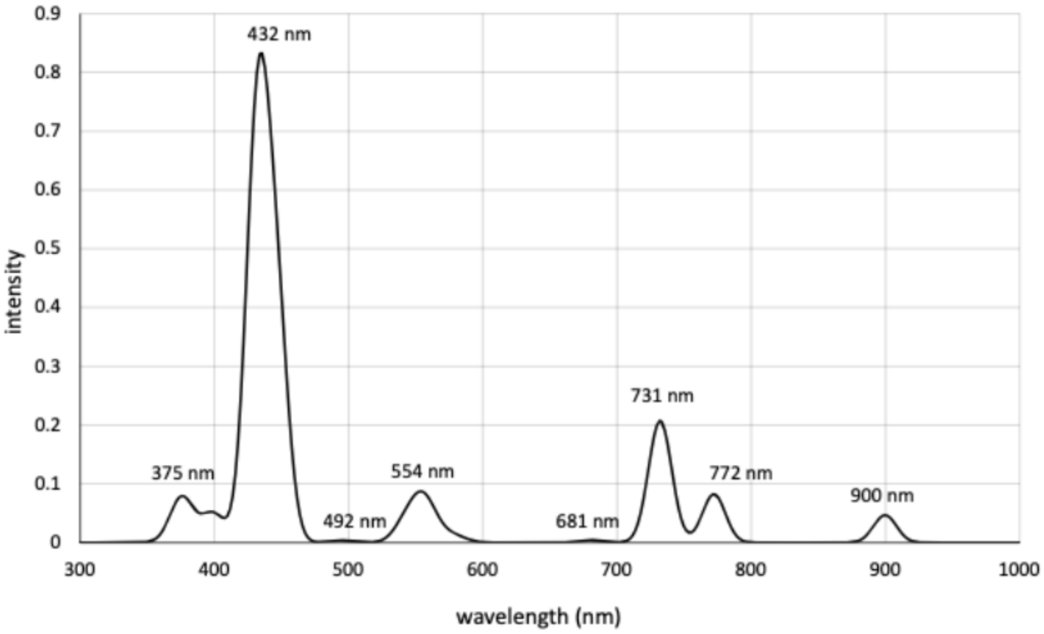

*Figure S68: TD-DFT spectrum of triplet **8***, (COSMO-M06L-D4/def2-TZVPP data).*

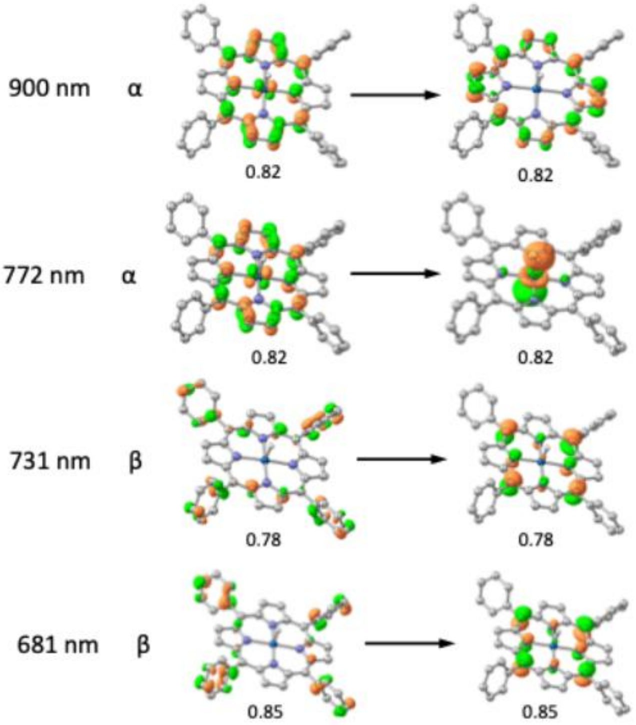

*Scheme S6: Natural transition orbitals contributing to the calculated TD-DFT absorption bands of triplet **8***, (COSMO-M06L-D4/def2-TZVPP data), isovalue at 0.05* $a_0^{-3/2}$.

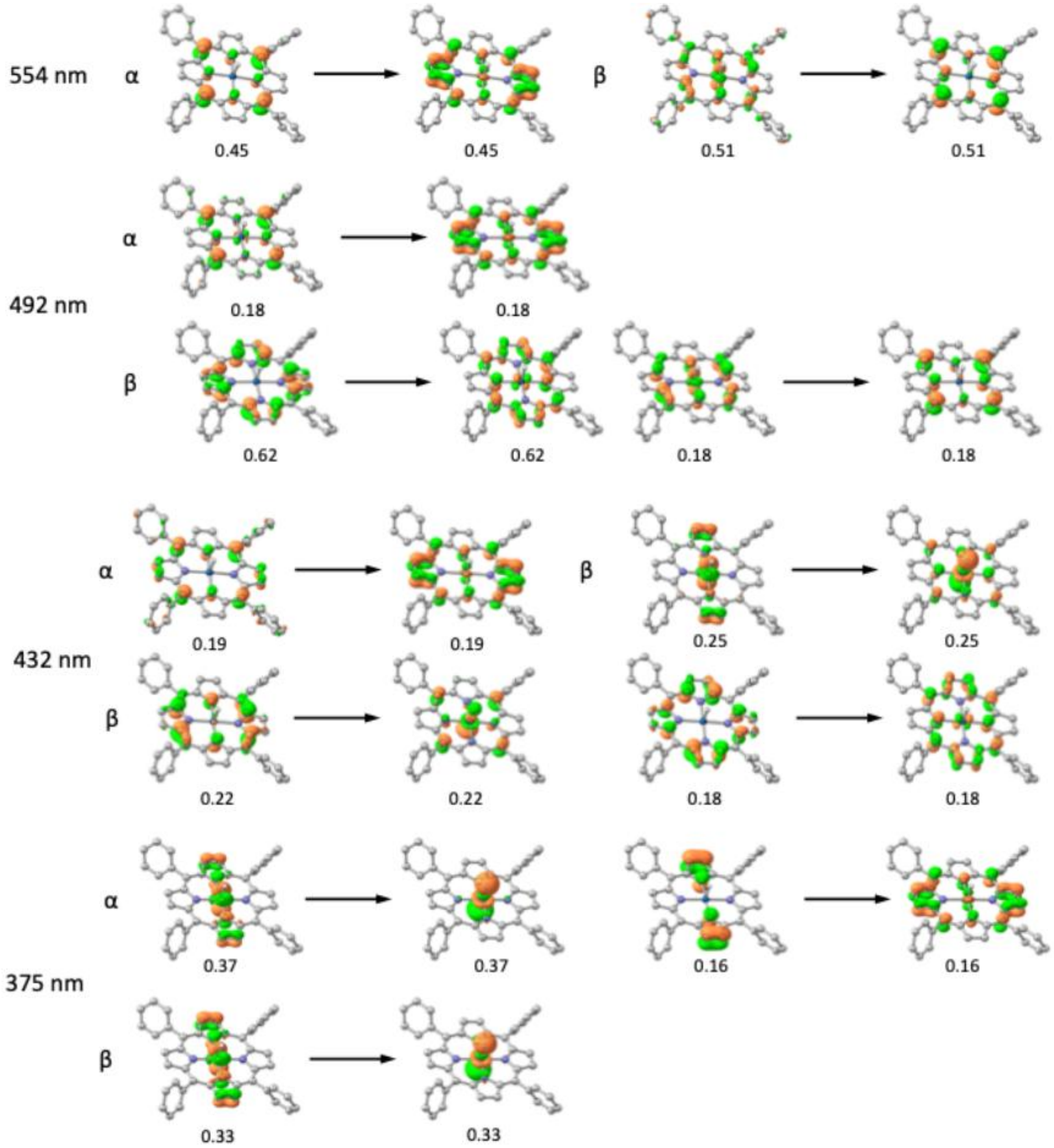

*Scheme S7: Natural transition orbitals contributing to the calculated TD-DFT absorption bands of triplet **8***, (COSMO-M06L-D4/def2-TZVPP data), isovalue at 0.05 $a_0^{-3/2}$.*

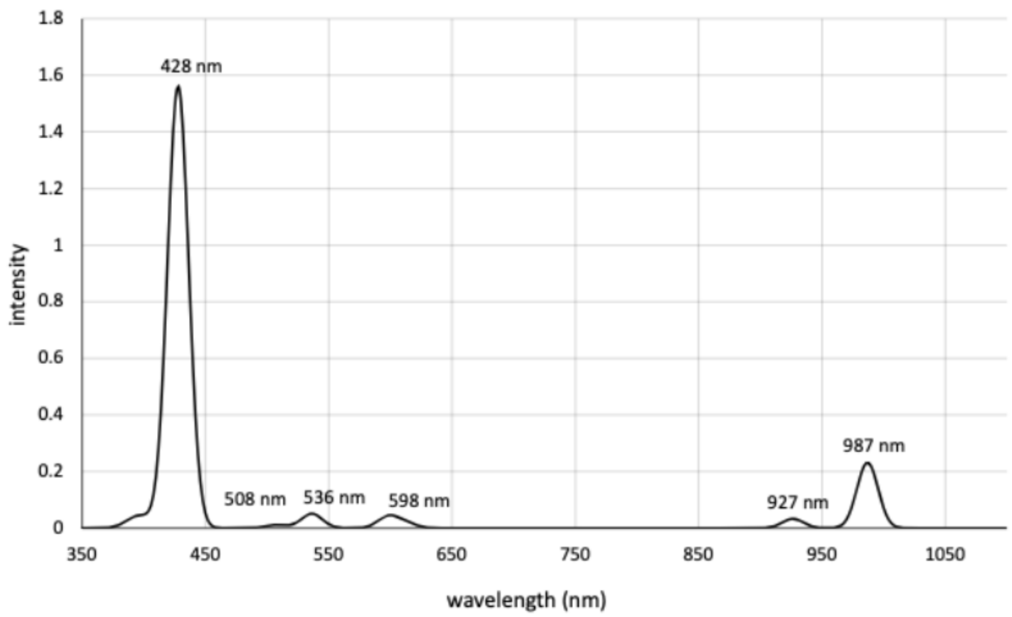

*Figure S69: TD-DFT spectrum of cationic **8**⁺, (COSMO-M06L-D4/def2-TZVPP data).*

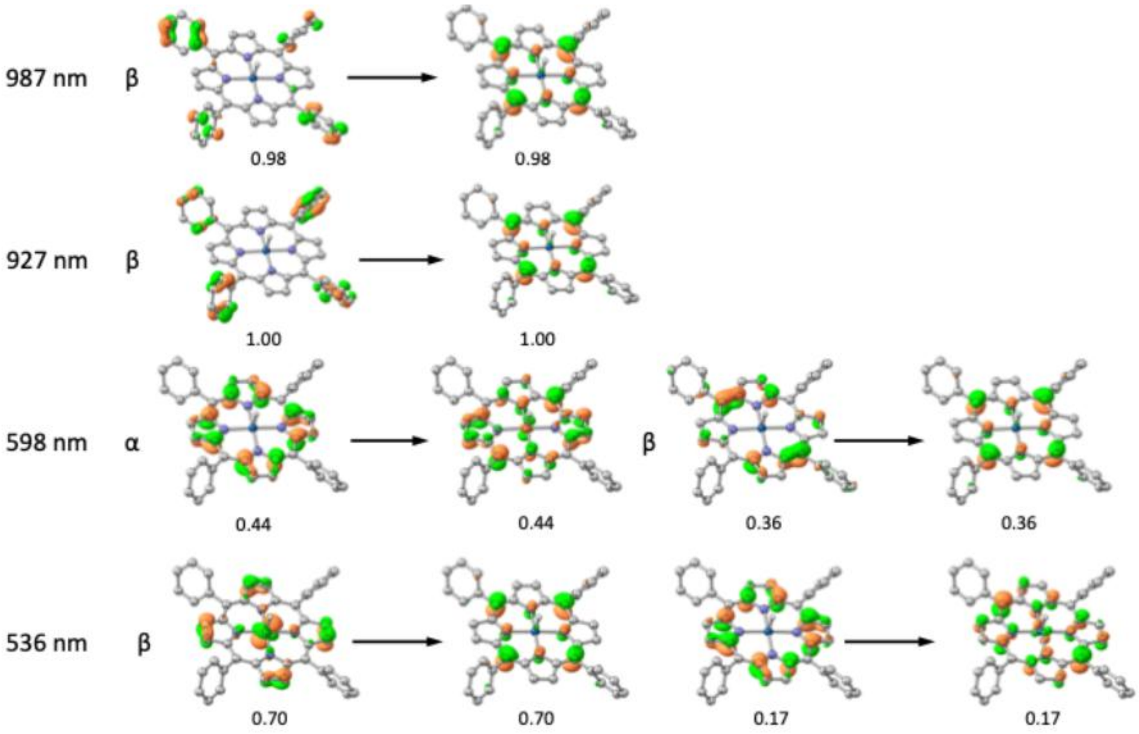

*Scheme S8: Natural transition orbitals contributing to the calculated TD-DFT absorption bands of cationic **8**⁺, (COSMO-M06L-D4/def2-TZVPP data), isovalue at 0.05* $a_0^{-3/2}$.

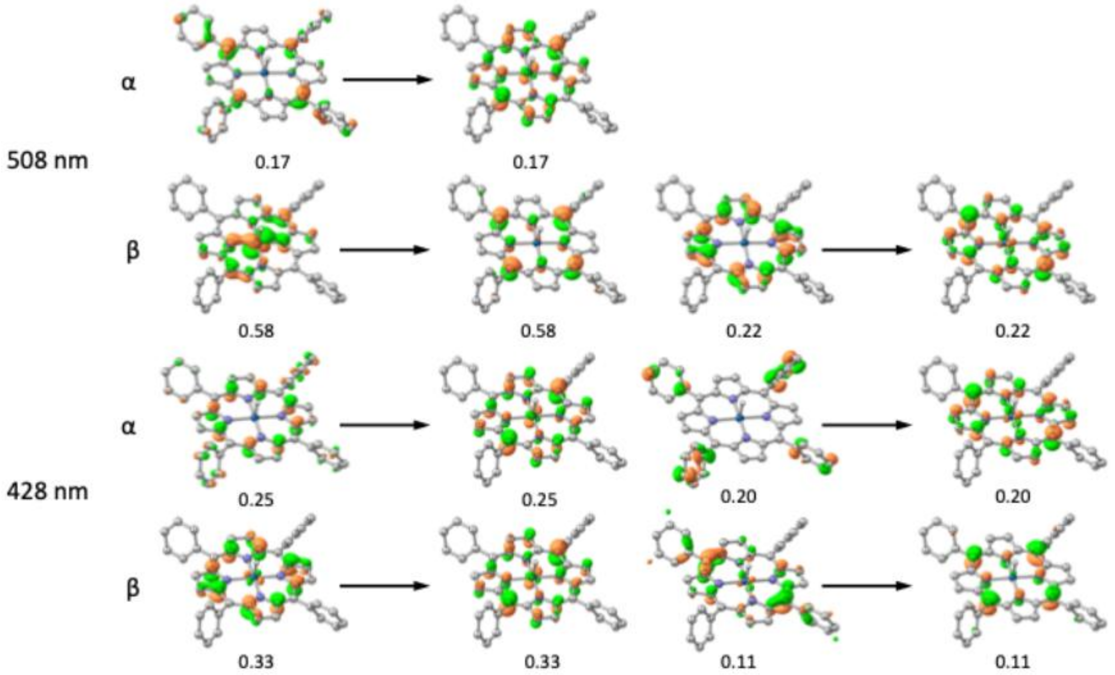

*Scheme S9(continued): Natural transition orbitals contributing to the calculated TD-DFT absorption bands of cationic **8⁺**, (COSMO-M06L-D4/def2-TZVPP data), isovalue at 0.05 $a_0^{-3/2}$.*

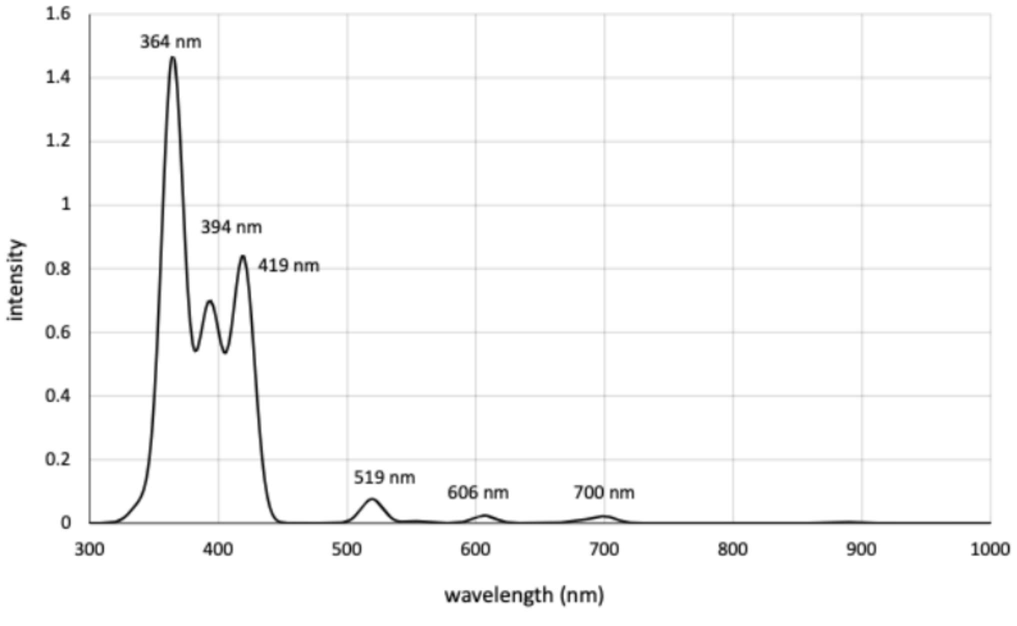

*Figure S70: TD-DFT spectrum of triplet, cationic (TPP)Ir⁺ (**10⁺**), (COSMO-M06L-D4/def2-TZVPP data).*

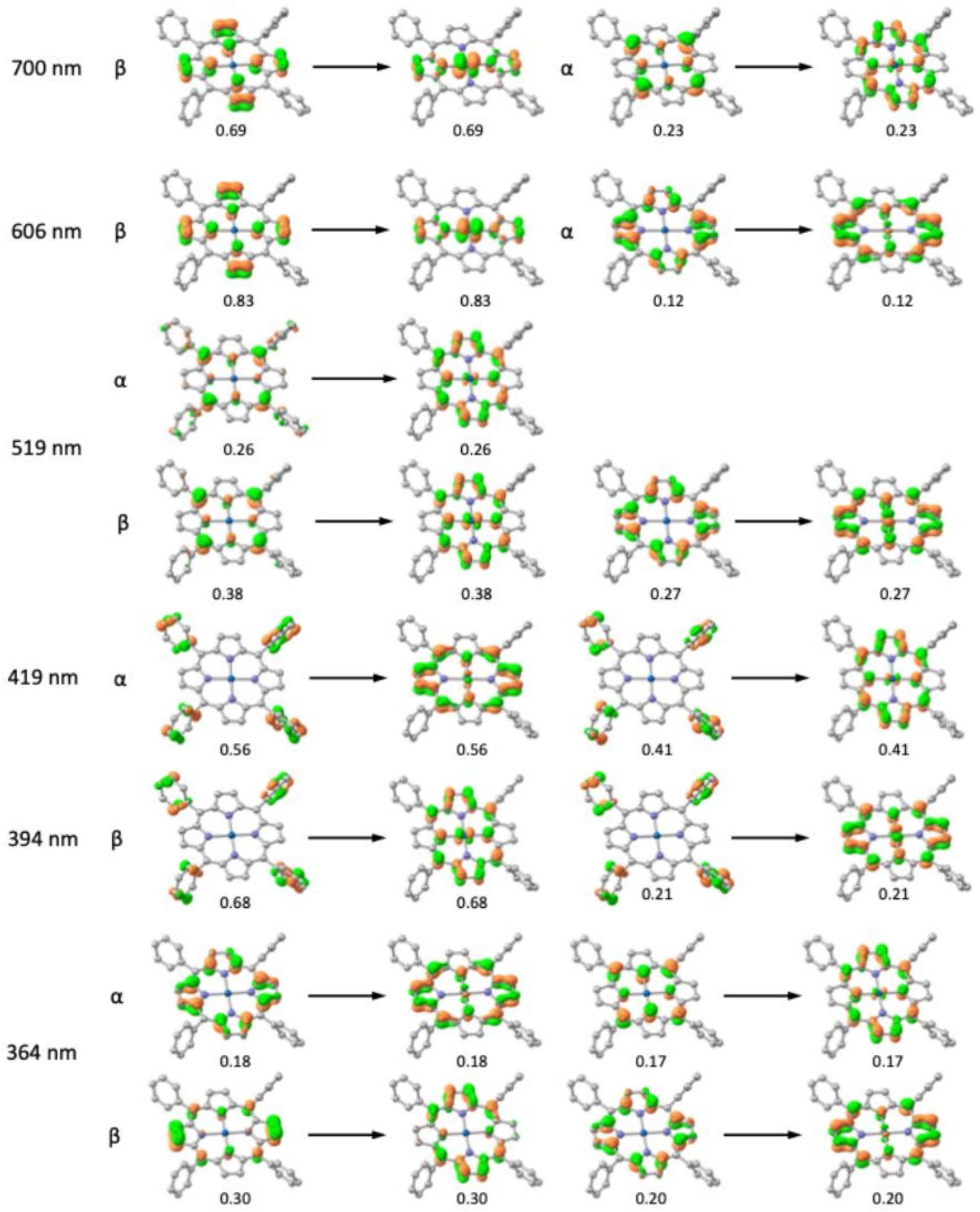

*Scheme S10: Natural transition orbitals contributing to the calculated TD-DFT absorption bands of triplet, cationic (TPP)Ir$^+$ (**10**$^+$) (COSMO-M06L-D4/def2-TZVPP data), isovalue at 0.05 $a_0^{-3/2}$.*

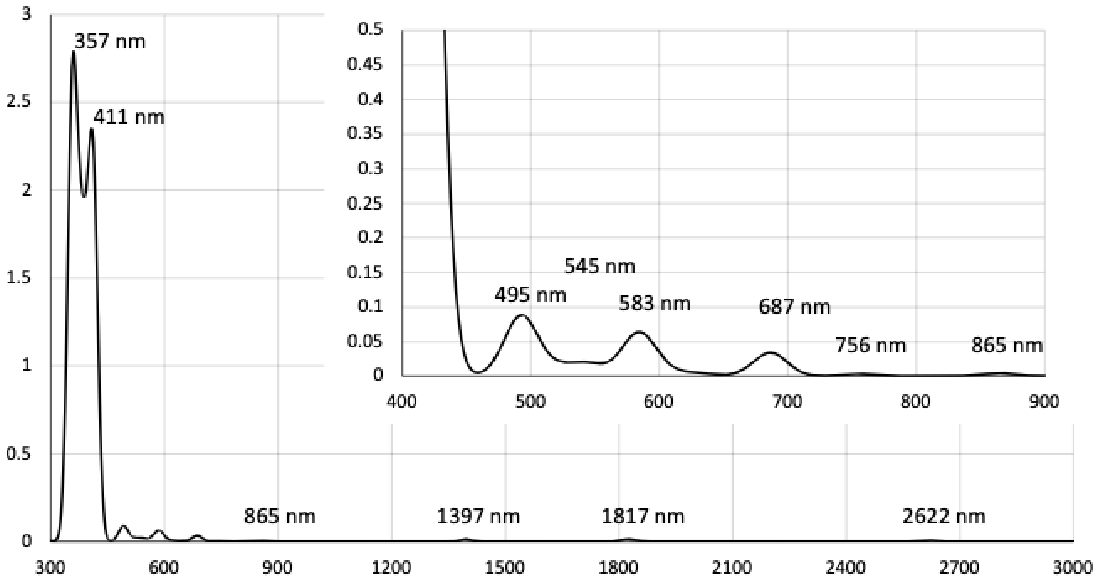

*Figure S71: SO-TD-DFT spectrum of triplet, cationic (TPP)Ir⁺ (**10⁺**), inset: enlarged region from 400 to 900 nm (COSMO-X2C-M06L/TZP data).*

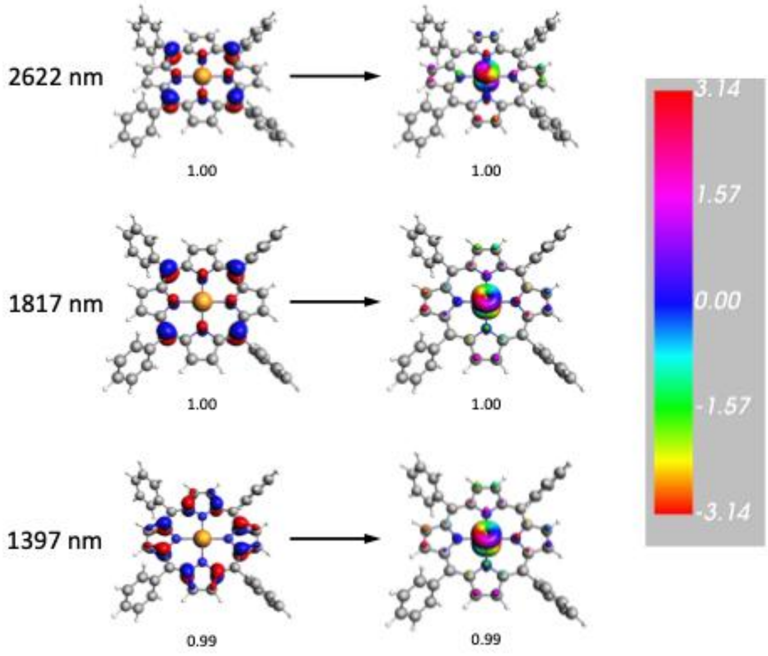

*Scheme S11: Natural transition orbitals contributing to the calculated TD-DFT absorption bands of triplet, cationic (TPP)Ir⁺ (**10⁺**), (COSMO-X2C-M06L/TZP data), scale for phase of the complex NTOs, isovalue at 0.05 $a_0^{-3/2}$.*

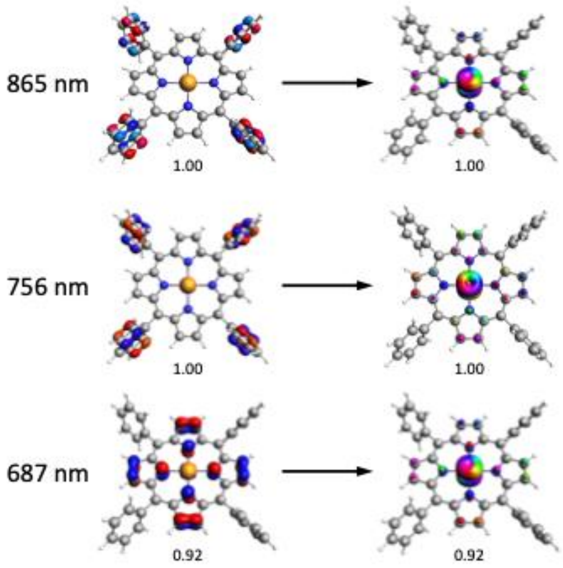

*Scheme S12: (continued): Natural transition orbitals contributing to the calculated TD-DFT absorption bands of triplet, cationic (TPP)Ir⁺ (**10⁺**), (COSMO-X2C-M06L/TZP data), isovalue at 0.05 $a_0^{-3/2}$.*

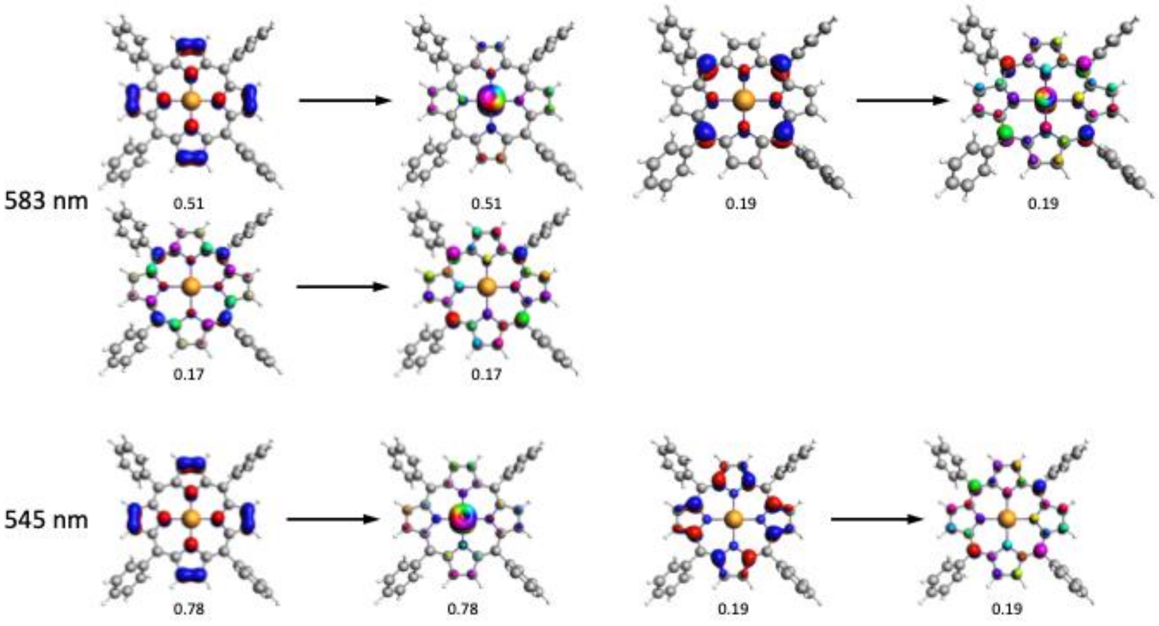

*Scheme S13 (continued): Natural transition orbitals contributing to the calculated TD-DFT absorption bands of triplet, cationic (TPP)Ir⁺ (**10⁺**), (COSMO-X2C-M06L/TZP data), isovalue at 0.05 $a_0^{-3/2}$.*

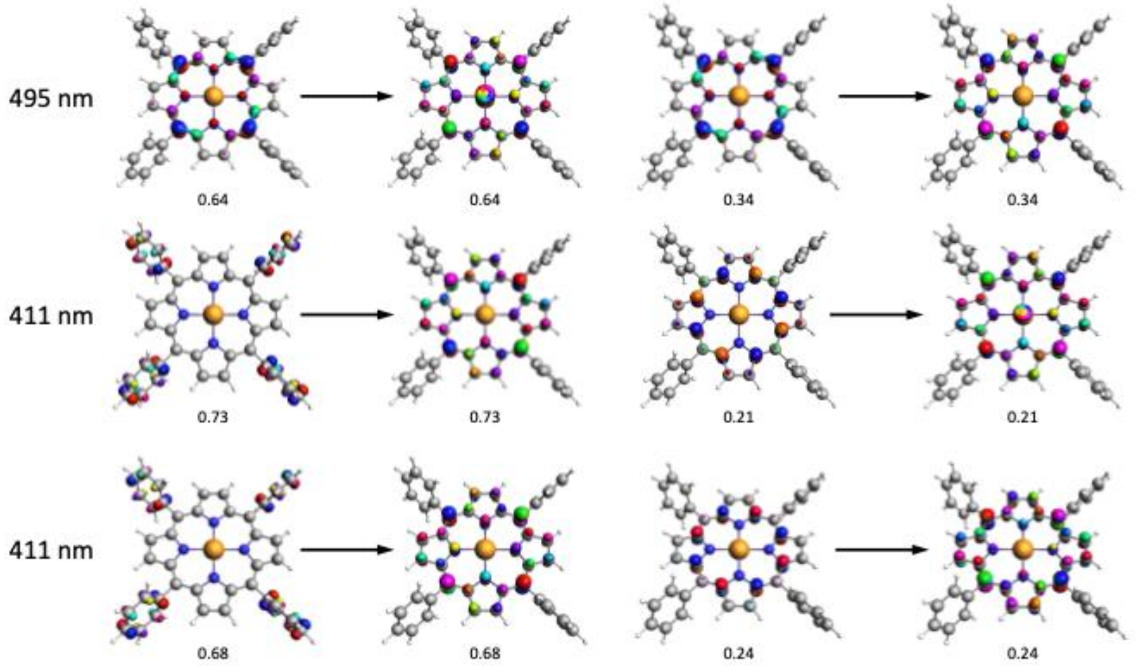

*Scheme S14 (continued): Natural transition orbitals contributing to the calculated TD-DFT absorption bands of triplet, cationic (TPP)Ir^{+} (**10**$^{+}$), (COSMO-X2C-M06L/TZP data), isovalue at 0.05 $a_0^{-3/2}$.*

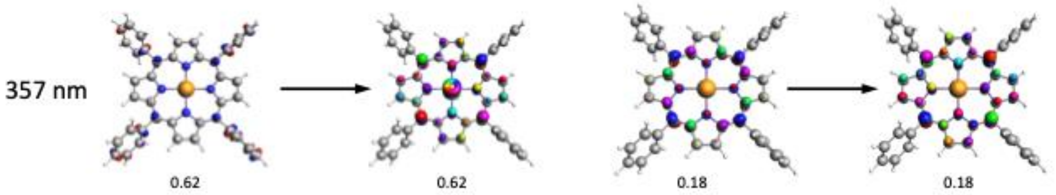

*Scheme S15 (continued): Natural transition orbitals contributing to the calculated TD-DFT absorption bands of triplet, cationic (TPP)Ir^{+} (**10**$^{+}$), (COSMO-X2C-M06L/TZP data), isovalue at 0.05 $a_0^{-3/2}$.*

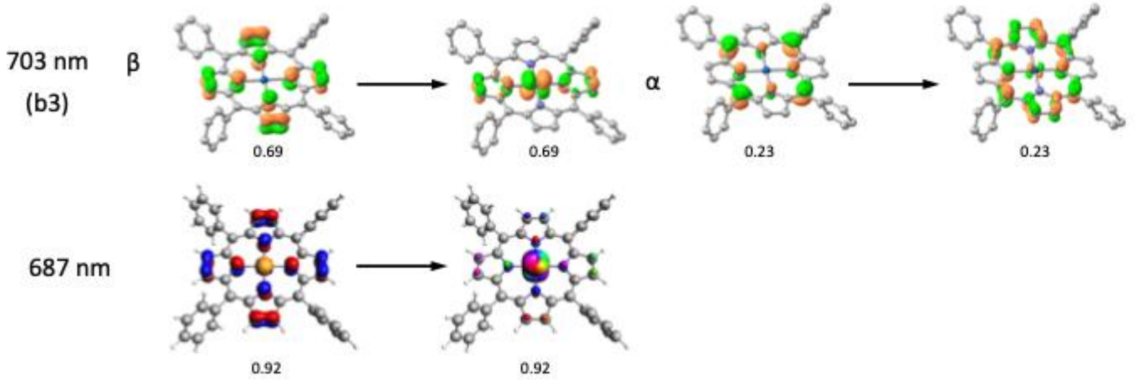

*Scheme S16: Comparison of natural transition orbitals from calculations using an ECP on iridium (top) and spin-orbit relativistic (bottom) relevant for the photoproduct assignment of triplet, cationic (TPP)Ir^{+} (**10**$^{+}$), (COSMO-X2C-M06L/TZP data); isovalue at 0.05 $a_0^{-3/2}$.*

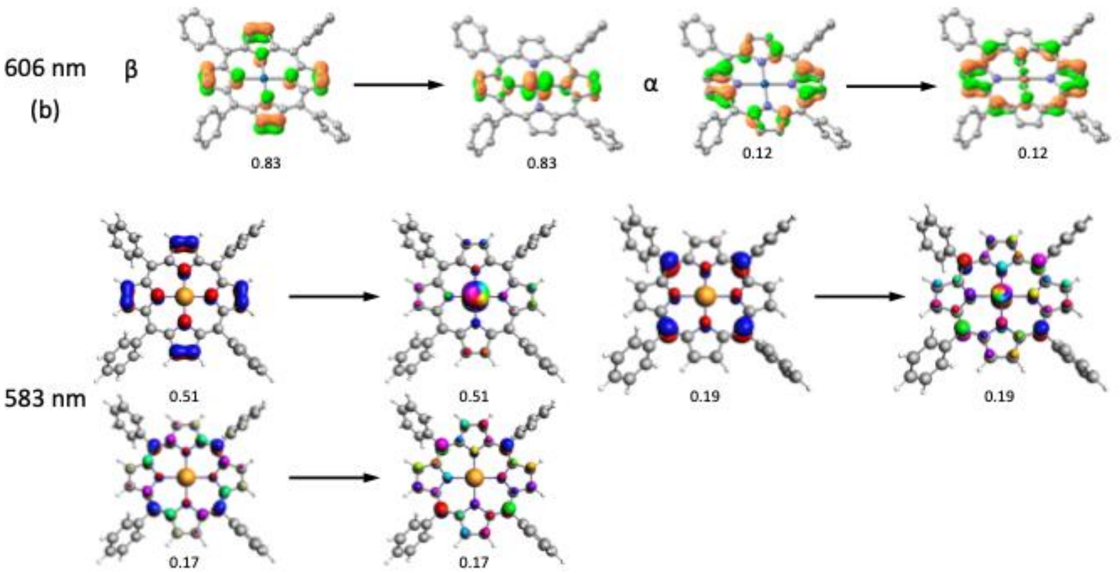

*Scheme S17: Comparison of natural transition orbitals from calculations using an ECP on iridium (top) and spin-orbit relativistic (bottom) relevant for the photoproduct assignment of triplet, cationic (TPP)Ir+ (**10+**), (COSMO-X2C-M06L/TZP data); isovalue at 0.05 $a_0^{-3/2}$.*

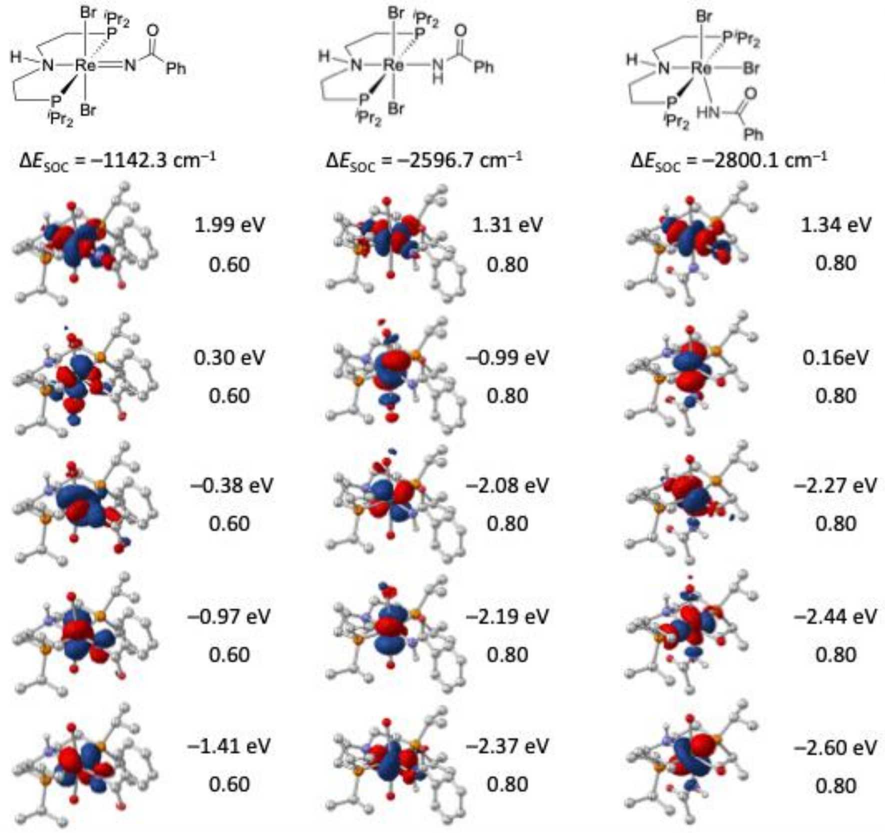

*Scheme S18: Spin orbit stabilization, natural orbitals, occupation numbers and energies for neutral rhenium complexes **3**, the trans isomer of **4** and **4**, state averaged CASSCF calculations with all five 5d rhenium orbitals, averaging over 10 quartets and 40 doublets for **3** and 5 quintets, 45 triplets and 50 singlets for the two isomers of **4**, isovalue at 0.05 $a_0^{-3/2}$.*

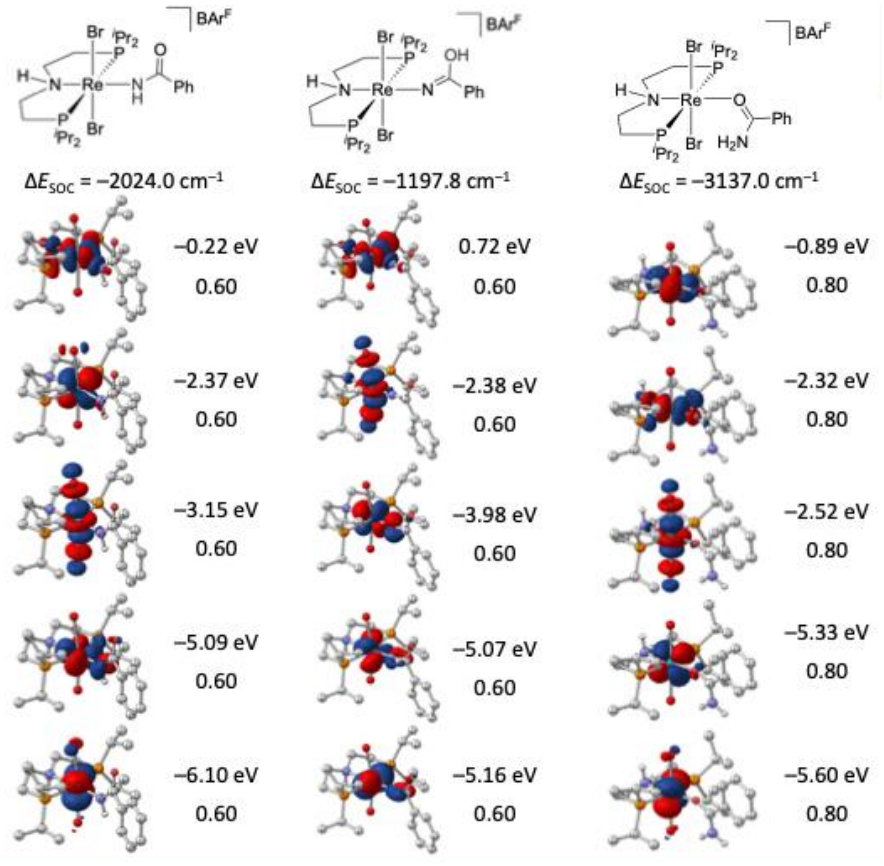

*Scheme S19: : Spin orbit stabilization, natural orbitals, occupation numbers and energies for isomer C of **4**⁺, **4**⁺ and product complex **5**⁺, state averaged CASSCF calculations with all five 5d rhenium orbitals, averaging over 10 quartets and 40 doublets for the isomers of **4**⁺ and 5 quintets, 45 triplets and 50 singlets for **5**⁺, isovalue at 0.05 $a_0^{-3/2}$.*

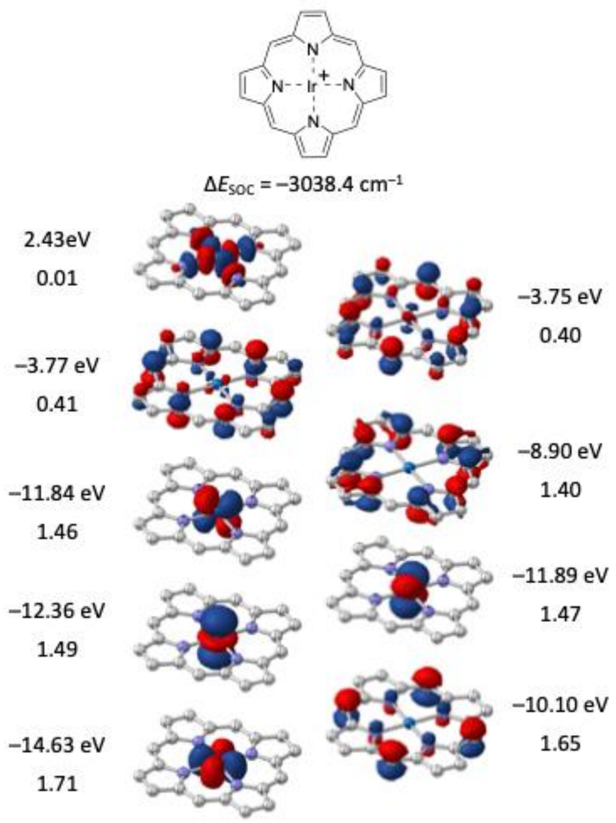

Scheme S20: Spin orbit stabilization, natural orbitals, occupation numbers and energies for the molecular model of complex ***10***$^{+}$*, state averaged CASSCF(10,9) calculation, averaging over 5 quintets, 45 triplets and 50 singlets, isovalue at 0.05* $a_0^{-3/2}$.

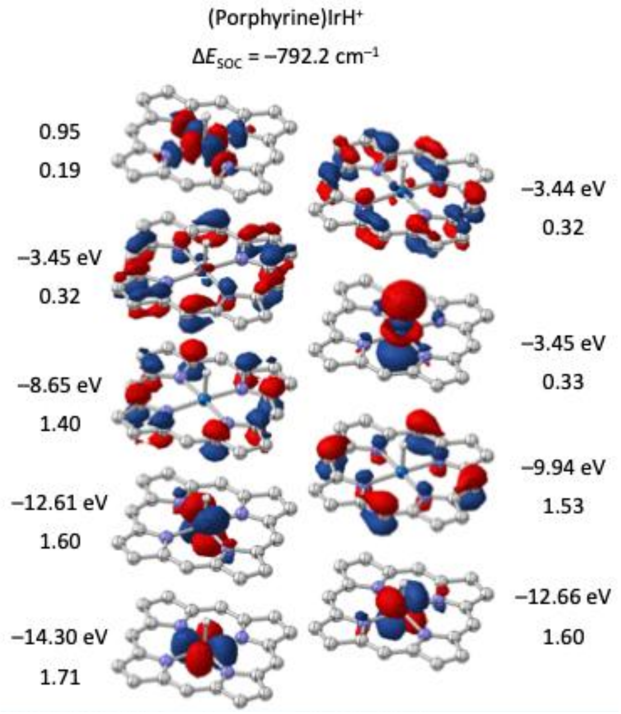

Scheme S21: Spin orbit stabilization, natural orbitals, occupation numbers and energies for the molecular model of complex ***8***$^{+}$*, state averaged CASSCF(9,9) calculation, averaging over 10 quartets and 40 doublets, isovalue at 0.05* $a_0^{-3/2}$.

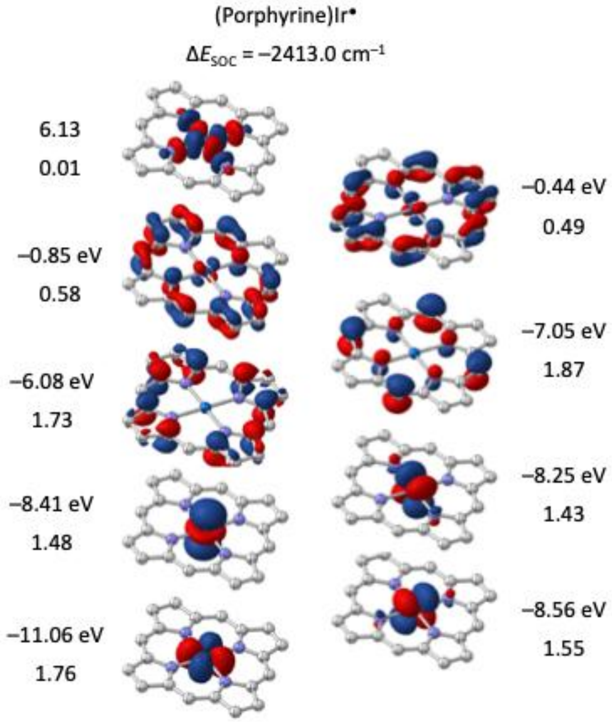

*Scheme S22: Spin orbit stabilization, natural orbitals, occupation numbers and energies for model complex **10**, state averaged CASSCF(11,9) calculation, averaging over 10 quartets and 40 doublets, isovalue at 0.05 $a_0^{-3/2}$.*

6.1 UV/vis spectrum of **3**$^+$

The calculations were carried out with the ORCA 5.0 program suite.[27, 56] The structure was optimized using the PBE0[29,30] functional, Grimme's dispersion correction with Becke-Johnson damping (D3BJ),[20,57] and the chain of spheres approximation[58] (RIJCOSX) to minimize computational costs. Ahlrich's triple-zeta basis set, def2-TZVP, was applied for all heteroatoms (Re, N, O, P, Br) while the smaller def2-SVP basis set was used for all other atoms in combination with the def2/J auxiliary basis set.[19,59,60] Tight convergence criteria were applied both in the self-consistent field and the optimization procedures. The structure was verified as local minimum.

To predict the electronic excitation spectrum, time-dependent DFT within the Tamm-Dancoff approximation[61] was conducted applying the B3LYP[62] functional, the chain of spheres approximation and an increased grid size of 3. Relativistic effects were included within the ZORA approximation[63,49] and spin-orbit coupling between singlets and triplets was considered by using quasi-degenerate perturbation theory.[64] The ZORA-def2-TZVPP all-electron basis set, a recontracted version of the Ahlrich's def2-TZVPP basis set as implemented in ORCA was used for all atoms except Re, for which the SARC-ZORA-TZVPP and SARC/J auxiliary basis set was employed.[50] The influence of the solvent (THF) was taken into account by the conductor-like polarizable continuum model (C-PCM).[65]

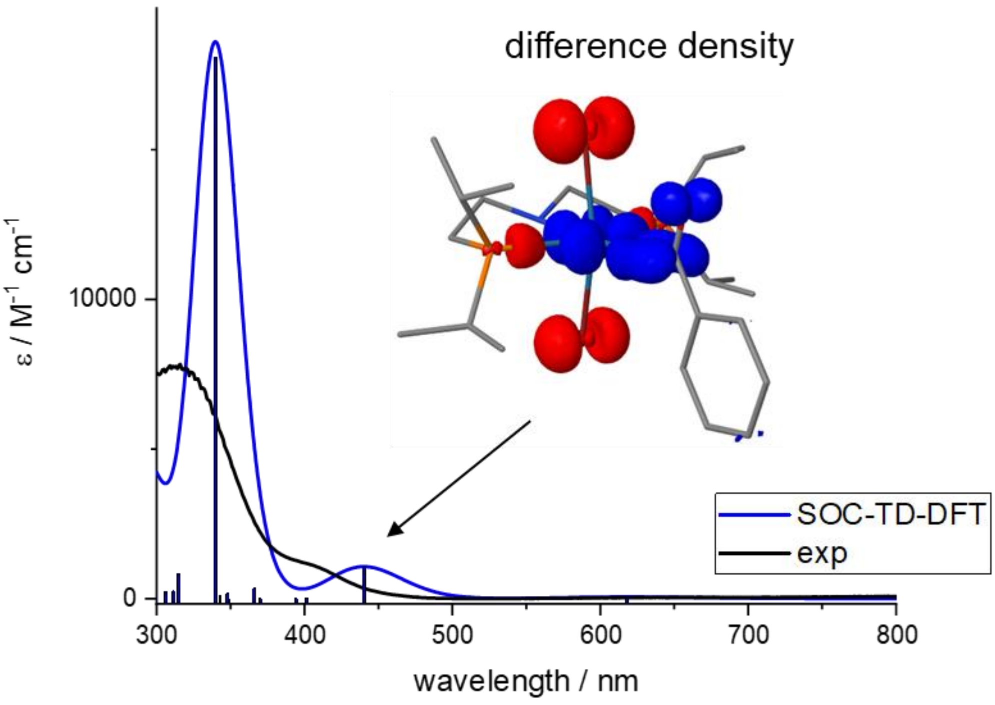

*Figure S72:Experimental UV-vis absorption spectrum of **3(BAr$^F_{24}$)** in THF (black) and TD-DFT computed spectrum (blue). The inset shows the difference density plot for the transition at λ = 432 nm (red: depopulation, blue: population).*

References

1 Fritz, M.; Rupp, S.; Kiene, C. I.; Kisan, S.; Telser, J.; Würtele, C.; Krewald, V.; Schneider, S. Photoelectrochemical Conversion of Dinitrogen to Benzonitrile: Selectivity Control by Electrophile- versus Proton-Coupled Electron Transfer. *Angew. Chem. Int. Ed. Engl.* **2022**, *61*, e202205922.

2 Marsh, D.; Mink, L. Microscale Synthesis and Electronic Absorption Spectroscopy of Tetraphenylporphyrin H_2(TPP) and Metalloporphyrins ZnII(TPP) and NiII(TPP). *J. Chem. Educ.* **1996**, *73* (12), 1188.

3 Kisan, S. Rhenium Mediated Formation of N-Containing Organic Compounds by Nitride Transfer. https://ediss.uni-goettingen.de/handle/11858/14476.

4 Yeung, S. K.; Chan, K. S. Selective Oxidation of (Porphyrinato)iridium(III) Arylethyls by Nitroxide: Evidence for Stabilization of Carbon-Centered Ir−CH 2 −CHR • Radicals by Iridium. *Organometallics* **2005**, *24* (26), 6426–6430. DOI: 10.1021/om050661a.

5 Cheung, C. W.; Chan, K. S. Base-Promoted Selective Activation of Benzylic Carbon−Hydrogen Bonds of Toluenes by Iridium(III) Porphyrin. *Organometallics* **2008**, *27* (13), 3043–3055.

6 Harris, R. K.; Becker, E. D.; Menezes, S. M. C. de; Granger, P.; Hoffman, R. E.; Zilm, K. W. Further conventions for NMR shielding and chemical shifts (IUPAC Recommendations 2008). *Magn. Reson. Chem. (Magnetic resonance in chemistry : MRC)* **2008**, *46* (6), 582–598.

7 Rehm, D.; Weller, A. Kinetics of Fluorescence Quenching by Electron and H-Atom Transfer. *Isr. J. Chem.* **1970**, *8* (2), 259–271.

8 Zanello, P. Inorganic Electrochemistry:Theory, Practice and Application; Royal Society of Chemistry, 2003.

9 Ji, Y.; DiRocco, D. A.; Kind, J.; Thiele, C. M.; Gschwind, R. M.; Reibarkh, M. LED-Illuminated NMR Spectroscopy: A Practical Tool for Mechanistic Studies of Photochemical Reactions. *ChemPhotoChem* **2019**, 3 (10), 984–992.

10 APEX3 v2016.9-0 (SAINT/SADABS/SHELXT/SHELXL), 2016.

11 Sheldrick, G. M. SHELXT–Integrated space-group and crystal-structure determination. *Acta Cryst.* **2015**, A71, 3–8.

12 Sheldrick, G. M. Crystal structure refinement with SHELXL. *Acta Cryst.* **2015**, C71, 3–8.

13 Sheldrick, G. M. A short history of SHELX. *Acta Cryst.* **2008**, A64, 112–122.

14 Spek, A. L. PLATON SQUEEZE: a tool for the calculation of the disordered solvent contribution to the calculated structure factors. *Acta Cryst C* **2015**, 71 (Pt 1), 9–18.

15 Frisch, M. J.; Trucks, G. W.; Schlegel, H. B.; Scuseria, G. E.; Robb, M. A.; Cheeseman, J. R.; Scalmani, G.; Barone, V.; Petersson, G. A.; Nakatsuji, H.; Li, X.; Caricato, M.; Marenich, A. V.; Bloino, J.; Janesko, B. G.; Gomperts, R.; Mennucci, B.; Hratchian, H. P.; Ortiz, J. V.; Izmaylov, A. F.; Sonnenberg, J. L.; Williams-Young, D.; Ding, F.; Lipparini, F.; Egidi, F.; Goings, J.; Peng, B.; Petrone, A.; Henderson, T.; Ranasinghe, D.; Zakrzewski, V. G.; Gao, J.; Rega, N.; Zheng, G.; Liang, W.; Hada, M.; Ehara, M.; Toyota, K.; Fukuda, R.; Hasegawa, J.; Ishida, M.; Nakajima, T.; Honda, Y.; Kitao, O.; Nakai, H.; Vreven, T.; Throssell, K.; Montgomery, Jr., J. A.; Peralta, J. E.; Ogliaro, F.; Bearpark, M. J.; Heyd, J. J.; Brothers, E. N.; Kudin, K. N.; Staroverov, V. N.; Keith, T. A.; Kobayashi, R.; Normand, J.; Raghavachari, K.; Rendell, A. P.; Burant, J. C.; Iyengar, S. S.; Tomasi, J.; Cossi, M.; Millam, J. M.; Klene, M.; Adamo, C.; Cammi, R.; Ochterski, J. W.; Martin, R. L.; Morokuma, K.; Farkas, O.; Foresman, J. B.; Fox, D. J. Gaussian 16 Revision B.01, Wallingford, CT, 2016.

16 Lee,C; Yang, W.; Parr, R. G. Development of the Colle-Salvetti correlation-energy formula into a functional of the electron density. *Phys. Rev. B* **1988**, *37*, 785-789.

17 Becke, A. D. Density-functional exchange-energy approximation with correct asymptotic behavior *Phys. Rev. A* **1988**, *38*, 3098-3100.

18 Becke, A. D. Density-functional thermochemistry. III. The role of exact exchange *J. Chem. Phys.* **1993**, *98*, 5648-5652

19 Weigend, F.; Ahlrichs, R. Balanced basis sets of split valence, triple zeta valence and quadruple zeta valence quality for H to Rn: Design and assessment of accuracy *Phys. Chem. Chem. Phys*. **2005**, *7*, 3297-3305.

20 Grimme, S.; Antony, J.; Ehrlich, S.; Krieg, H. A consistent and accurate *ab initio* parametrization of density functional dispersion correction (DFT-D) for the 94 elements H-Pu *J. Chem. Phys*. **2010**, *132*, 154104

21 Wodyński, A.; Kaupp, M. Local Hybrid Functional Applicable to Weakly and Strongly Correlated Systems *J. Chem. Theory Comput.* **2022**, *18*, 6111-6123.

22 Caldeweyher, E.; Ehlert, S.; Hansen, A.; Neugebauer, H.; Spicher, S.; Bannwarth, C.; Grimme S. A generally applicable atomic-charge dependent London dispersion correction *J. Chem. Phys.* **2019**, *150*, 154122.

23 Furche, F.; Ahlrichs, R.; Hättig, C.; Klopper, W.; Sierka, M.; Weigend F. Turbomole *Wiley Interdiscip. Rev. Comput. Mol. Sci.* **2014**, *4*, 91-100.

24 Franzke, Y. J.; Holzer, C.; Andersen, J. H.; Begušić, T.; Bruder, F.; Coriani, S.; Della Sala, F.; Fabiano, E.; Fedotov, D. A.; Fürst, S.; Gillhuber, S.; Grotjahn, R.; Kaupp, M.; Kehry, M.; Krstić, M.; Mack, F.; Majumdar, S.; Nguyen,v; Parker, S. M.; Pauly, F.; Pausch, A.; Perlt, E.; Phun, G. S.; Rajabi, A.; Rappoport, D.; Samal, B.; Schrader, T.; Sharma, M.; Tapavicza, E.; Treß, R. S.; Voora, V.; Wodyński, A.; Yu, J. M.; Zerulla, B.; Furche, F.; Hättig, C.; Sierka, M.; Tew, D. P.; Weigend, F. TURBOMOLE: Today and Tomorrow *J. Chem. Theory Comput.* **2023**, *19*, 6859-6890.

25 Ariai, J.; Gellrich, U. The entropic penalty for associative reactions and their physical treatment during routine computations *Phys. Chem. Chem. Phys.* **2023**, *25*, 14005-14015.

26 Gerlach, T. ; Müller, S.; de Castilla, A. G.; Smirnova, I. An open source COSMO-RS implementation and parameterization supporting the efficient implementation of multiple segment descriptors *Fluid Phase Equilibria* **2022**, *560*, 113472.

27 Neese, F. The ORCA program system *Wiley Interdiscip. Rev. Comput. Mol. Sci.* **2012**, *2*, 73-78.

28 Neese, F. Software update: The ORCA program system—Version 5.0 *Wiley Interdiscip. Rev. Comput. Mol. Sci.* **2022**, *12*, e1606.

29 Perdew, J. P.; Burke, K.; Ernzerhof, M. Generalized Gradient Approximation Made Simple *Phys. Rev. Lett.* **1996**, *77*, 3865–3868.

30 Adamo, C.; Barone, V. Toward reliable density functional methods without adjustable parameters: The PBE0 model *J. Chem. Phys*. **1999**, *110*, 6158–6170.

31 Adler, T. B.; Knizia, G.; Werner, H.-J. A simple and efficient CCSD(T)-F12 approximation *J. Chem. Phys.* **2007**, *127*, 221106.

32 Knizia, G.; Adler, T. B.; Werner, H.-J. Simplified CCSD(T)-F12 methods: theory and benchmarks *J. Chem. Phys.* **2009**, *130*, 054104.

33 Werner, H.-J.; Knowles, P. J.; Knizia, G.; Manby, F. R.; Schütz, M. Molpro: a general-purpose quantum chemistry program package *Wiley Interdiscip. Rev. Comput. Mol. Sci.* **2012**, *2*, 242-253.

34 Peterson, K. A.; Adler, T. B.; Werner, H.-J. Systematically convergent basis sets for explicitly correlated wavefunctions: the atoms H, He, B-Ne, and Al-Ar *J. Chem. Phys.* **2008**, *128*, 084102.

35 Figgen, D.; Peterson, K. A.; Dolg, M.; Stoll, H. Energy-consistent pseudopotentials and correlation consistent basis sets for the 5d elements Hf-Pt *J. Chem. Phys.* **2009**, *130*, 164108.

36 Yousaf, K. E.; Peterson, K. A. Optimized auxiliary basis sets for explicitly correlated methods *J. Chem. Phys.* **2008**, *129*, 184108.

37 Kritikou, S.; Hill, J. G. Auxiliary Basis Sets for Density Fitting in Explicitly Correlated Calculations: The Atoms H-Ar *J. Chem. Theory Comput.* **2015**, *11*, 5269-5276.

38 Weigend, F. Hartree-Fock exchange fitting basis sets for H to Rn *J. Comput. Chem.* **2008**, *29*, 167-175.

39 Hill, J. G. Auxiliary basis sets for density fitting second-order Møller-Plesset perturbation theory: correlation consistent basis sets for the 5d elements Hf-Pt *J. Chem. Phys.* **2011**, *135*, 044105.

40 Zhao, Y.; Truhlar, D. G. A new local density functional for main-group thermochemistry, transition metal bonding, thermochemical kinetics, and noncovalent interactions *J. Chem. Phys.* **2006**, 125, 194101.

41 Klamt, A.; Schüürmann, G. COSMO: a new approach to dielectric screening in solvents with explicit expressions for the screening energy and its gradient *J. Chem. Soc., Perkin Trans. 2* **1993**, 799-805.

42 Svensson, M.; Humbel, S.; Froese, R. D. J.; Matsubara, T.; Sieber, S.; Morokuma, K. ONIOM: A Multilayered Integrated MO + MM Method for Geometry Optimizations and Single Point Energy Predictions. A Test for Diels−Alder Reactions and Pt(P(t -Bu) 3) 2 + H 2 Oxidative Addition *J. Phys. Chem.* **1996**, *100*, 19357-19363.

43 Svensson, M.; Humbel, S.; Morokuma, K. Energetics using the single point IMOMO (integrated molecular orbital+molecular orbital) calculations: Choices of computational levels and model system *J. Chem. Phys.* **1996**, *105*, 3654-3661.

44 Chung, L. W.; Sameera, W. M. C.; Ramozzi, R.; Page, A. J.; Hatanaka, M. Petrova, G. P.; Harris, T. V.; Li, X.; Ke, Z.; Liu, F.; Li, H.-B.; Ding, L.; Morokuma, K. The ONIOM Method and Its Applications *Chem. Rev.* **2015**, *115*, 5678-5796.

45 Angeli, C; Cimiraglia, R.; Evangelisti, S.; Leininger, T.; Malrieu, J.-P. Introduction of n-electron valence states for multireference perturbation theory *J. Chem. Phys.* **2001**, 114, 10252–10264.

46 Angeli, C.; Cimiraglia, R.; Malrieu, J.-P. N-electron valence state perturbation theory: a fast implementation of the strongly contracted variant *Chem. Phys. Lett.* **2001**, 350, 297-305.

47 Angeli, C.; Cimiraglia, R.; Malrieu, J.-P. n-electron valence state perturbation theory: A spinless formulation and an efficient implementation of the strongly contracted and of the partially contracted variants *J. Chem. Phys.* **2002**, 117, 9138–9153.

48 Heß, B. A.; Marian, C. M.; Wahlgren, U.; Gropen, O. A mean-field spin-orbit method applicable to correlated wavefunctions *Chem. Phys. Lett.* **1996**, 251, 365-371.

49 Van Wüllen, C. Molecular density functional calculations in the regular relativistic approximation: Method, application to coinage metal diatomics, hydrides, fluorides and chlorides, and comparison with first-order relativistic calculations *J. Chem. Phys.* **1998**, 109, 392–399.

50 Pantazis, D. A.; Chen, X.-Y.; Landis, C.R.; Neese, F. All-Electron Scalar Relativistic Basis Sets for Third-Row Transition Metal Atoms *J. Chem. Theory Comput.* **2008**, 4, 6, 908–919.

51 Weigend, F. A fully direct RI-HF algorithm: Implementation, optimised auxiliary basis sets, demonstration of accuracy and efficiency *Phys. Chem. Chem. Phys*. **2002**, 4, 4285-4291.

52 Bordwell, F. G.; Harrelson, J. A. Jr., Lynch, T. Y. Homolytic bond dissociation energies for the cleavage of .alpha.-nitrogen-hydrogen bonds in carboxamides, sulfonamides, and their derivatives. The question of synergism in nitrogen-centered radicals *J. Org. Chem.* **1990**, *55*, 3337-3341.

53 Resa, S.; Millán, A.; Fuentes, N.; Crovetto, L.; Luisa Marcos, M.; Lezama, L.; Choquesillo-Lazarte, D.; Blanco, V.; Campaña, A. G.; Cárdenas, D. J.; Cuerva, J. M. O-H and (CO)N-H bond weakening by coordination to Fe(ii) *Dalton Trans.* **2019**, *48*, 2179-2189.

54 Wise, C. F.; Agarwal, R. G.; Mayer, J. M. Determining Proton-Coupled Standard Potentials and X–H Bond Dissociation Free Energies in Nonaqueous Solvents Using Open-Circuit Potential Measurements *J. Am. Chem. Soc.* **2020**, *142*, 10681-10691.

55 Agarwal, R. G.; Coste, S. C.; Groff, B. D.; Heuer, A. M.; Noh, H.; Parada, G. A.; Wise, C. F.; Nichols, E. M.; Warren, J. J.; Mayer, J. M. Free Energies of Proton-Coupled Electron Transfer Reagents and Their Applications *Chem. Rev.* **2022**, *122*, 1-49.

56 Neese, F. Software Update: The ORCA Program System,Version 4.0. Wiley Interdiscip. *Rev. Comput. Mol. Sci.* **2018**, *8*, e1327.

57 Grimme, S.; Ehrlich, S.; Goerigk, L. Effect of the damping function in dispersion corrected density functional theory *J. Comput. Chem*. **2011**, *32*, 1456–1465.

58 Neese, F.; Wennmohs, F.; Hansen, A.; Becker, U. Efficient, approximate and parallel Hartree–Fock and hybrid DFT calculations. A 'chain-of-spheres' algorithm for the Hartree–Fock exchange *Chemical Physics* **2009**, *356*, 98-109.

59 Weigend, F. Accurate Coulomb-fitting basis sets for H to Rn *Phys. Chem. Chem. Phys*. **2006**, *8*, 1057.

60 Andrae, D.; Häußermann, U.; Dolg, M.; Stoll, H.; Preuß, H. Energy-adjustedab initio pseudopotentials for the second and third row transition elements *Theor. Chim. Acta* **1990**, *77*, 123–141.

61 Neese, F.; Olbrich, G. Efficient use of the resolution of the identity approximation in time-dependent density functional calculations with hybrid density functionals *Chem. Phys. Lett.* **2002**, *362*, 170−178.

62 Stephens, P. J.; Devlin, F. J.; Chabalowski, C. F.; Frisch, M. J. Ab Initio Calculation of Vibrational Absorption and Circular Dichroism Spectra Using Density Functional Force Fields *J. Phys. Chem.* **1994**, *98*, 11623.

63 van Lenthe, E.; Baerends, E. J.; Snijders, J. G. Relativistic regular two-component Hamiltonians *J. Chem. Phys.* **1993**, *99*, 4597–4610.

64 de Souza, B.; Farias, G.; Neese, F.; Izsak, R. Predicting Phosphorescence Rates of Light Organic Molecules Using Time-Dependent Density Functional Theory and the Path Integral Approach to Dynamics *J. Chem. Theory Comput*. **2019**, *15*, 1896.

65 Barone, V.; Cossi, M. Quantum Calculation of Molecular Energies and Energy Gradients in Solution by a Conductor Solvent Model *J. Phys. Chem. A*, **1998**, *102*, 1995.
